# The effects of functional response and host abundance fluctuations on genetic rescue in parasitoids with single‐locus sex determination

**DOI:** 10.1002/ece3.6889

**Published:** 2020-10-14

**Authors:** Etsuko Nonaka, Veijo Kaitala

**Affiliations:** ^1^ Department of Ecology, Environment, and Plant Science Stockholm University Stockholm Sweden; ^2^ Organismal and Evolutionary Biology Programme University of Helsinki Helsinki Finland; ^3^ Present address: Department of Biological and Environmental Science University of Jyväskylä Jyväskylä Finland

**Keywords:** dispersal, eco‐evolutionary dynamics, genetic drift, individual‐based model, population bottleneck, spatially structured population

## Abstract

Many parasitoids have single‐locus complementary sex determination (sl‐CSD), which produces sterile or inviable males when homozygous at the sex determining locus. A previous study theoretically showed that small populations have elevated risks of extinction due to the positive feedback between inbreeding and small population size, referred to as the diploid male vortex. A few modeling studies have suggested that the diploid male vortex may not be as common because balancing selection at sex determining loci tends to maintain high allelic diversity in spatially structured populations. However, the generality of the conclusion is yet uncertain, as they were drawn either from models developed for particular systems or from a general‐purpose competition model. To attest the conclusion, we study several well‐studied host–parasitoid models that incorporate functional response specifying the number of attacked hosts given a host density and derive the conditions for a diploid male vortex in a single population. Then, we develop spatially structured individual‐based versions of the models to include female behavior, diploid male fertility, and temporal fluctuations. The results show that producing a handful of successful offspring per female parasitoid could enable parasitoid persistence when a typical number of CSD alleles are present. The effect of functional response depends on the levels of fluctuations in host abundance, and inviable or partially fertile diploid males and a small increase in dispersal can alleviate the risk of a diploid male vortex. Our work supports the generality of effective genetic rescue in spatially connected parasitoid populations with sl‐CSD. However, under more variable climate, the efficacy of the CSD mechanism may substantially decline.

## Graphical abstract

Many parasitoids have single‐locus complementary sex determination (sl‐CSD), which produces sterile or inviable males when homozygous at the sex determining locus. It appears to have a dire consequence on the persistence of small populations by inducing an extinction vortex. Our work supports the generality of effective genetic rescue in spatially connected parasitoid populations with sl‐CSD.

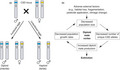

## 1 INTRODUCTION

**Figure 1:**
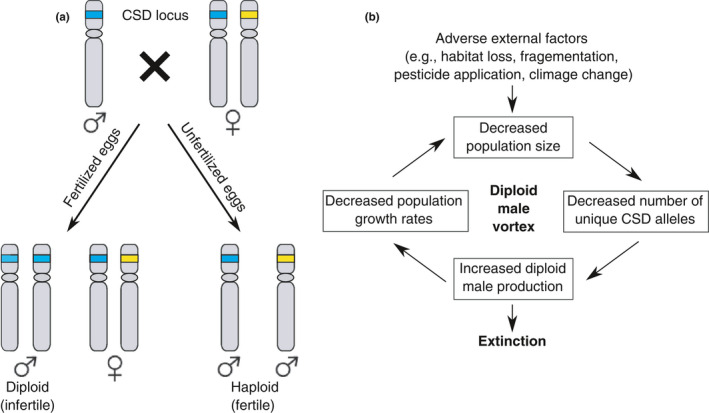
(a) A schematic diagram of the inheritance of the single‐locus complementary sex determination (sl‐CSD) system. Modified from figure 1 in Nair et al. (2018). (b) External factors can drive populations to become small, which could trigger the negative feedback between small population size and diploid male production (due to increased inbreeding), potentially leading to extinction in an extinction vortex. A diploid male vortex is an extinction vortex caused by diploid male production in hymenopteran populations. Modified from figure 3a in Zayed and Packer (2005)

Small populations are susceptible to inbreeding and reduced population growth rate caused by the positive feedback between small population size and inbreeding depression, known as the extinction vortex (Gilpin & Soule, 1986). This positive feedback may occur in a more exacerbated form in hymenopteran taxa (sawflies, bees, wasps, and ants), which have the haplodiploid genetic system with complementary sex determination (CSD; Heimpel & de Boer, 2008). In these taxa, unfertilized haploid eggs develop into males, while fertilized diploid eggs develop into females only if they are heterozygous at the CSD loci (Figure 1a; Heimpel & de Boer, 2008). Diploid eggs that are homozygous at the CSD loci develop as diploid males. Diploid males are often infertile or inviable (hereafter DMs; Cowan & Stahlhut, 2004; Heimpel & de Boer, 2008; Whiting, 1943). Because inbreeding increases homozygosity and hence can produce excess infertile or inviable individuals in taxa carrying CSD loci, their populations consequently can become vulnerable to a decline due to inbreeding depression. In many species, CSD involves only one locus (single‐locus CSD or sl‐CSD; Harpur et al., 2012; van Wilgenburg et al., 2006). Zayed and Packer (2005) theoretically demonstrated that, using a single population model, excess production of DMs may cause an extinction vortex in small populations with sl‐CSD, which they called the diploid male vortex (Figure 1b). The study concluded that DM production can elevate the risk of extinction by ten or more folds and alerted conservation management to substantially higher extinction risks of small hymenopteran populations with sl‐CSD.

Empirical evidence of the diploid male vortex in literature is, however, limited, and existing studies provide suggestive but inconclusive evidence (de Boer et al., 2015; Boff et al., 2014; Nair et al., 2018; Soro et al., 2016; Takahashi et al., 2008; Zayed & Packer, 2001). There could be inherent difficulties in documenting a diploid male vortex “in action” over several generations before the population goes extinct. The fragility of populations with sl‐CSD suggested by Zayed and Packer (2005) seems also counter to the facts that over 80 species of hymenopterans with known sl‐CSD exist in nature (Harpur et al., 2012; Heimpel & de Boer, 2008; van Wilgenburg et al., 2006) and that hymenopterans are one of the most specious taxa (Gaston, 1991; Godfray, 1994). Previous modeling studies have shown that a diploid male vortex may not occur as readily in nature because migration and negative frequency‐dependent selection can maintain allelic variation at the CSD loci in spatially structured populations (Hein et al., 2009; Nair et al., 2018) and because coupled dynamics of host and parasitoid populations dampen unstable oscillations (Bompard et al., 2016). Hein et al. (2009) found that, using a general competition model, initiation of a diploid male vortex would require stringent life history traits (e.g., low fertility of DMs, almost no dispersal), as seemingly small changes in these traits could boost parasitoid persistence. Two other studies examined particular systems with empirically parameterized models and likewise reported resilience of parasitoid populations (Nair et al., 2018; Weis et al., 2017). Nair et al. (2018) reported that, although the proportion of DMs increased by threefold in the population of a parasitoid after the host populations crashed, their modeling results indicated little imminent risk of a diploid male vortex even if the host fluctuates with similar variability. Investigation using other types of models can attest the generality of these conclusions.

Population ecologists have developed a number of parasitoid models that incorporate “functional response” to model the rate of parasitism (Hassell, 2000). Functional response in parasitoid models determines how the number of hosts attacked (and parasitized) by parasitoids depends on host density and is limited by parasitoids’ ability to search and handle hosts (Hassell, 2000). These models with functional response can therefore encode parasitoid biology more explicitly than a general competition model used by Hein et al. (2009). They used a logistic growth model and thus assumed that the parasitoids were host limited and attacked hosts randomly. Parasitoids can also be egg or time limited, which motivated the development of functional response models (Hassell, 2000). Because the shapes of functional response reflect how the number of attacked hosts responds as a function of host density, the models incorporating different types of functional responses are suited to investigate the effects of host population size on parasitoid populations and their persistence. In particular, because population bottlenecks can cause genetic drift and loss of CSD alleles, how parasitoid and host densities relate at their low densities likely influences persistence of parasitoid populations with CSD. In addition, fluctuations in host abundance can be introduced into these models to study the effects of population bottlenecks and recoveries on the frequencies of extinctions. More variable and correlated climatic fluctuations are anticipated under future climate change scenarios, and higher variability and prolonged or spatially extensive population bottlenecks (Jangjoo et al., 2020; Kahilainen et al., 2018; Tack et al., 2015) could increase the vulnerability of populations with sl‐CSD.

In this study, we examine parasitoid population models involving six different functional responses and life history characteristics to study their effects on population persistence. Our approach combines population dynamics models and population genetics in attempt to better understand the population‐level consequences of sex determination genetics. We also consider the effects of environmental fluctuations through host abundance. To this end, we analyze simple deterministic models of a single population and then individual‐based models of spatially structured populations incorporating demographic stochasticity and various parasitoids’ reproductive and behavioral characteristics. Our results show that negative frequency‐dependent selection and dispersal in spatially structured populations enable effective genetic rescue and that modest changes in life history characteristics could alleviate extinction risk substantially. In agreement with previous modeling studies mentioned above, natural parasitoid populations may be more resistant to a diploid male vortex than originally suggested based on results obtained from a single population model (Zayed & Packer, 2005). However, population persistence could become less certain when large fluctuations in host abundance cause prolonged population bottlenecks in parasitoids. This study suggests that the maintenance of habitat connectivity should be an effective conservation strategy for parasitoid populations with sl‐CSD in fragmented landscapes under anticipated more extreme and variable climate.

## 2 METHODS

### 2.1 The deterministic single population model

We assume solitary parasitoids. Females randomly mate only once, males can mate multiple times (Godfray, 1994; Zayed, 2004). DMs can mate but do not transfer functional sperm (Harpur et al., 2012; but later we relax this assumption in the simulation model). Females mated with a DM can still have sons via parthenogenesis, and all her daughters will be inviable triploids (van Wilgenburg et al., 2006) and immediately die as eggs so that the host becomes available for other parasitoids to parasitize. Therefore, DMs not only “waste” their mothers’ efforts to produce daughters but also their mates’ reproductive efforts. The population dynamics of parasitoids are described by

$${\mathrm{DP}}_{t+1}=cN_{t}\left(1‐s_{0}\right)b_{t}{\tilde{M}}_{t}$$

$${\mathrm{HP}}_{t+1}={cN_{t}s}_{0}b_{t}$$

where ${\mathrm{DP}}_{t}$ and ${\mathrm{HP}}_{t}$ are the densities of diploid and haploid individuals at time t, respectively. c is the expected number of parasitoids emerging per parasitized host, and $N_{t}$ is the density of the host at time t. $s_{0}$ is the primary (i.e., mothers’ intended) sex ratio (females can control how many eggs to fertilize in haplodiploid species), ${\tilde{M}}_{t}$ is the fraction of normal males among all males, $\frac{M_{t}}{M_{t}+{\mathrm{DM}}_{t}}$,$M_{t}$ is the number of male parasitoids, and $b_{t}$ is a function of $F_{t}$, the number of female parasitoids and signifies the rate of parasitism (fraction of hosts being parasitized).

We consider six well‐studied parasitoid models presented in Table 2.1 in Hassell (2000; Tables 1 and 2), including functional responses type I, II, and III and two modes of attacks to be contrasted to assess the effects of positive slopes, saturation, and convexity at low host densities. Model 1 assumes that parasitoids are egg‐limited (pro‐ovigenic; females emerge with a fixed complement of eggs), while others assume they are never egg‐limited (synovigenic; mature eggs throughout their life; Godfray, 1994). Models 1, 2, 4, and 6 assume that eggs are randomly distributed among host individuals, implying all host individuals are equally susceptible to parasitism (Hassell, 2000). These functional forms assume a Poisson process in parasitoids’ finding hosts, implying that a host can be attacked multiple times. That is, females cannot tell apart parasitized and not‐yet parasitized hosts. We assume that one parasitoid at most comes out of one host (i.e., c=1; Table 2). In Models 3 and 5, all host individuals are not equally susceptible to parasitism and an overall distribution of parasitoid attacks is more aggregated than random, as modeled by the negative binomial distribution. Aggregation of attacks can occur when the probability of finding hosts by parasitoids is biased in space (e.g., due to habitat heterogeneity or searching behavior), certain genotypes are more resistant to parasitism, or when hosts are spatially aggregated (e.g., on host plants or microhabitats (Godfray, 1994; Hassell & May, 1985)). With the extra clumping parameter k, the random and aggregated attack models are not directly comparable to each other except the case when k approaches infinity. Models 2 and 3 take on type I (linear without an asymptote) functional response of parasitoids, while Models 4 and 5 take on type II (hyperbolic) and Model 6 type III (sigmoidal; Table 1). Type II and type III functional responses assume that parasitoids are time limited so that any parasitoid cannot indefinitely increase the number of attacked hosts as in type I (the functions saturate at $\frac{1}{T_{h}}$). The number of attacked hosts does not change with host density in Model 1 (no functional response). The relative numbers of attacked hosts switch among the functional responses as the density of female parasitoids increases at low host densities (Figure S1). Therefore, depending on the duration and severity of bottlenecks these functional responses differentially affect parasitoid persistence.

**Table 1:** The definitions of the symbols, state variables, and parameters

Symbols used	Definition	Values used in the IBM	Source
$F_{t}$	Number of female parasitoids at time t	State variable (positive integer)	
$DM_{t}$	Number of diploid males at time t	State variable (positive integer)	
$M_{t}$	Number of normal males at time t	State variable (positive integer)	
$\bar{N}$	Mean host population size across patches	100	Hein et al. (2009)
${\bar{N}}_{j}$	Mean host population size in patch j	Drawn randomly from a uniform distribution between 10 and 190	Hein et al. (2009)
$N_{j,t}$	Host population size in patch j at time t	Drawn randomly from the lognormal distribution with $Normal\left(log\left({\bar{N}}_{j}\right),\sigma_{j}\right)$	Hein et al. (2009)
w	Number of eggs to become reproducing adults, laid per female parasitoids (Model 1 only)	[3, 4, 5]	[3, 4, 5, 6, 7, 8, 9] in the supplementary materials
a	Per capita searching efficiency (“area of encounter”) defined as the proportion of total hosts encountered and parasitized by one female per life time (Model 2–6)	[0.03, 0.035, 0.04, 0.045, 0.05]	Hassell (2000) [0.025, 0.03, 0.035, 0.04, 0.045, 0.05] in the supplementary materials
c	The number of parasitoids emerging per parasitized host	1	Hassell (2000)
k	Clumping parameter for negative binomial distributions (Models 3 and 5)	1	Hassell and May (1985), Hassell (2000), May (1978)
$T_{h}$	Handling time per attacked prey (Models 4–6) expressed as a fraction of a life time and corresponds to the length of the time interval between consecutive eggs laid	0.005 (200 eggs laid in a life time)	Zhang et al. (2004), Zamani et al. (2006), Fathipour et al. (2017), Pourtaghi et al. (2017)
d,g	Fitting parameters (Model 6 only)	Varied such that $a=\frac{d{\bar{N}}_{\mathrm{all}}}{1+g{\bar{N}}_{\mathrm{all}}}$ varies between 0.03 and 0.05, where ${\bar{N}}_{\mathrm{all}}$ is mean host density among all the patches (=100)	
$s_{0}$	Primary sex ratio (fraction of haploid)	0.5	
ℓ	The number of CSD alleles	20 initially	Cook and Crozier (1995)
h	Fraction of diploid homozygote	$\frac{1}{\ell }$ at equilibrium	Yokoyama and Nei (1979); van Wilgenburg et al. (2006); Harpur et al. (2012)
m	Probability of dispersal	Varied between 0 and 0.4	
ρ	Correlation between patches for the variance–covariance matrix	0 or 0.2	Nair et al. (2018)
$\sigma_{j}$	Standard deviation of the underlying normal distribution for temporal fluctuation in patch j	0 (no fluctuation), $0<\sigma_{j}\leq 0.5$ (small fluctuation), or $0<\sigma_{j}\leq 1.2$ (large fluctuation)	Nair et al. (2018)
κ	Color of the environmental noise	0.6 (red noise)	Kaitala et al. (1997)

**Table 2:** The expressions of $b_{t}b_{t}$ for the six models, functional response, distributions of attacks, and the persistence conditions for a parasitoid population

	$b_{t}$(the rate of parasitism)	Functional response	Distribution of attacks	Condition for parasitoid persistence expressed in terms of $h^{*}=\frac{1}{\ell }$ and $s_{0}$
1	$b_{t}=\left(1‐e^{‐\frac{wF_{t}}{N_{t}}}\right)$	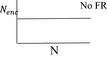	Random	$w>\frac{1+\sqrt{1+4h^{*}\left(\frac{1}{s_{0}}‐1\right)}}{2c\left(s_{0}‐1\right)\left(h^{*}‐1\right)}$
2	$b_{t}=\left(1‐e^{‐aF_{t}}\right)$	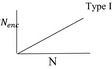	Random	$aN>\frac{1+\sqrt{1+4h^{*}\left(\frac{1}{s_{0}}‐1\right)}}{2c\left(s_{0}‐1\right)\left(h^{*}‐1\right)}$
3	$b_{t}=\left(1‐{\left(1+\frac{aF_{t}}{k}\right)}^{‐k}\right)$		Negative binomial (aggregated)	
4	$b_{t}=\left(1‐e^{‐\frac{aF_{t}}{1+aT_{h}N_{t}}}\right)$	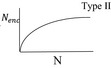	Random	$aN>\frac{1+\sqrt{1+4h^{*}\left(\frac{1}{s_{0}}‐1\right)}}{2c\left(s_{0}‐1\right)\left(h^{*}‐1\right)‐\left(1+\sqrt{1+4h^{*}\left(\frac{1}{s_{0}}‐1\right)}\right)T_{h}}$
5	$b_{t}=\left(1‐{\left(1+\frac{aF_{t}}{k\left(1+aT_{h}N_{t}\right)}\right)}^{‐k}\right)$		Negative binomial (aggregated)	
6	$b_{t}=\left(1‐e^{‐\frac{dN_{t}F_{t}}{1+gN_{t}+dT_{h}N_{t}^{2}}}\right)$ $a=\frac{dN_{t}}{1+gN_{t}}$	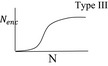	Random	

Note

See Table 1 for the definition of the parameters and symbols. In the second column, the shapes of the function responses are shown schematically. $N_{\mathrm{enc}}$ = the number of hosts encountered (and attacked). N= host density. Notice that the right‐hand side of the persistence conditions are the same for the first three models.

Diploid offspring are either females (if heterozygous at the CSD locus) or DMs (if homozygous). We partition ${\mathrm{DP}}_{t+1}$ into females or DMs by calculating the proportion of homozygotes among offspring, $h_{t+1}$;

$$h_{t+1}=\sum_{i=1}^{l}p_{i}^{2}\left(t+1\right)$$

in a population at the Hardy–Weinberg equilibrium and $p_{i}\left(t+1\right)=\frac{2*DP\left(t+1\right)*p_{i,dp}\left(t+1\right)+HP\left(t+1\right)*p_{i,hp}\left(t+1\right)}{\sum_{j=1}^{l}2*DP\left(t+1\right)*p_{j,dp}\left(t+1\right)+HP\left(t+1\right)*p_{j,hp}\left(t+1\right)}$ where $p_{i,dp}$ and $p_{i,hp}$ are the proportion of i allele in the diploid and haploid population, respectively. According to the model of the temporal dynamics of CSD allele frequencies, all l alleles have the frequency equal to 1/l at the equilibrium (Harpur et al., 2012; van Wilgenburg et al., 2006; Yokoyama & Nei, 1979). We then split the equation of ${\mathrm{DP}}_{t+1}$ into $F_{t+1}$ (females) and ${\mathrm{DM}}_{t+1}$ (diploid males), and relabel ${\mathrm{HP}}_{t+1}$ as $M_{t+1}$ (males),

$$F_{t+1}={\mathrm{DP}}_{t+1}\left(1‐h_{t+1}\right)=cN_{t}\left(1‐s_{0}\right)b_{t}{\tilde{M}}_{t}\left(1‐h_{t+1}\right)$$

$${\mathrm{DM}}_{t+1}={\mathrm{DP}}_{t+1}h_{t+1}=cN_{t}\left(1‐s_{0}\right)b_{t}{\tilde{M}}_{t}h_{t+1}$$

$$M_{t+1}={cN_{t}s}_{0}b_{t}$$

### 2.2 The individual‐based simulation model of a spatially structured parasitoid population

We extend the deterministic model above to an individual‐based simulation model where it is more straightforward to incorporate spatial structure, parasitoid life history and behavior, and fluctuations in host abundance. Females and DMs are represented by two copies of CSD alleles, and (haploid) males are by one allele. They are initialized at the start of the simulation by randomly drawing integers from the uniform distribution between 1 and the maximum number of alleles present.

The space consists of 64 patches arranged in an 8 x 8 lattice, j ^th^ of which contains a host population $\left(N_{j,t}\right)$whose mean abundance $\left({\overset{‐}{N}}_{j}\right)$is drawn from a uniform distribution between 10 and 190 individuals (mean across all the patches, $\overset{‐}{N},$equals to 100; Table 1). The temporal dynamics of host abundance in each patch is determined independently randomly, unless specified, from the lognormal distribution with the mean of the underlying normal distribution equal to $log\left({\overset{‐}{N}}_{j}\right)$and the standard deviation $\left(\sigma_{j}\right)$equal to 0 (i.e., no temporal variation nor variation in $\sigma_{j}$among patches), uniformly randomly distributed in ${0<\sigma }_{j}\leq 0.5$(i.e., temporal variation and variation in $\sigma_{j}$among patches) or in ${0<\sigma }_{j}\leq 1.2$across patches. Thus, the host dynamics are independent of the parasitoid. When host abundance is spatially autocorrelated, $log\left(N_{j,t}\right)$is drawn from the multivariate normal distribution with mean host abundance $log\left({\bar{N}}_{j}\right)$. and a variance–covariance matrix with $\sigma_{j}$ in the diagonal and the correlation parameter ρ = 0.2. $\sigma_{j}$ is uniformly randomly distributed in $0<\sigma_{j}\leq 1.2$. These values follow empirically observed ranges in a previously studied system (Nair et al., 2018). Temporally autocorrelated host abundance is generated by assuming a multiplicative effect of the noise

$$d_{j,t+1}=\kappa d_{j,t}+\text{Normal}\left(0,\sigma_{j}\right)$$

$$N_{j,t+1}={\bar{N}}_{j}+d_{j,t+1}$$

(Kaitala et al., 1997). $d_{j,t}$ is colored environmental noise in patch j at time t, κ controls the color of the noise (when κ>0 red noise (positive autocorrelation), when κ<0 blue noise (negative)). We set κ= 0.6. $\sigma_{j}$ is again uniformly randomly distributed in $0<\sigma_{j}\leq 1.2$.

Parasitoids can disperse once to one of the neighboring 4 patches in cardinal directions with a specified probability $\left(m\right)$, and dispersing parasitoids are randomly chosen every generation. After dispersal, the rate of parasitism $\left(b_{t}\right)$ and the number of parasitized hosts are calculated for each patch. The number of offspring (including inviable DMs) to be produced is the same as the number of parasitized hosts (i.e., c=1). Reproducing females are randomly selected from the females in the patch, are assigned to one or more hosts to parasitize, and randomly select one male or DM to mate. Male and DMs can be chosen multiple times. No sib‐mating avoidance is assumed as evidence is scarce (Collet et al., 2020; van Wilgenburg et al., 2006). For each reproducing female, the number of daughters among her offspring is determined randomly with probability $1‐s_{0}$. Daughters inherit one allele randomly selected out of two from their mothers and the other one from their fathers. Sons inherit one randomly chosen allele from their mothers. Females mating with sterile DMs only produce sons, while those with partially fertile DMs can produce some daughters (i.e., if a female mated with a 20% fertile DM, 20% of eggs to be daughters will be randomly assigned to actually become daughters, and the rest will be inviable triploids). The alleles in the offspring can mutate to a brand new or another allele already in the populations, but we set mutation rate at zero for this study. Diploids that are homozygous at the CSD locus either become sterile or partially fertile DMs, eliminated as inviable DMs, or stay as females if CSD is not operating.

We consider six DM scenarios that are similar to what Hein et al. (2009) considered. The reference level is provided by the scenario where all diploids are females (“No CSD”). The viability and sterility of DMs are varied in three scenarios where they are inviable (die immediately; “inviable DM”), effectively sterile (participate in mating but do not transfer functional sperm; “sterile DM”), and 20% fertile (only 20% of sired eggs are diploid, not triploid; “partially fertile DM”; Heimpel & de Boer, 2008; van Wilgenburg et al., 2006). In the last two scenarios, we vary female behavior; the ability to reject DMs with 20% probability (“reject DM 20%”) and the preference for female‐biased primary sex ratio (“sex ratio 40/60”; Antolin et al., 2003; Cook & Crozier, 1995; Godfray, 1994).

We run the simulations for 5,000 generations to determine whether the parasitoid populations can persist for a sufficiently long period of time. The populations are initialized with 5,000 parasitoids and 20 CSD alleles. We set c=1 for computational efficiency and therefore consider only successful eggs laid to kill the host (i.e., no host escaping parasitism nor dying from other causes, or no triploid eggs). We vary dispersal rate from 0 to 0.4, w from 3 to 5 (Model 1), and aa from 0.03 to 0.05 (Model 2–6). We select these values of w and a to compare Model 1 with the rest of the models (the mean host abundance in the landscape is set at 100 so that these values of a yield 3 to 5 successful eggs per female parasitoid on average). In the absence of host abundance fluctuation, parasitoid populations mostly persist the above values of w and a in Models 1 and 2. The results for wider ranges of w and a are presented in the supplementary materials. We run 20 replicates of each unique parameter combination. The parasitoid populations are considered extinct if the numbers of females or normal haploid males become zero before the end of the 5000‐generation simulations. Persistence is calculated as the number of simulations with persistent parasitoid populations divided by the number of replicates, 20. Allelic richness is the number of unique CSD alleles in the entire metapopulation and averaged across 20 replicates. Persistence and allelic richness are averaged over all the simulations across the values of w or a and dispersal rate or averaged by dispersal rate.

## 3 RESULTS

### 3.1 The deterministic single population model

We conduct a stability analysis of the extinction equilibrium $\left(F,M,DM\right)=\left(0,0,0\right)$ of this system to derive expressions for deterministic extinction of the parasitoid population. We observe in numerical simulation of this model that the fraction of normal (haploid) males, $\tilde{M}$, and the fraction of homozygotes, h, approach a constant value as F→0,M→0, and DM→0. We can, therefore, express the equilibrium value of $\tilde{M}$ at the extinction equilibrium as

$${\tilde{M}}^{*}=\frac{M^{*}}{M^{*}+DM^{*}}=\frac{s_{0}}{s_{0}+\left(1‐s_{0}\right){\tilde{M}}^{*}h^{*}}{\tilde{M}}^{*}$$

where $M^{*},DM^{*},{\tilde{M}}^{*}$, and $h^{*}$ are equilibrium values of respective state variables. Faria et al. (2016) arrived at an equivalent expression with different parameterization. We can solve the quadratic equation for ${\tilde{M}}^{*}>0$ and evaluate the derivative of F as F→0 to discern the stability of F at F=0. For Model 1, the extinction equilibrium is stable if.

$${\tilde{M}}^{*}<\frac{1}{wc\left(1‐s_{0}\right)\left(1‐h^{*}\right)}$$

**Figure 2:**
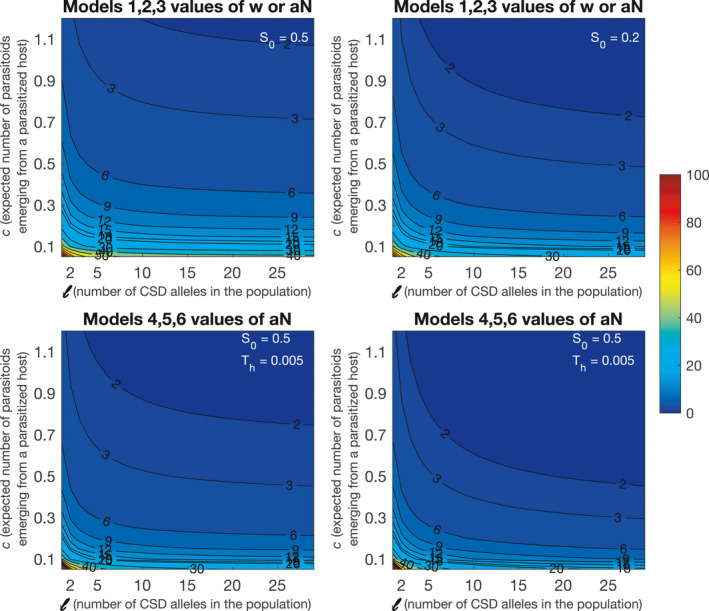
The minimum number of successful offspring needed to be produced for parasitoid persistence (Table 1) evaluated at various values of c (expected number of parasitoids emerging from a parasitized host) and l (number of alleles) at two values of primary sex ratio (fraction of haploid males, $s_{0}$), for Models 1–3 (a and b) and for Models 4–6 (c–f). For the latter set of models, the conditions are evaluated at $T_{h}$=0.005 (a parasitoid can attack up to 200 hosts per lifetime)

Or equivalently,

$${\tilde{\mathrm{DM}}}^{*}>1‐\frac{1}{wc\left(1‐s_{0}\right)\left(1‐h^{*}\right)}$$

because ${\tilde{M}}^{*}+{\tilde{\mathrm{DM}}}^{*}=1.$ Therefore, when the fraction of DMs exceeds this quantity, the extinction equilibrium is stable. Otherwise, the parasitoid population is to persist. For the unrealistically stringent case of two CSD alleles the equilibrium value of $h,h^{*},$ is 0.5. Assuming $s_{0}=0.5$ and c=1, the minimum w for persistence is 6, which is one to two orders of magnitude lower than a potential number of eggs to be typically laid by a female parasitoid (Figure 2a; Cheng et al., 2017; Harvey, 2007; Harvey et al., 2001, 2017; Heimpel et al., 1998; Houston et al., 1992; Vos & Hemerik, 2003; Waage & Ming, 1984). In natural hymenopteran populations, the number of CSD alleles normally ranges from 9 to 20 (Cook & Crozier, 1995), and our result suggests that 3 or more viable offspring per female could sustain population persistence (Figure 2a). A large fraction of parasitized hosts will die from causes other than parasitism or kill parasitoid eggs or larvae via immune responses (e.g., encapsulation) so that likely c<1 in nature (for solitary parasitoids laying one egg per host). For instance, in the case with c=0.1, 30, or more successfully attacked hosts per female would suffice to sustain the parasitoid population. Parasitoid populations can be strongly female biased (Godfray, 1994), and if we assume $s_{0}=0.2$, it requires fewer successful offspring to attain persistence (Figure 2b). Similarly, for Model 2 and 3, we obtain the persistence condition (Table 2) and find that the two conditions are equivalent when w=aN. The condition becomes more complex when handling time $\left(T_{h}\right)$ is included in the model (Model 4–6; Table 1, Figure 2c,d). The result elucidates that the persistence of the parasitoid population depends on the population size of the host in Models 2–6, but not in Model 1.

### 3.2 Individual‐based simulation model

**Figure 3:**
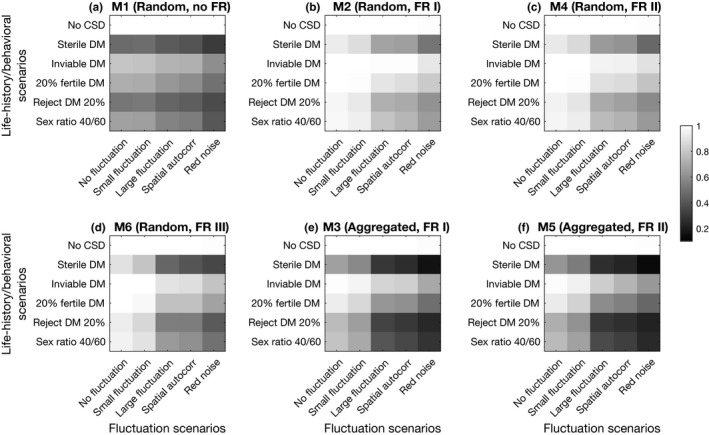
The proportion of simulation runs that persisted for 5,000 generations. In each panel, the six DM life history scenarios are in rows, and the fluctuation scenarios in the columns. For each parameter set, 20 simulations are run. To produce this figure, simulated data with $w=\left[3,4,5\right]$ or $a\bar{N}=\left[3,4,5\right]$ are aggregated across all dispersal rates from 0 to 0.4. Red noise = temporally positively autocorrelated host abundance

We set up the parameter values so that, when CSD is not operating, populations very rarely go extinct in any of the scenarios (2 out of 1,000s of simulations). The persistence of parasitoid populations is least sensitive to increasing levels of fluctuations in host abundance and their autocorrelation when the model does not include functional response (Model 1; Figure 3a). It is most sensitive in the aggregated attack models (Models 3 and 5; Figure 3e,f). Persistence is similar between the models with type I (Models 2 and 3, Figure 3b,e) and type II functional response (Models 4 and 5 Figure 3c,f) within either mode of attacks. The random attack model with functional response type III (Model 6; Figure 3d) achieves lower persistence than those with type I or type II. Persistence is the lowest when DMs are sterile in all scenarios. When DMs are inviable or partially fertile, persistence improves, in many cases, substantially (by up to almost 60%; Figure 3).

**Figure 4:**
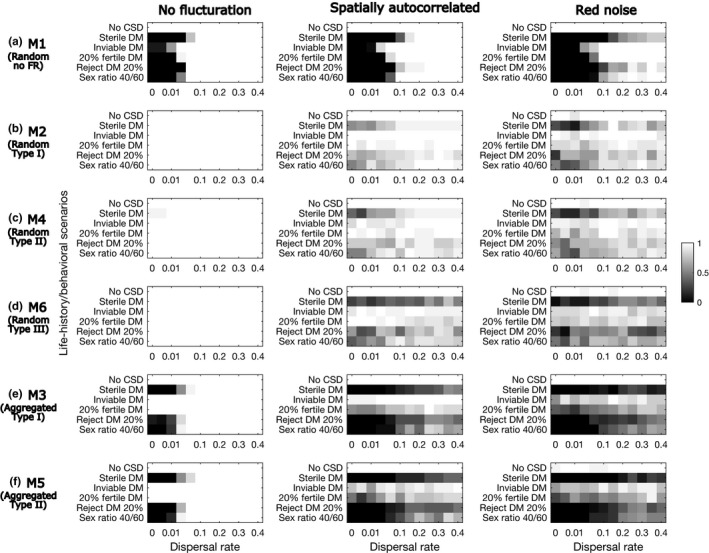
The proportion of simulation runs (out of 20) that persisted for 5,000 generation with increasing dispersal rate. The six models are shown in the rows and three fluctuation scenarios in the columns (the other scenarios are in the online supplementary materials). In each panel, the six DM scenarios are in the rows. The dispersal rate varies from 0 to 0.4 (0, 0.005, 0.01, 0.02, 0.05, 0.1, 0.15, 0.2, 0.25, 0.3, 0.35, 0.4). The parameter values are w = 4 (Model 1) and $a\bar{N}$=4 (Models 2–6)

Population persistence of parasitoids increases with dispersal rate for the majority of the scenarios (Figure 4). Across all the models and fluctuation scenarios, persistence increases more quickly with increasing dispersal rate in the inviable and partially fertile DM scenarios than in the other scenarios. As the amplitudes of fluctuations increase, more dispersal is needed to achieve comparable levels of persistence. Compared with the random attack models with type I and type II functional response (Figure 4b,c), the model with type III (Figure 4d) shows slower increases in persistence with increasing dispersal. The aggregated attack models (Models 3 and 5; Figure 4e,f) show slower increases in persistence with increasing dispersal than the random attack models (Models 2, 4, 6; Figure 4b–d).

**Figure 5:**
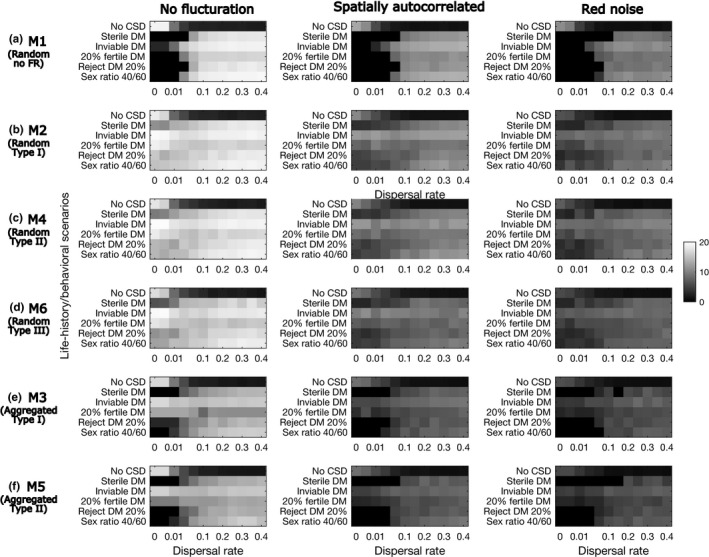
The number of unique alleles in the 64 patches remaining after 5,000 generations with increasing dispersal rate. The six models are shown in the rows and three fluctuation scenarios in the columns (the other scenarios are in the online supplementary materials). In each panel, the six DM scenarios are in rows. The dispersal rate varies from 0 to 0.4 (0, 0.005, 0.01, 0.02, 0.05, 0.1, 0.15, 0.2, 0.25, 0.3, 0.35, 0.4). The parameter values are w = 4 (Model 1) and $a\bar{N}$=4 (Models 2–6). Each simulation is initiated with 20 unique alleles (no mutation)

The number of alleles maintained at the CSD locus with increasing dispersal rate mirrors the patterns of population persistence (Figure 5). Because the locus is neutral when no CSD is operating, allelic diversity is quickly lost with increasing dispersal, as expected. Random attacks (Figure 5b–d) lead to greater numbers of maintained alleles for a given fluctuation and DM scenario than aggregated attacks (Figure 5e,f). When fluctuation is large and spatially or temporally autocorrelated, the number of maintained alleles drops in all the models and scenarios (the second and third column). These results show that strong balancing selection at the CSD locus causes newly arriving alleles via immigrants to increase in frequency and restores lost allelic diversity from population bottlenecks. In other words, negative density‐dependent selection at the CSD locus enables efficient genetic rescue in the population. However, extensive (i.e., spatially autocorrelated) and prolonged (i.e., red noise) bottlenecks can deplete allelic diversity and hence increase the chance of parasitoid extinction via a diploid male vortex.

## 4 DISCUSSION

By studying models of parasitoid populations with functional response that links host abundance and the number of attacked (and parasitized) hosts, we explore how the persistence of parasitoids with sl‐CSD can be affected by population dynamics, behavioral and life history traits, and inbreeding. First, we confirmed analytically previous results from simulations by Zayed and Packer (2005) that in a large population a handful of successful offspring produced per female enable parasitoid persistence when a typical number of CSD alleles (9 to 20; Cook & Crozier, 1995) are present. Second, type I and type II functional response behave similarly within each mode of attacks (random or aggregated). The random attack models with no (flat) or type III functional response show lower persistence of parasitoid populations across the DM or fluctuation scenarios than type I or type II random attack models. Persistence decreases markedly in the type III random attack model when amplitudes of fluctuations are large. Third, all the DM scenarios, inviable and partially fertile DMs in particular, increase parasitoid persistence, relative to the sterile DM case, invariably across the models and fluctuation scenarios. Lastly, a small fraction of dispersing parasitoids enables effective genetic rescue among fragmented populations. Negative density‐dependent selection at the CSD locus in combination with dispersal enables genetic rescue, where newly arriving alleles carried by immigrants increase in frequency and restore allelic diversity in genetically depauperate local populations (Antolin et al., 2003; Nair et al., 2018). Overall, our study extends the findings from the previous study by Hein et al. (2009) to models with density‐dependent attacks and to autocorrelated fluctuations of host abundance.

### 4.1 Model assumptions

We assumed that the host population was independent of the parasitoid to have the focus of the analysis on parasitoid persistence, as we were interested in how parasitoid populations respond to host density could affect the frequency of the diploid male vortex. This situation may occur when host abundance is determined by factors other than parasitoids such as abiotic conditions or resource availability (e.g., Nair et al., 2018). Decoupling the populations may have stabilized the dynamics, as coupled host–parasitoid models such as the Nicholson‐Bailey model (our Model 2 with a coupled host; Hassell, 2000) is typically unstable. Interestingly, a recent study showed that sl‐CSD stabilized dynamics and promoted parasitoid persistence in a coupled model (Bompard et al., 2016). In our (noncoupled) models, sl‐CSD exerted purely negative effects. These two modeling approaches are complementary, but it is interesting that both generally point to less ominous effects of sl‐CSD on population persistence than it may have seemed initially.

We limited the dispersal of parasitoids to once in lifetime to one of the neighboring local populations. Because many parasitoids are known to be good dispersers, this assumption may seem restrictive. We envisioned that the spatial extent scales with dispersal ability. If the parasitoid is highly mobile, a local population likely occupies a large area, which may render dispersal to another population infrequent. If the parasitoid is sedentary, dispersal is rare. Longer dispersal distance could cause CSD alleles to be permanently lost during a bottleneck by random drift from the entire population, if the gene flow becomes too high. Somewhat limited dispersal allows local populations to maintain different sets of alleles and a larger set as a whole metapopulation.

### 4.2 Interactions between population dynamics and the population genetics of sl‐CSD

Fluctuations in population size in time and space influence how much genetic diversity is maintained and how it is distributed among local populations in spatially structured populations (Jangjoo et al., 2020; Varvio et al., 1986; Whitlock, 1992). In populations going through a population bottleneck, the amounts of heterozygosity and alleles lost depend on the severity of the bottleneck (population size at a bottleneck) and population growth rate (Nei et al., 1975). Functional response determines the number of hosts attacked, hence the number of emerging parasitoids, at given parasitoid and host densities. Therefore, it determines the size of a parasitoid population in a bottleneck and how quickly they can grow when host abundance subsequently increases. Type I and type II functional response allow parasitoid populations to achieve higher numbers of attacked hosts at low host densities than type III functional response. As a result, parasitoid populations governed by type III functional response experience severer population bottlenecks and take longer to recover, raising the chances for losing CSD alleles by genetic drift. Moreover, parasitoid populations can take advantage of high host density when the number of attacked hosts increases with host density, allowing populations to grow large and accumulate genetic diversity. This explains why the model with no functional response leads to lower persistence of parasitoid populations. These population dynamical effects of functional response contribute to reducing extinction risk under certain conditions, hence the occurrence of the diploid male vortex, caused by the negative feedback between small population size and genetic drift.

Our analytical results suggest that a small number of successful parasitoid offspring per female suffice to sustain the persistence of a large parasitoid population with a typical number of CSD alleles found in natural populations. This agrees with the results from Zayed and Packer (2005) and Hein et al. (2009). Female parasitoids would need to lay multiple times more eggs than these numbers because many host species have defense mechanisms to kill parasitoid eggs or developing larvae and many parasitized hosts likely die of other causes. Even when postoviposition mortality is taken into account, the numbers of eggs laid needed for parasitoid persistence seem attainable by a female parasitoid in normal conditions as they usually produce hundreds up to a couple of thousands of eggs in lifetime (Waage & Ming, 1984; Houston et al., 1992; Godfray, 1994; Heimpel et al., 1998; Harvey et al., 2017, 2001; Harvey, 2007; Vos & Hemerik, 2003; Cheng et al., 2017). If this conjecture is realistic, although sl‐CSD appears fragile and “unintelligent” (sensu van Wilgenburg et al., 2006) at the first sight, the system seems to work quite well at the population level. A meta‐analysis found that experimental studies most commonly reported type II functional response with parasitoids (Fernández‐arhex & Corley, 2003). Although population bottlenecks can eliminate rare alleles, rare alleles have weaker effects on heterozygosity at the population level as far as the remaining alleles occur in intermediate frequencies (Nei et al., 1975). Also, decline in heterozygosity after a bottleneck is less when populations can recover quickly (Maruyama & Fuerst, 1985; Nei et al., 1975).

The models with random and aggregated attacks are not directly comparable because of the additional parameter k. Only if k approaches infinity, the aggregated attack models converge to the random attack model with the same functional response. We assume k=1, based on the studies that reported the values of k between 0.28 to 1.3 (Hassell, 2000; Hassell & May, 1985; May, 1978). In the simulations the functional responses in the aggregated attack models attain lower number of attacked hosts at intermediate female parasitoid densities than in the random attack models (Figure S1). There are multiple reasons known for unequal susceptibility to parasitism among hosts (hence aggregated attacks) in natural populations, and unequal susceptibility is probably more realistic than hosts all being equally susceptible (Hassell, 2000). If parasitoids are able to select high quality hosts to attack, the aggregated attack models can be considered to have already discounted the number of attacked hosts for loss of offspring from host mortality and immunological defense to some degree. If this is the case, the discrepancy between the number of successful offspring and the number of eggs needed to be laid to compensate for lost offspring should be smaller for aggregated attacks than for random attacks.

### 4.3 Life history and behavioral scenarios

In all simulation scenarios, inviable and partially fertile DMs mitigate extinction risk the most, while rejecting DM 20% of the time and female‐biased primary sex ratio improve population persistence to much lesser extent. These results qualitatively agree with previous studies (Fauvergue et al., 2015; Hein et al., 2009; Zayed, 2004; Zayed & Packer, 2005). Sterile DMs not only fail their mothers’ reproductive efforts to produce daughters but also waste their mates’ such efforts. Inviable DMs die without mating (i.e., no wasting of their mates). Parasitoid populations where females reject DMs 20% of the time persist much less than those where females mate with 20% fertile DMs, although in both cases 20% of eggs intended to be daughters become daughters. One explanation is that, with the reject DMs scenario, demographic stochasticity in rejection success is greater when population size is small. Another explanation is genetic stochasticity; the chance to include a wider variety of CSD alleles is higher when each female produces some daughters. In the simulations, allelic diversity is slightly but consistently higher in the partially fertile DMs scenario at low dispersal rate than in the reject DMs scenario (Figure 5). Female parasitoids may bias toward producing more daughters when inbreeding occurs in the population (Antolin et al., 2003; Cook & Crozier, 1995; Godfray, 1994). In our simulations, this strategy mitigates extinction risk only little. Because we assume all DMs are sterile in the daughter‐biased sex ratio scenario, females that mated with DMs produced even fewer sons (and no daughters). If DMs are partially fertile, daughter‐biased sex ratio could be expected to increase parasitoid persistence more substantially. Viability and fertility of DMs remain uncertain for many species (Harpur et al., 2012), but effectively sterile DMs could be more common than inviable DMs because deleterious recessive alleles are purged in haploid males in haplodiploid taxa (Zayed, 2004). Limited evidence indicates that, in most of parasitoid species with sl‐CSD, DMs are as viable as females, and a small fraction has partially viable or inviable DMs (Harper et al., 2016; Harpur et al., 2012). Partially fertile DMs have been found in several species, while some appear effectively sterile (de Boer et al., 2007; Elias et al., 2009, 2010; Harpur et al., 2012; Zaviezo et al., 2017). Only one species has been confirmed to have fully fertile DMs (Cowan & Stahlhut, 2004).

Our results agree with suggestions made by previous studies that being dispersive is a good strategy to maintain genetic diversity at the CSD locus and alleviate extinction risk (Antolin & Strand, 1992; Collet et al., 2020; Cook & Crozier, 1995; Faria et al., 2016; Ruf et al., 2011; van Wilgenburg et al., 2006). Solitary parasitoids are generally good fliers (Godfray, 1994; van Wilgenburg et al., 2006). At or near the top of the food chain, parasitoids often experience sparser resources in space to reach than their hosts do (Holt, 2002; Martinson & Fagan, 2014; van Nouhuys, 2005). Moreover, Inbreeding avoidance is one of the factors that can promote the evolution of dispersal (Saastamoinen et al.,  2018). Field studies have shown that parasitoids can disperse for 10s of meters up to 7.5 km (Collet et al., 2020; Couchoux et al., 2016; Nair et al., 2016; Roland et al., 2000) and can be wind‐borne over several kilometers (Kristensen, Schellhorn, Hulthen, Howie, & De Barro, 2013). Wing polymorphism and sex‐biased dispersal have been documented in multiple species of parasitoids (Asplen et al., 2009; Collet et al., 2020; Godfray, 1994; Ruf et al., 2011), suggesting that dispersal is adaptive. Some species have a premating refractory period during which one or both sexes of newly emerged adults disperse before mating (Godfray, 1994; van Wilgenburg et al., 2006). Collet et al. (2020) observed sib‐mating tolerance in natural populations of a parasitoid that possesses sl‐CSD and produces sterile DMs and suggested that dispersal probably reduces production of DMs effectively enough for the parasitoid not to evolve other behavioral or genetic countermeasures.

### 4.4 Fluctuating host populations

Large fluctuations, when spatially and temporally autocorrelated at the levels we introduce into the model, further endanger population persistence of parasitoids in our simulations. The variability of fluctuations is based on a previous study, which used long‐term empirical data that showed increasing frequencies of extreme butterfly host abundances in the last decade (Nair et al., 2018; Tack et al., 2015). Because low abundance of hosts can cause population bottlenecks in parasitoids, we expect that positive spatial or temporal autocorrelation in host abundance would exacerbate bottleneck effects. In a butterfly metapopulation studied by Jangjoo et al. (2020), a prolonged bottleneck event depleted genetic diversity from local populations and disrupted the spatial pattern of population differentiation more than a bottleneck of a shorter duration. In recent years, weather patterns may have become more spatially autocorrelated in some areas (Kahilainen et al., 2018). In our models, spatially autocorrelated fluctuations do not reduce persistence as much as temporally autocorrelated noise probably because the parasitoid disperses at moderate to high rates in a large fraction of the simulations (Figure S2). Dispersal is one of the mechanisms that can cause spatial autocorrelation in population dynamics (Liebhold et al., 2004), so that externally imposing spatial autocorrelation may not further increase the risk of population bottlenecks in our simulations. There is no reason to assume that fluctuations in weather conditions themselves are temporally autocorrelated. However, parasitoids experience fluctuations that have been filtered by their hosts, and environmental variation could be modified to become more alike to red noise as it propagates through the food chain (Kuparinen et al., 2018; Ripa et al., 1998; Sugihara, 1995). Although the CSD system appears to be effective in mitigating the occurrence of a diploid male vortex, it could break down if fluctuations in host abundance become more extreme and temporally autocorrelated.

## CONFLICT OF INTEREST

The authors are not aware of any competing interests.

## AUTHOR CONTRIBUTION

**Etsuko Nonaka:** Conceptualization (equal); Data curation (lead); Formal analysis (lead); Investigation (lead); Methodology (equal); Writing‐original draft (lead); Writing‐review & editing (lead). **Veijo Kaitala:** Conceptualization (equal); Formal analysis (supporting); Investigation (supporting); Methodology (equal).

## Supporting information

Supplementary Material

## Data Availability

The Matlab code that generated the data used to support the findings of this study is openly available in Dryad (https://doi.org/10.5061/dryad.fbg79cnsg).
