# Supplementary material for: The effects of functional response and host abundance fluctuations on genetic rescue in parasitoids with single‐locus sex determination
Source: Ecol Evol. 2020 Oct 14;10(23):13030–43. doi: 10.1002/ece3.6889 (PMC7713968; doi:10.1002/ece3.6889)

Supplementary Information

Figure S1. The number of hosts attacked as a function of host density at six female parasitoid densities (F = 1, 5, 50, 100, 300, and 1000) for the six functional responses. F is the number of female parasitoids in the population. For models 4, 5, and 6, T_h=0.005.

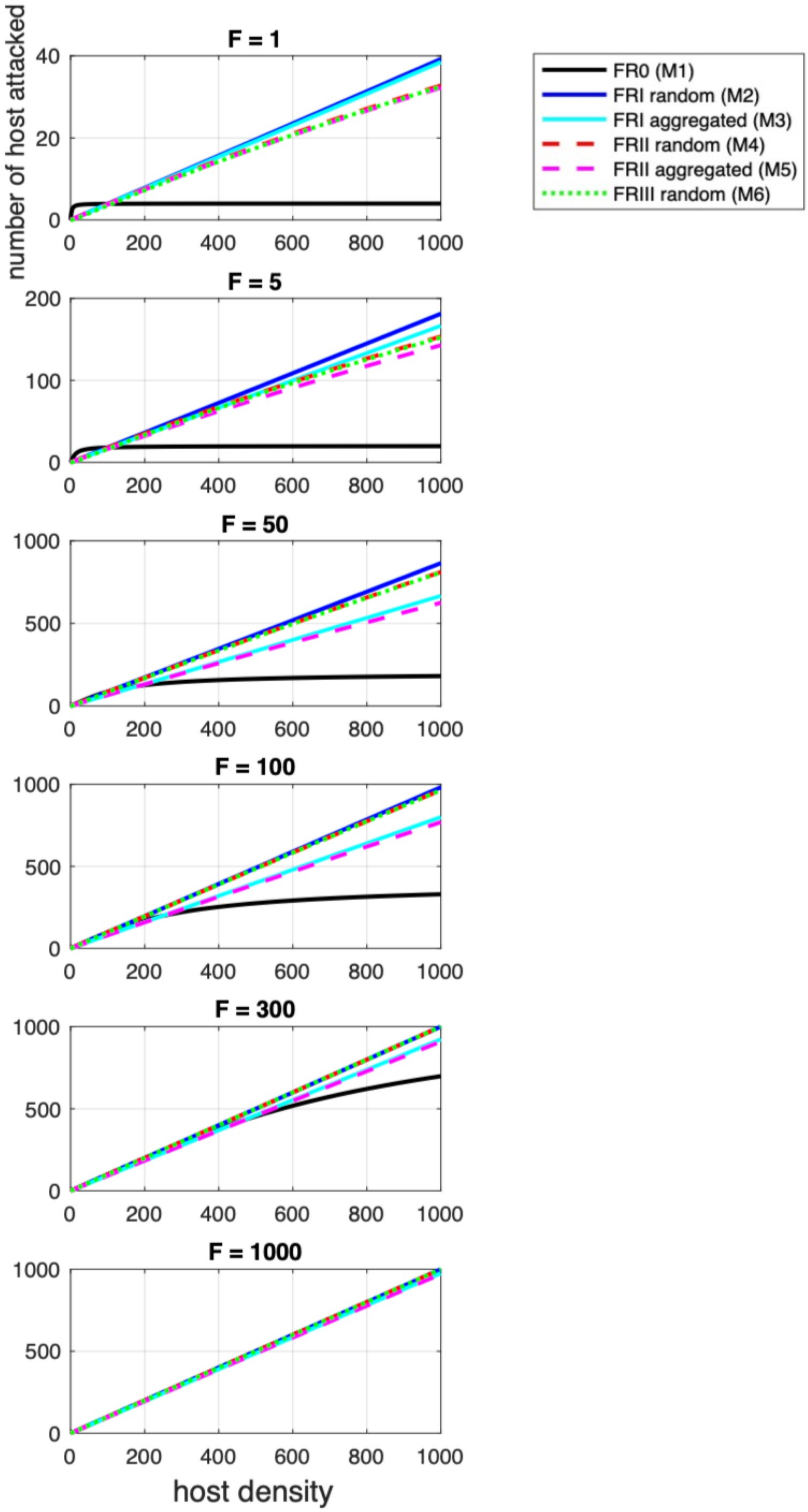

Figure S2. Population persistence. The colors indicate the number of simulations in which the parasitoid population persisted for 5000 generations. The total number of simulation is 20 for each parameter combinations indicated on the x- and y-axies. The values presented in Figure 2 in the main text is population persistence (proportion persisted) averaged over all the dipersal rates and w = [3,4,5] or a = [0.03:0.005:0.05]. In Figure 3 in the main text, the same data are shown by dispersal rate.

Model 1: no added fluctuation
Population persistence

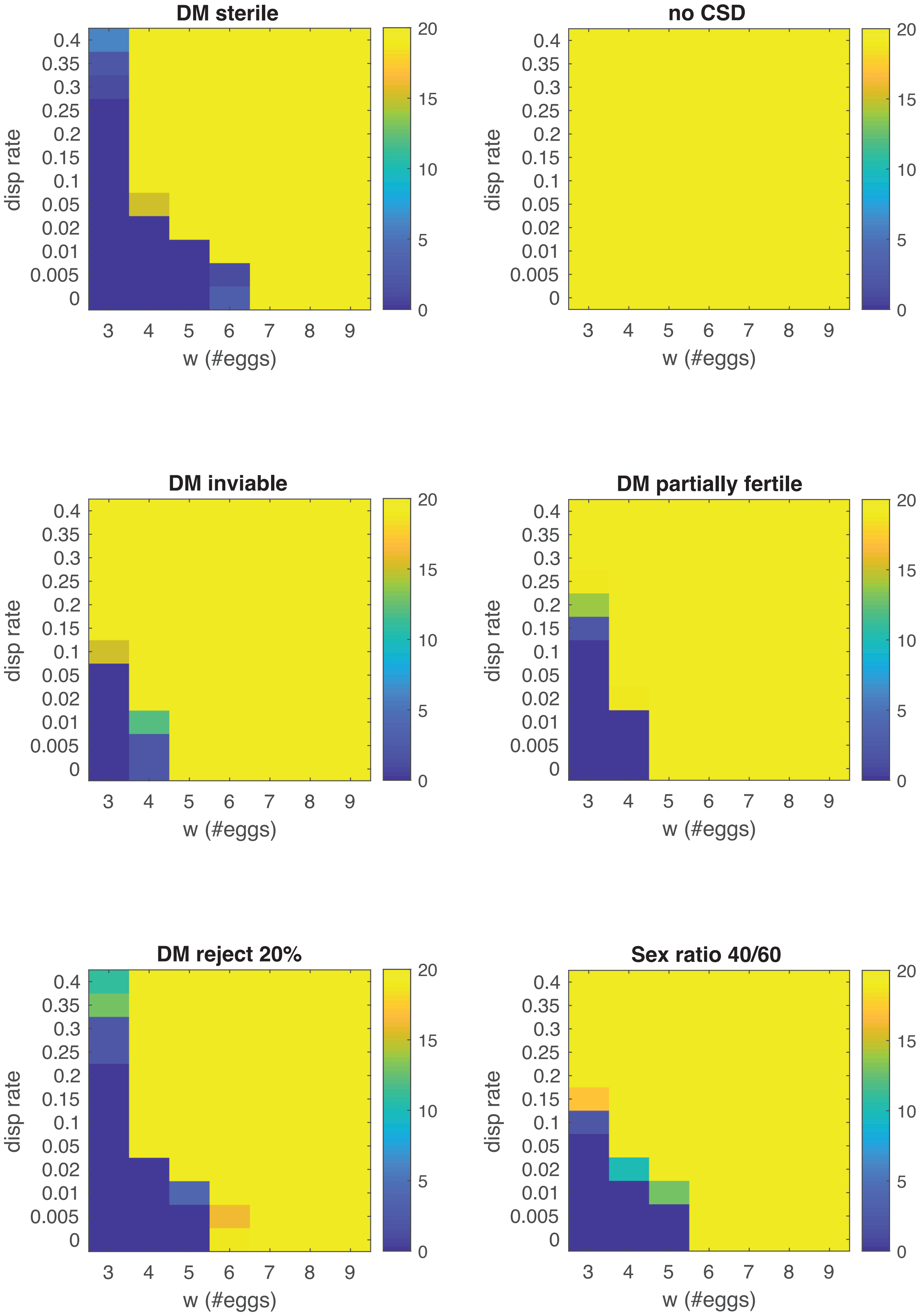

Model 1: small fluctuation
Population persistence

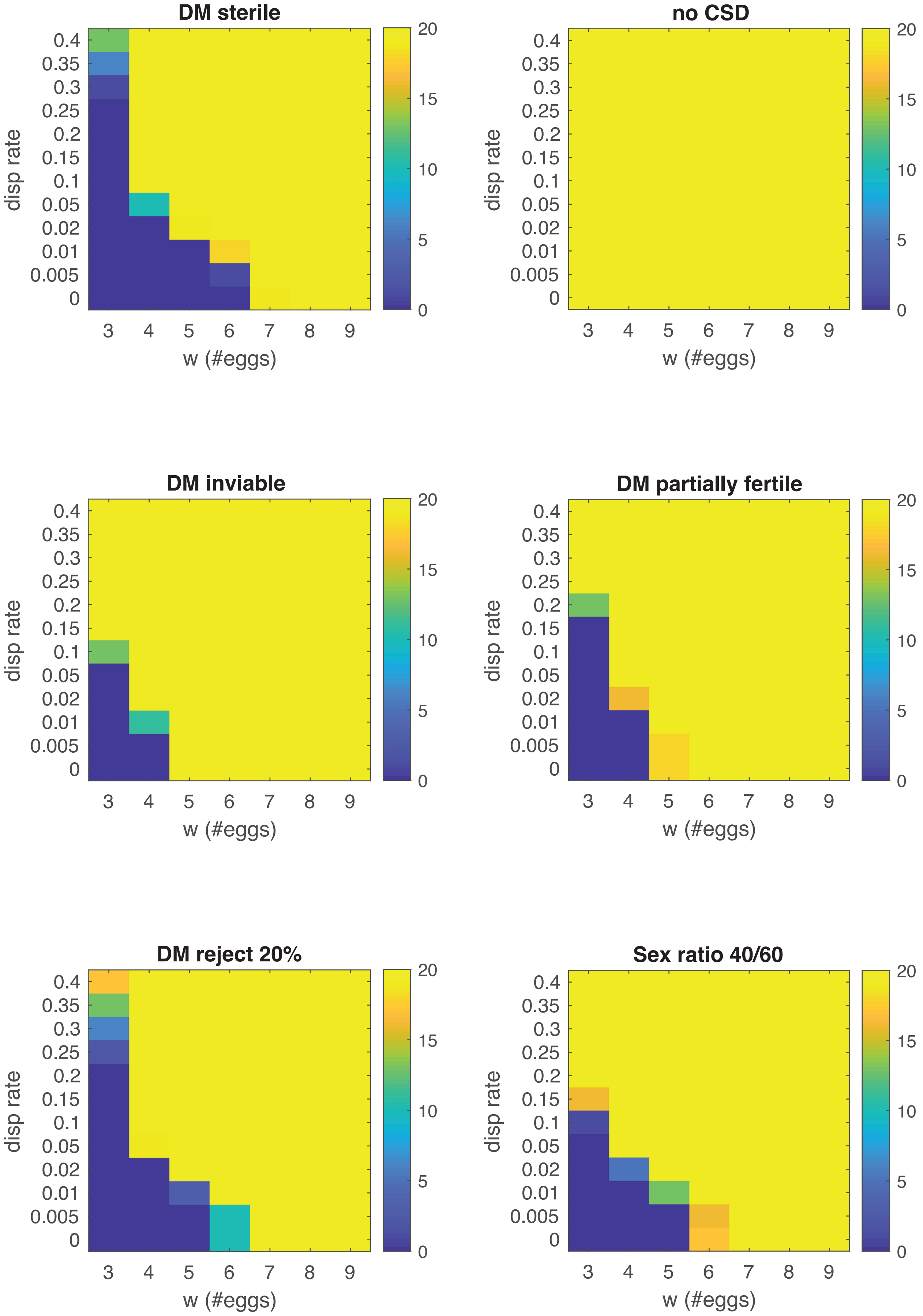

Model 1: large fluctuation
Population persistence

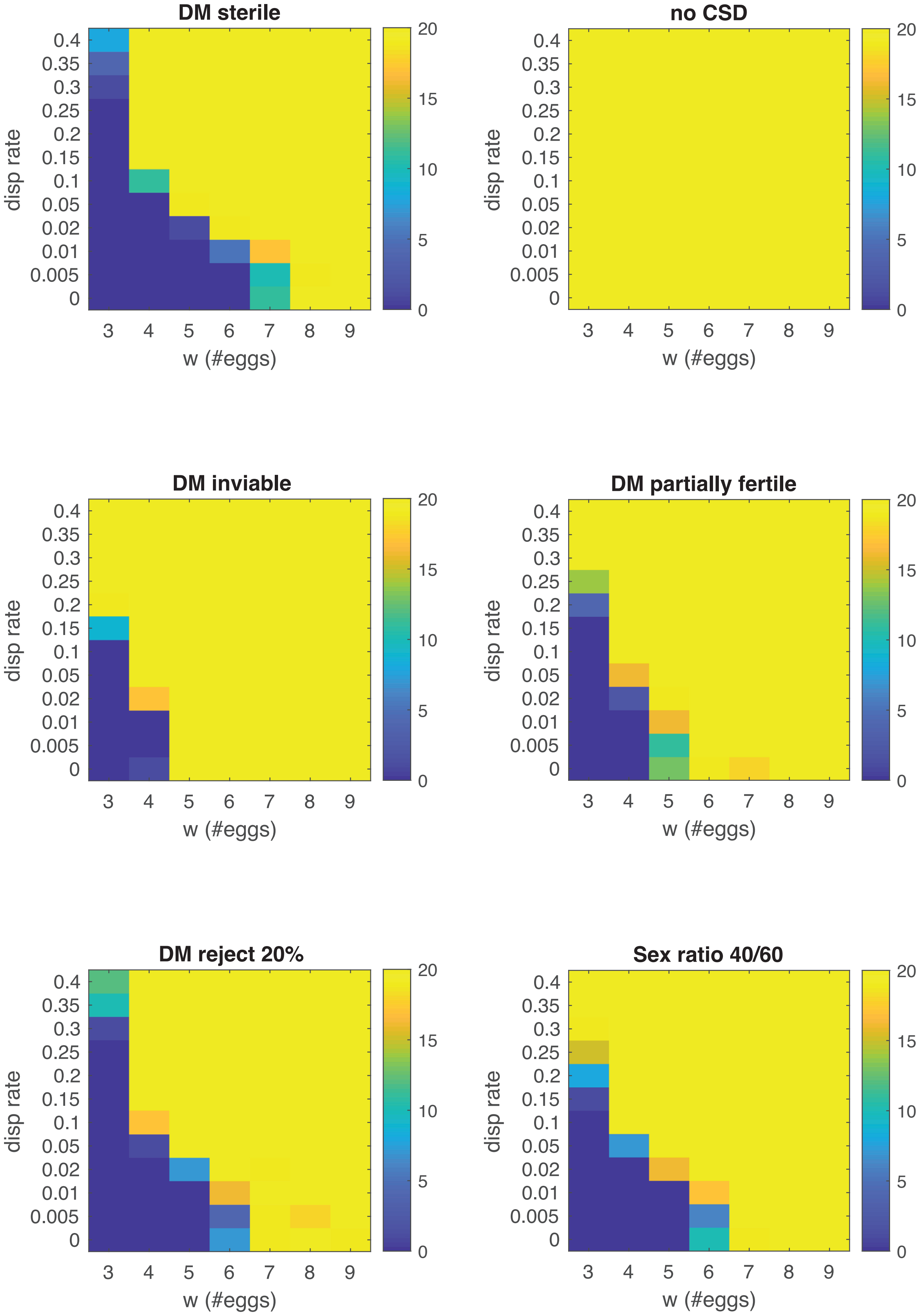

Model 1: spatially autocorrelated large fluctuation
Population persistence

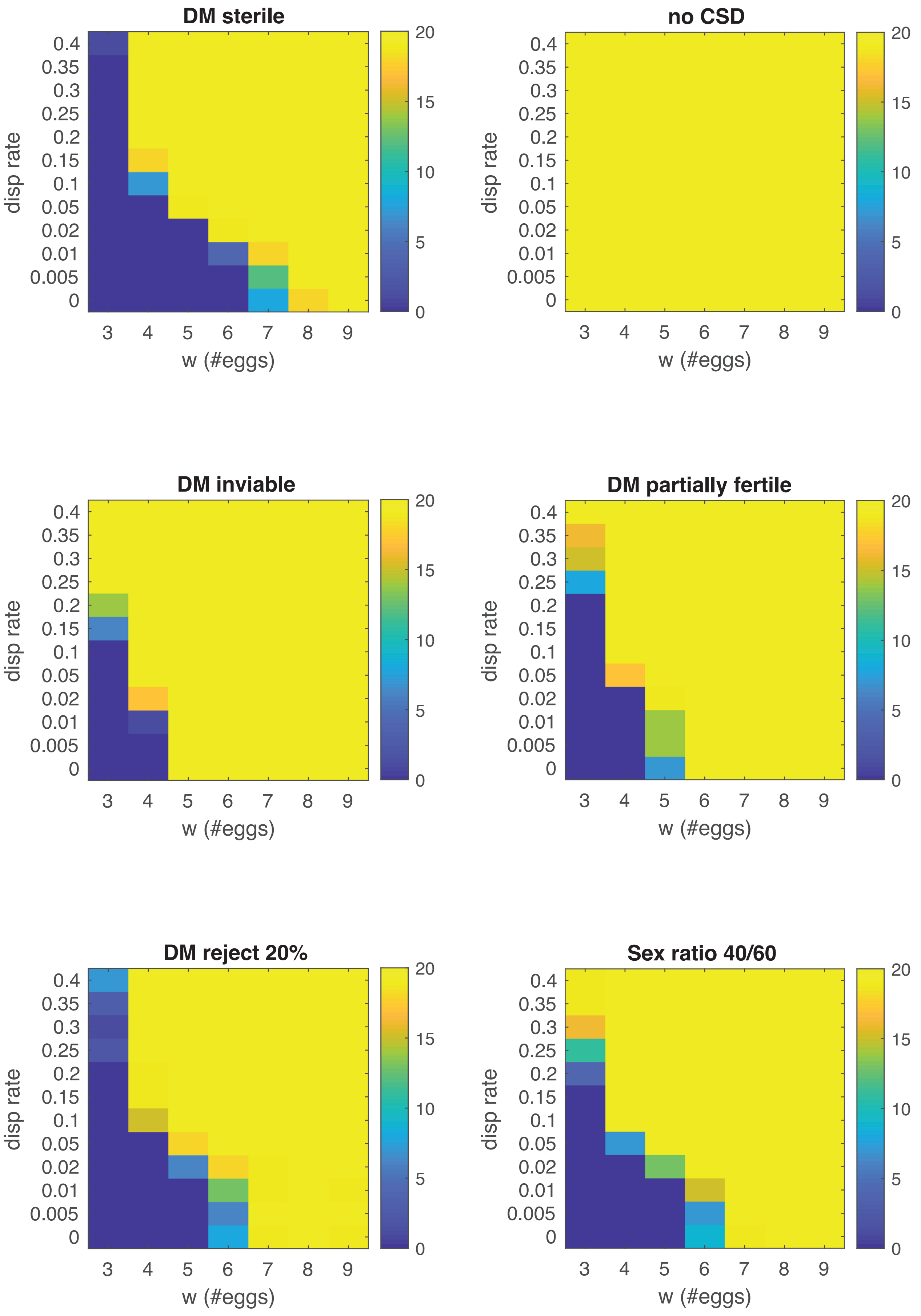

Model 1: large rednoise
Population persistence

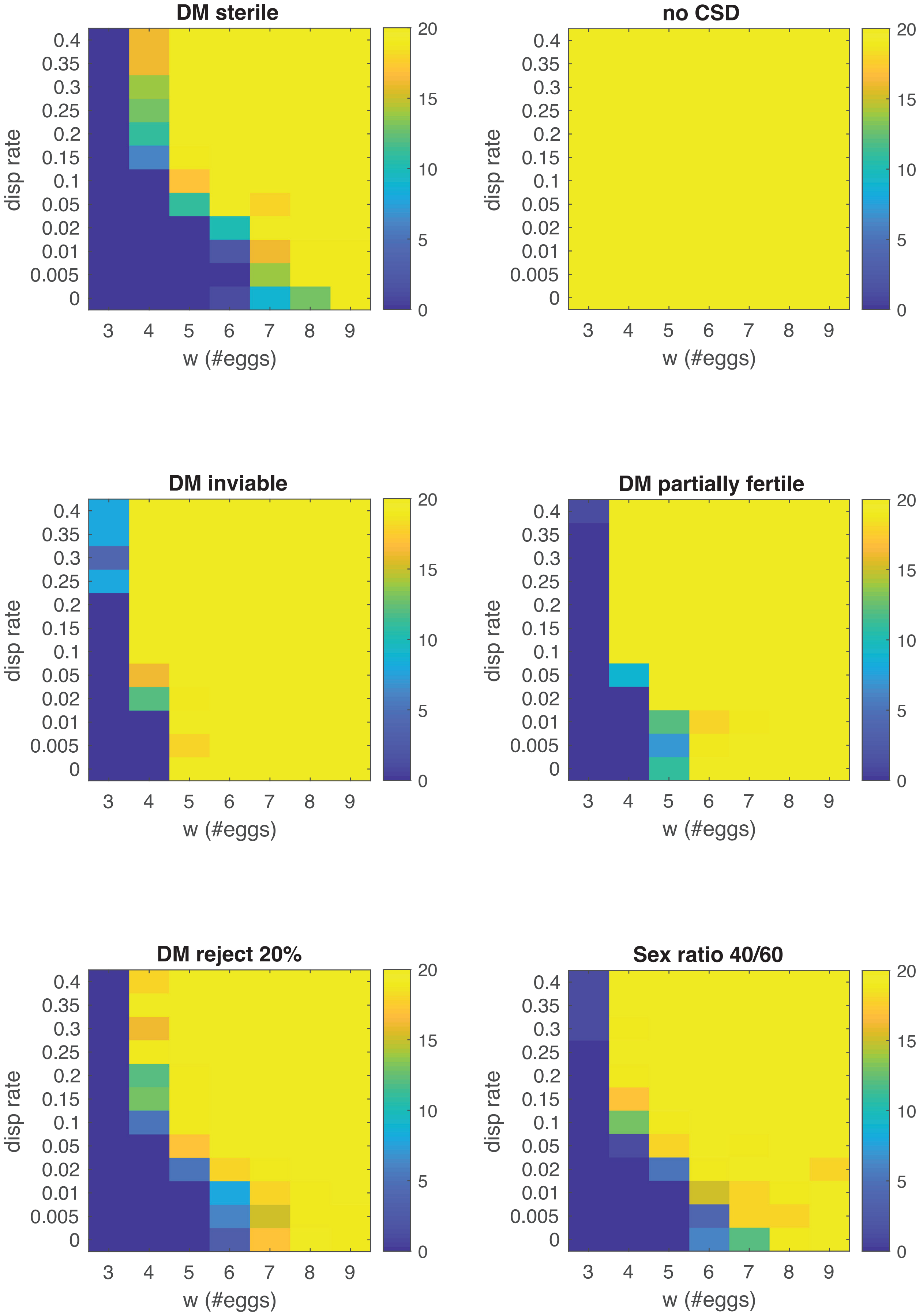

Model 2: no added fluctuation
Population persistence

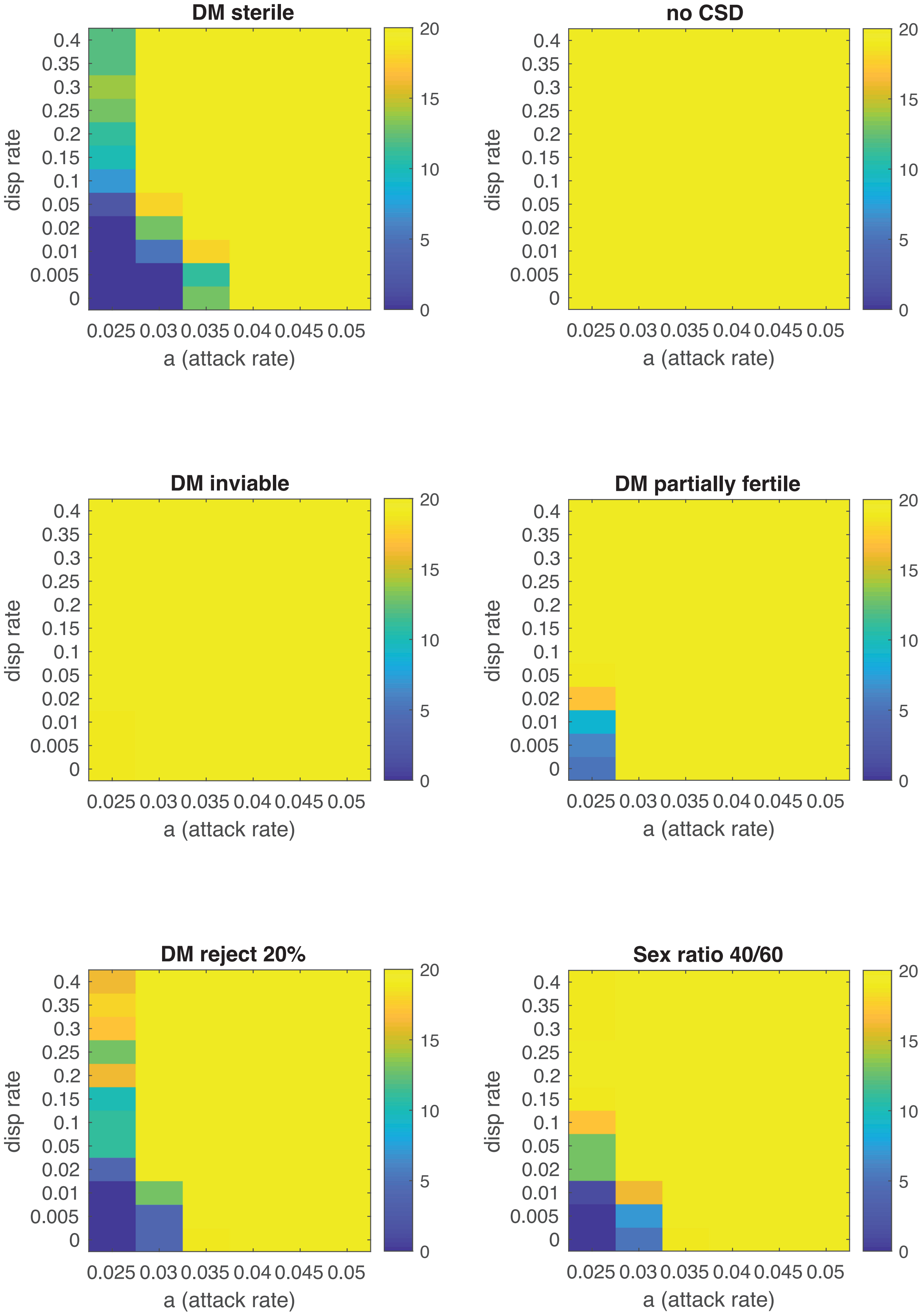

Model 2: small fluctuation
Population persistence

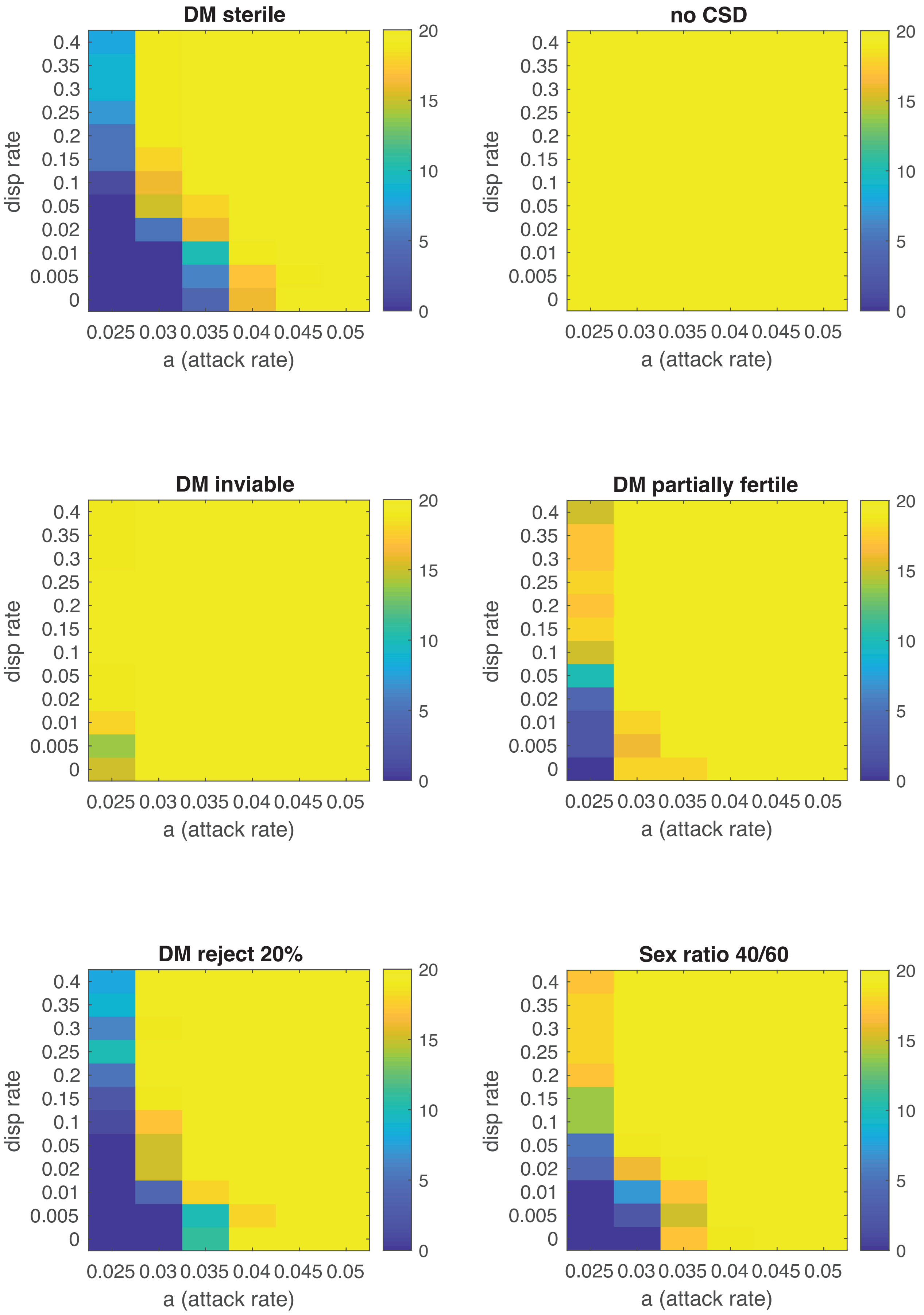

Model 2: large fluctuation
Population persistence

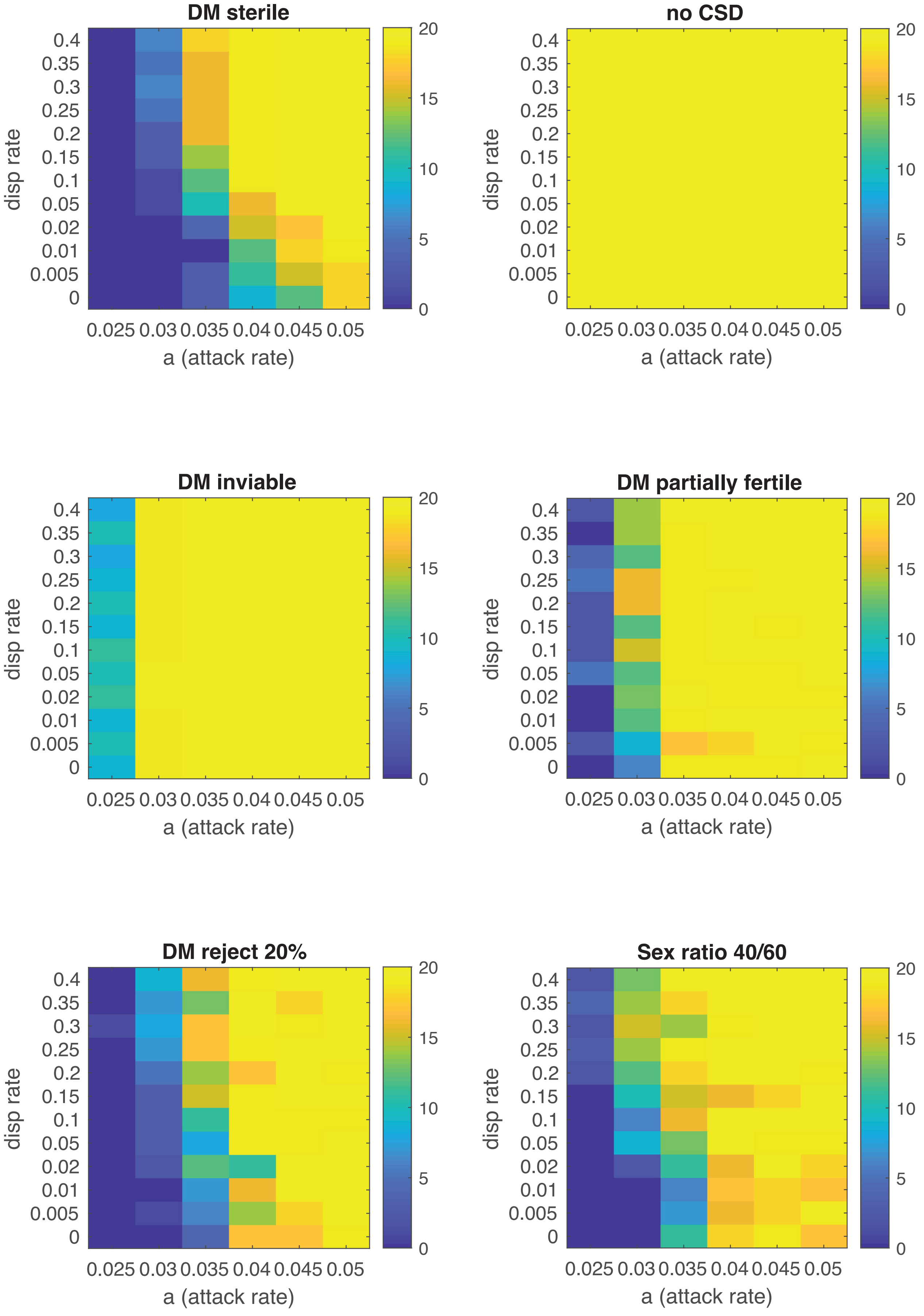

Model 2: spatially autocorrelated large fluctuation
Population persistence

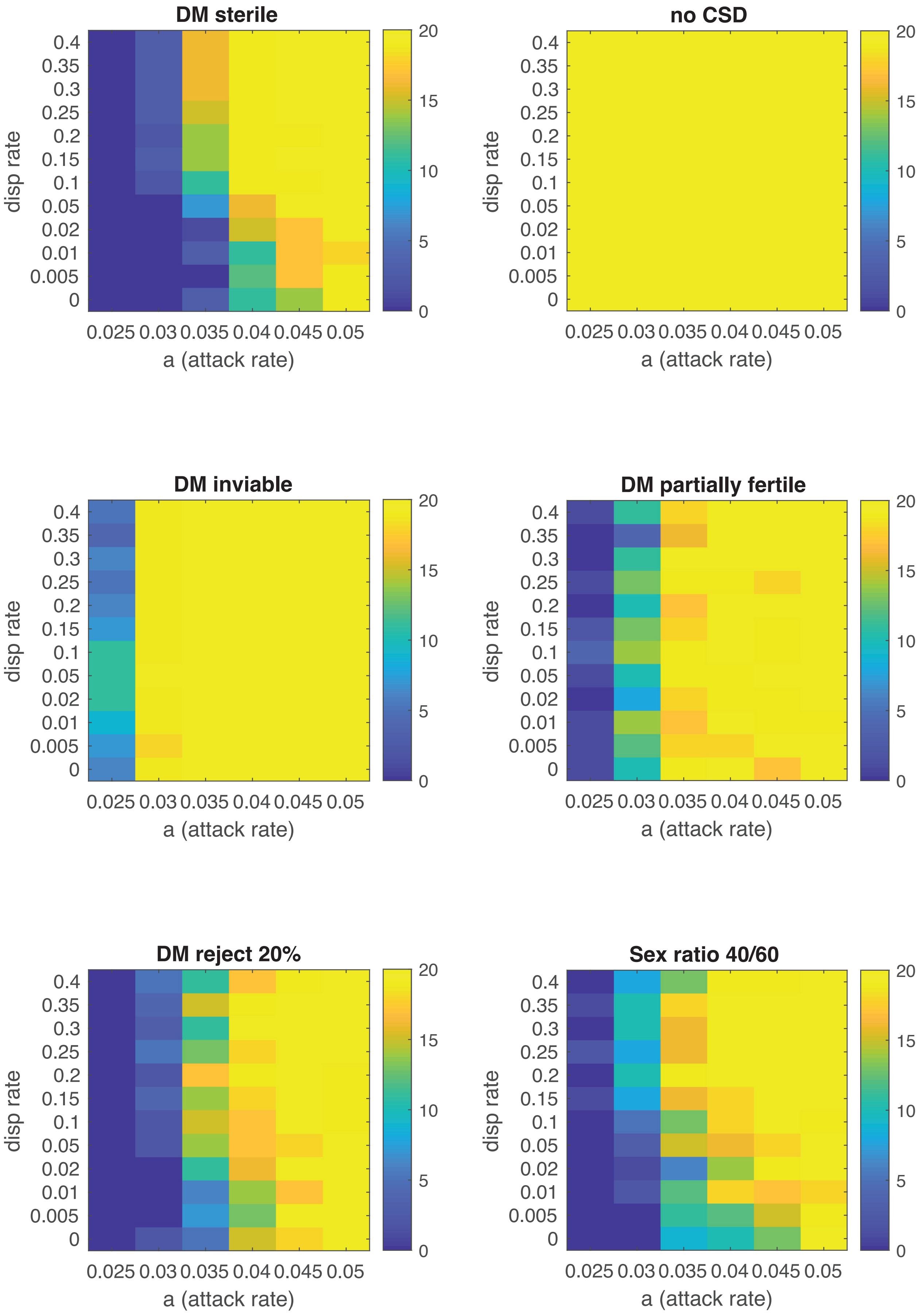

Model 2: large rednoise
Population persistence

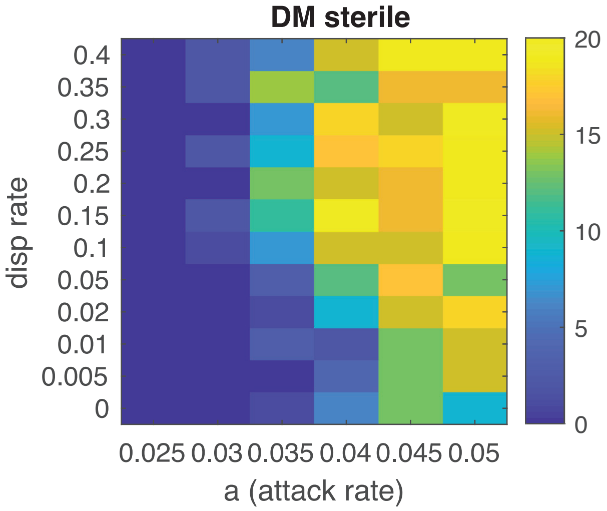

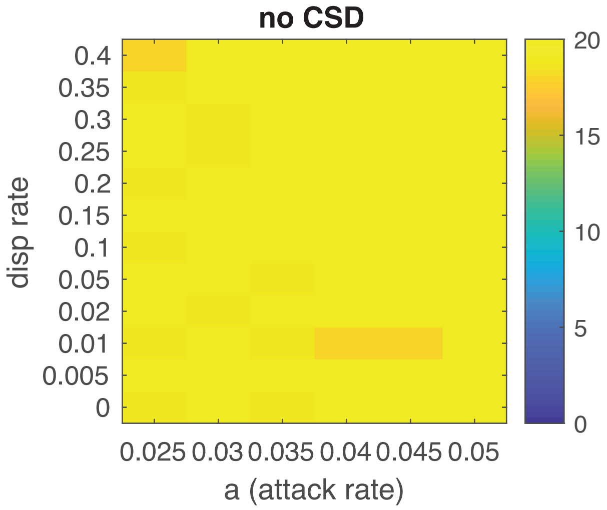

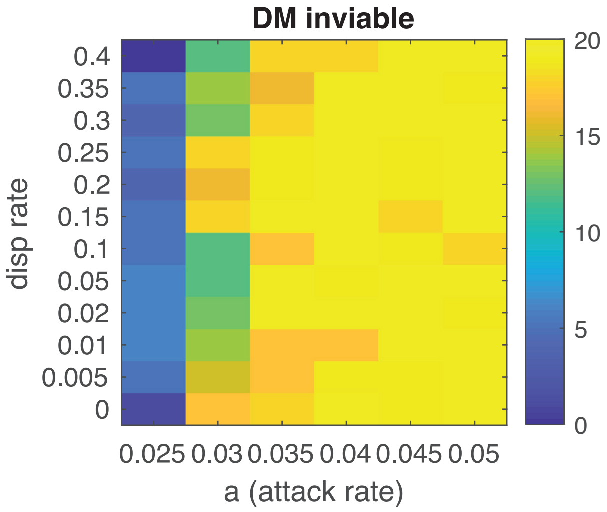

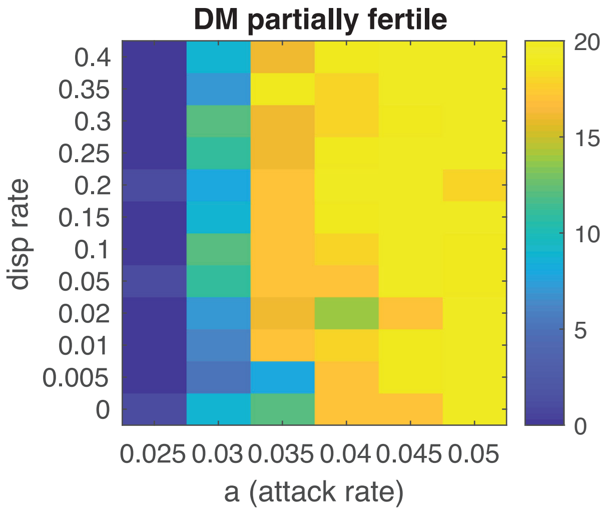

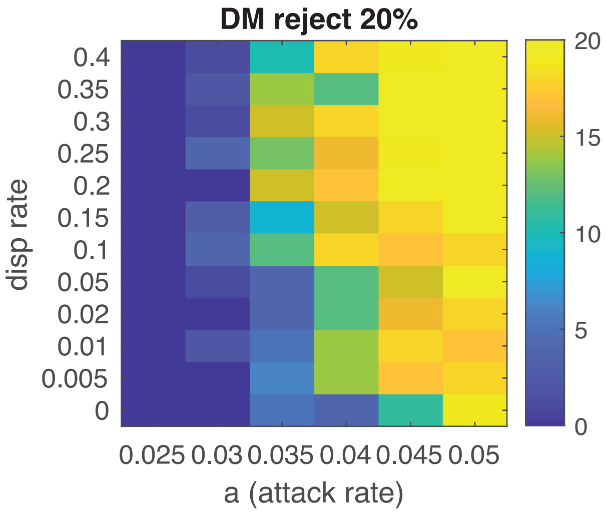

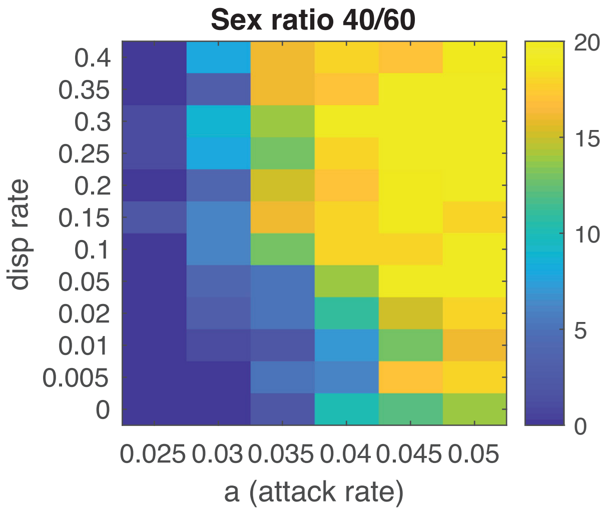

Model 3: no added fluctuation
Population persistence

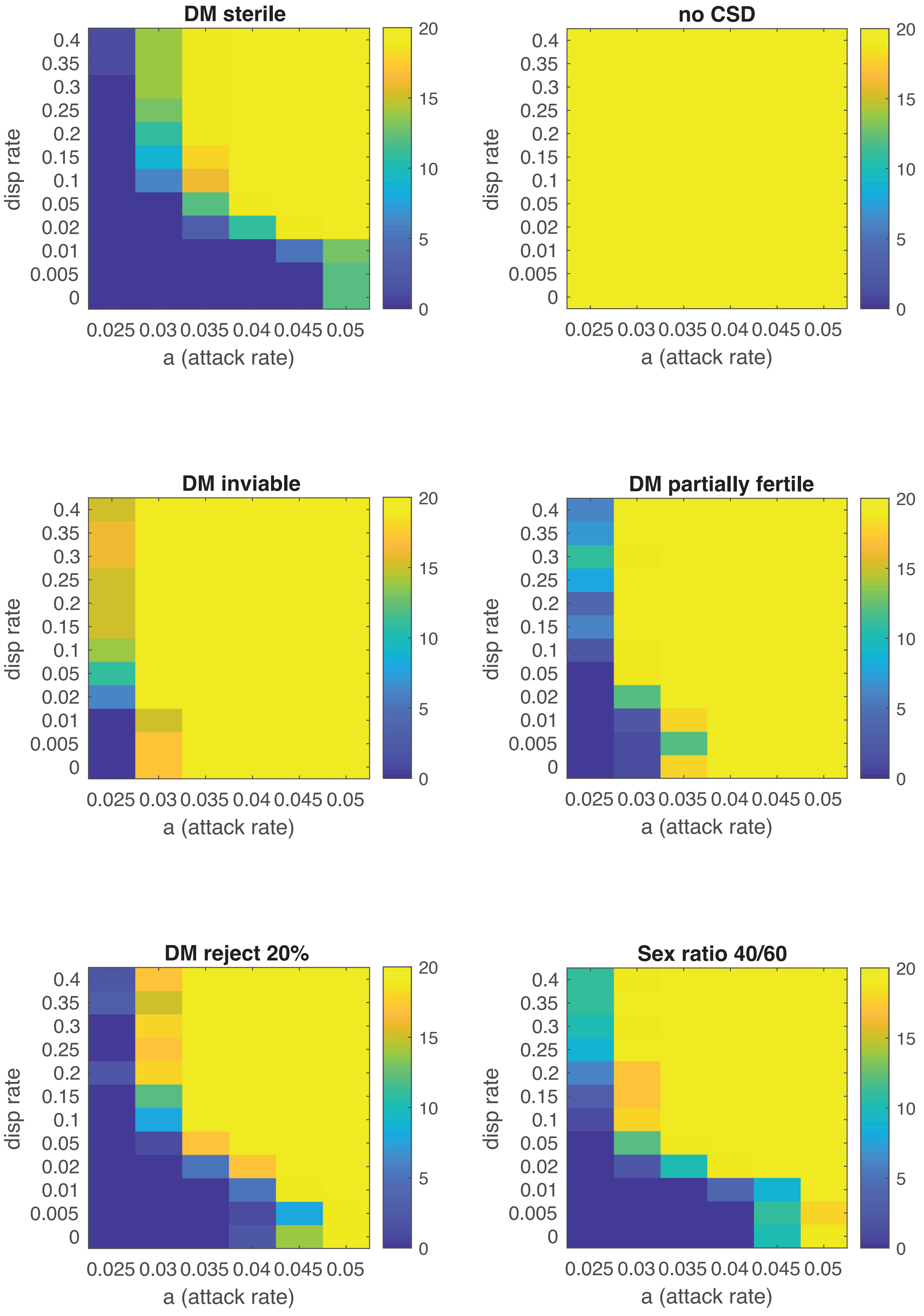

Model 3: small fluctuation
Population persistence

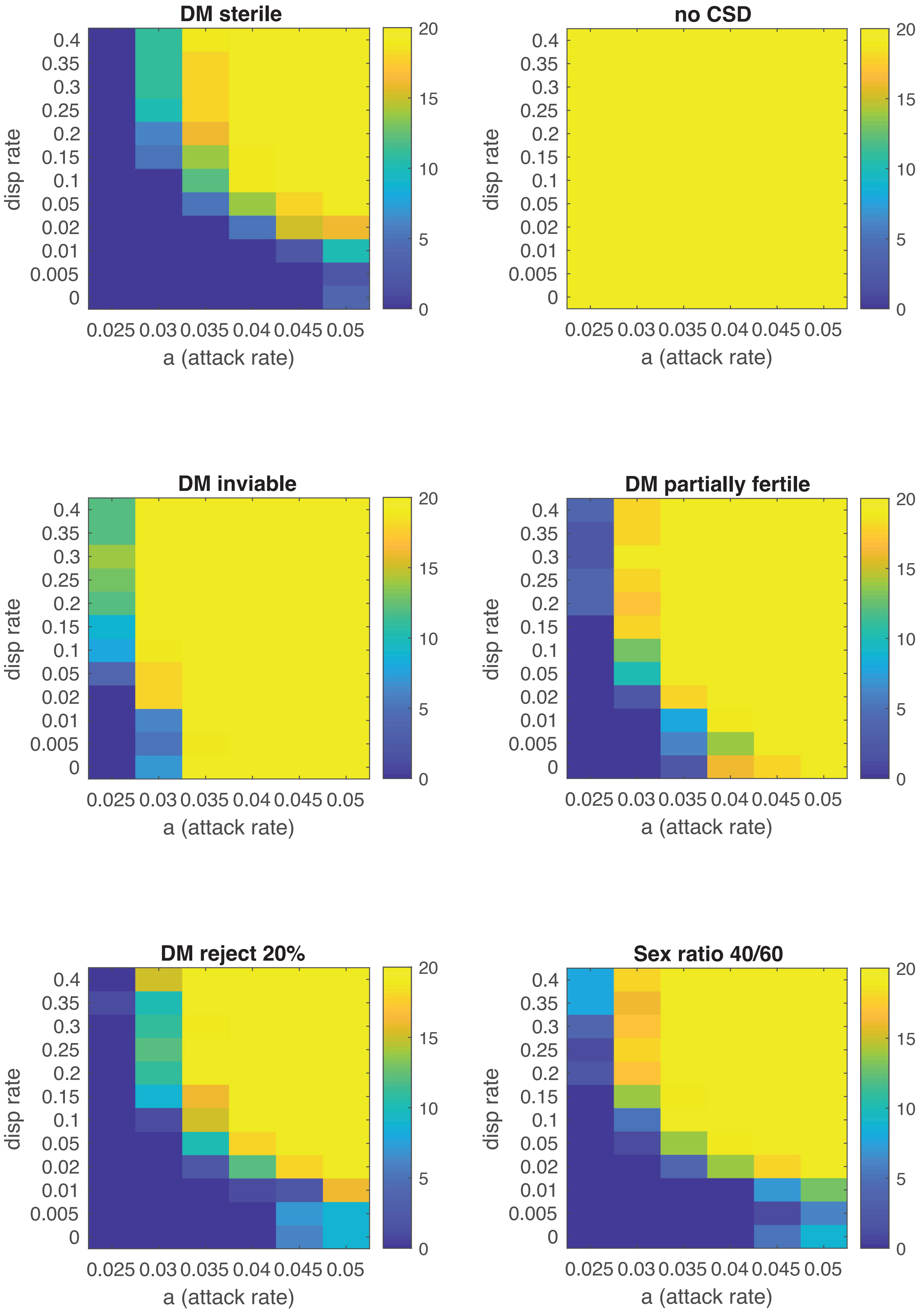

Model 3: large fluctuation
Population persistence

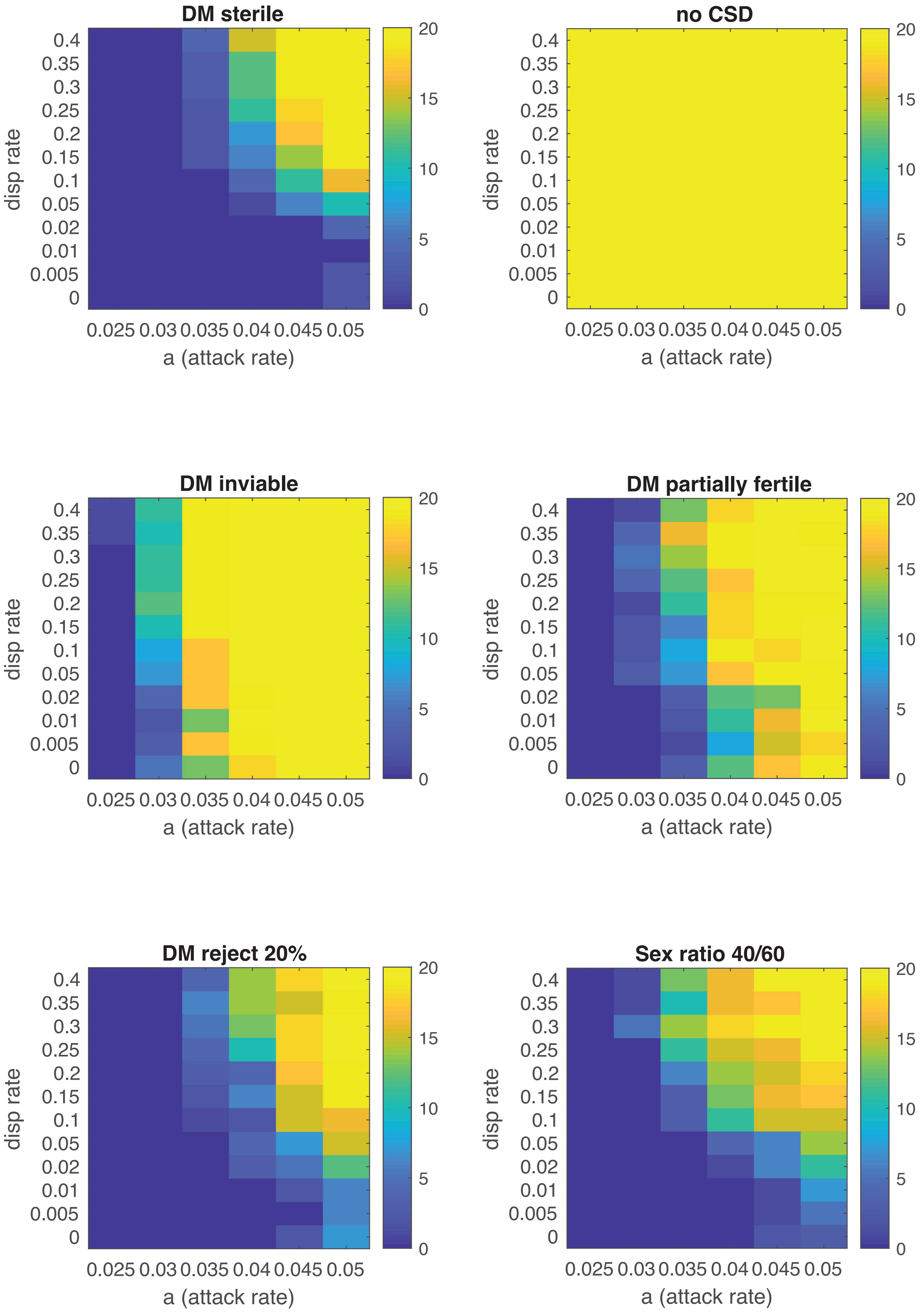

Model 3: spatially autocorrelated large fluctuation
Population persistence

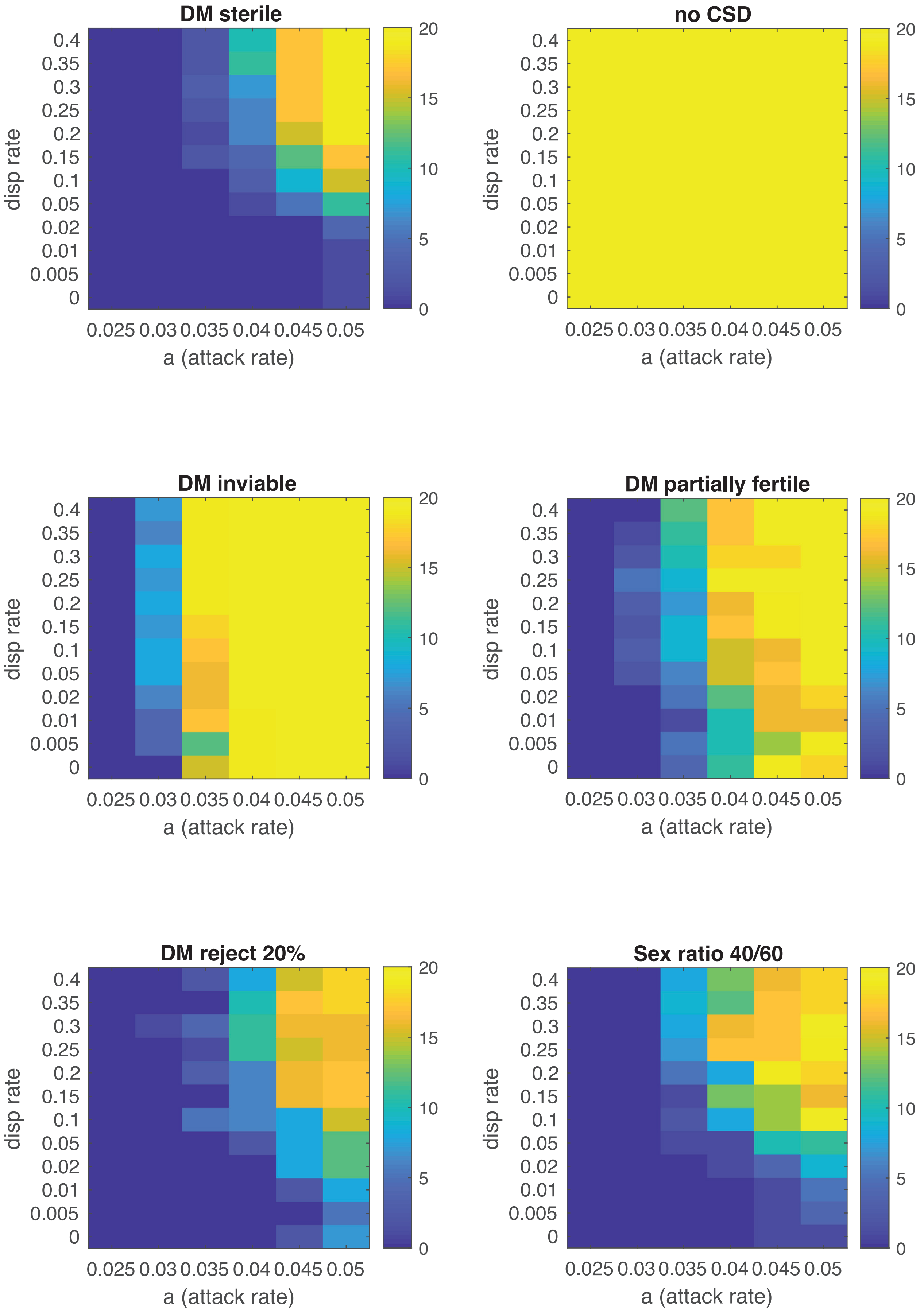

Model 3: large rednoise
Population persistence

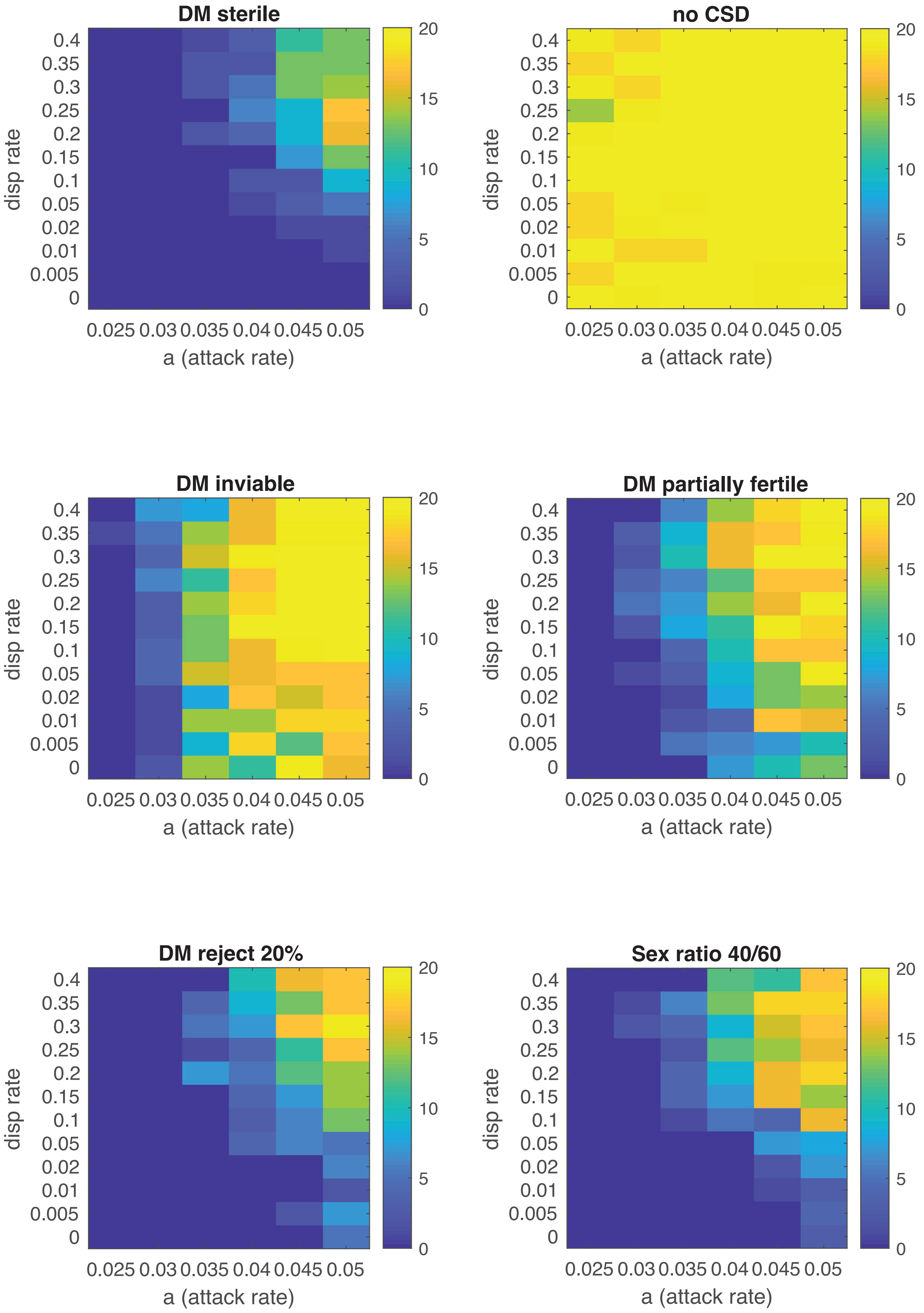

Model 4: no added fluctuation
Population persistence

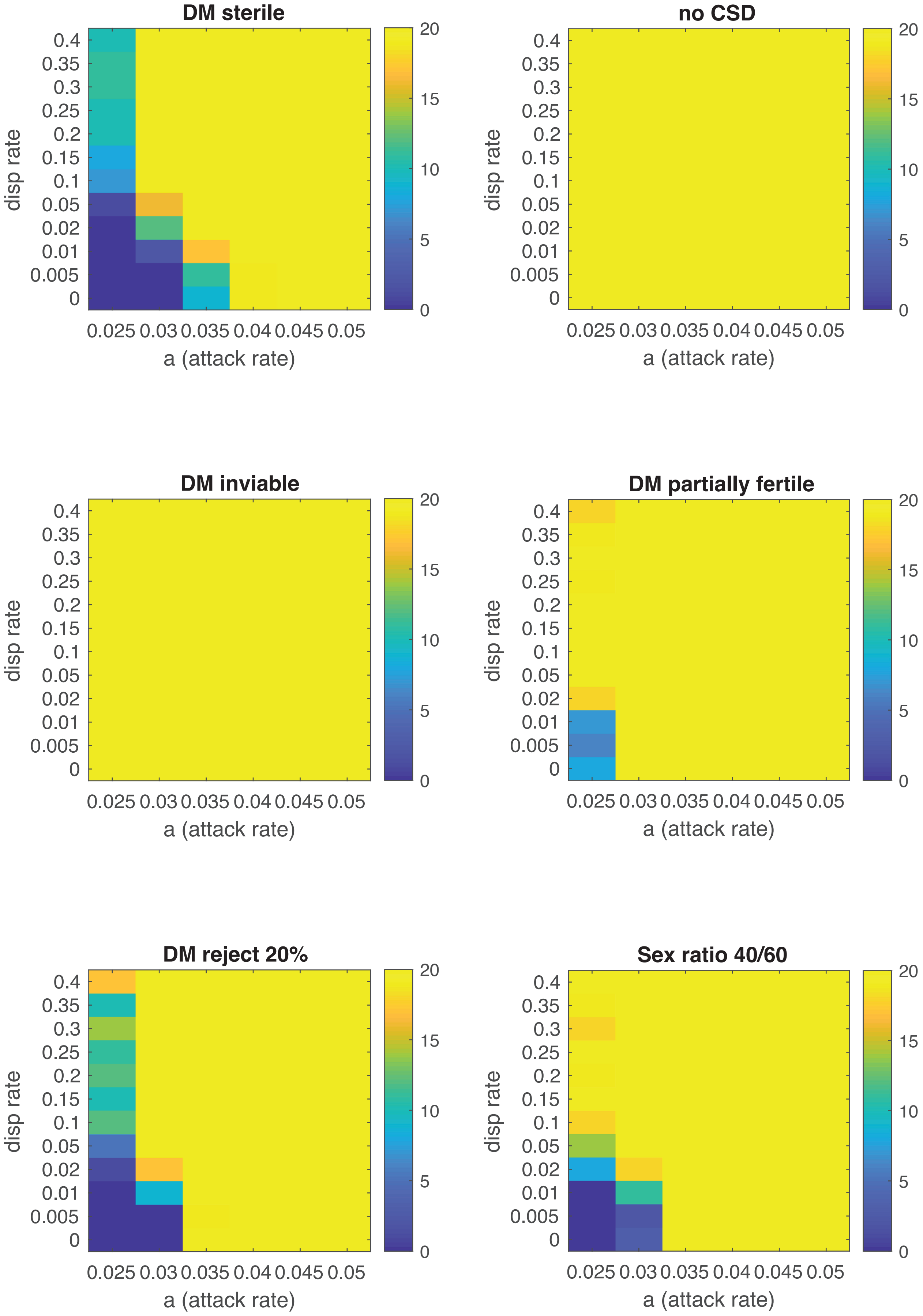

Model 4: small fluctuation
Population persistence

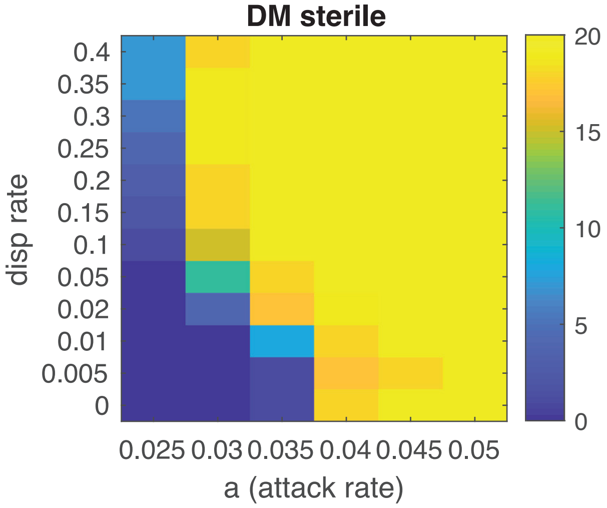

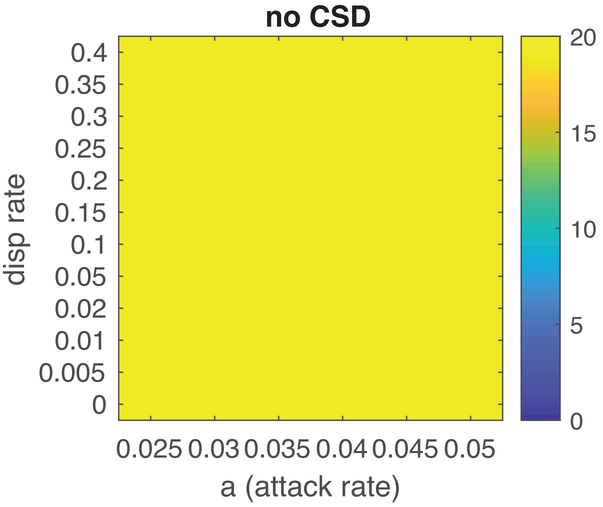

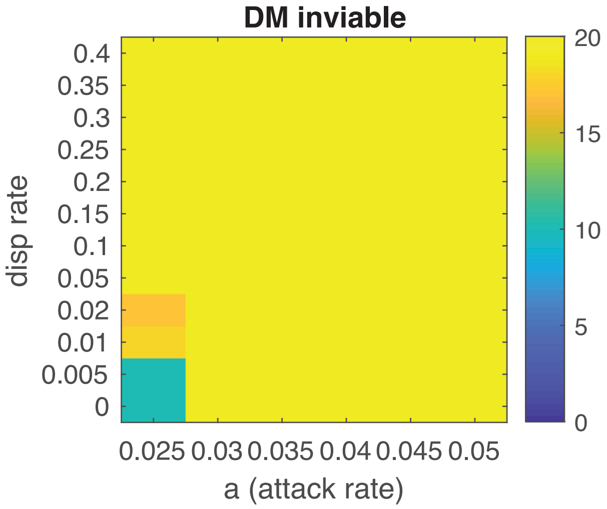

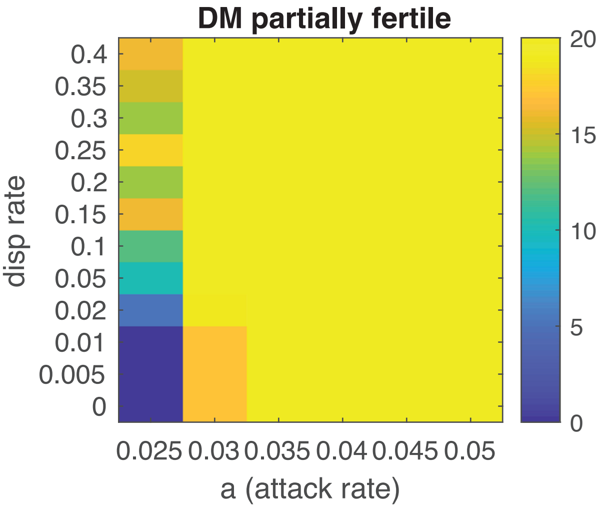

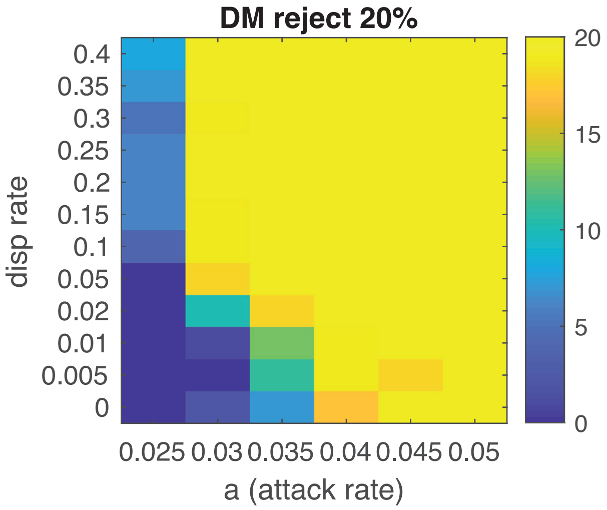

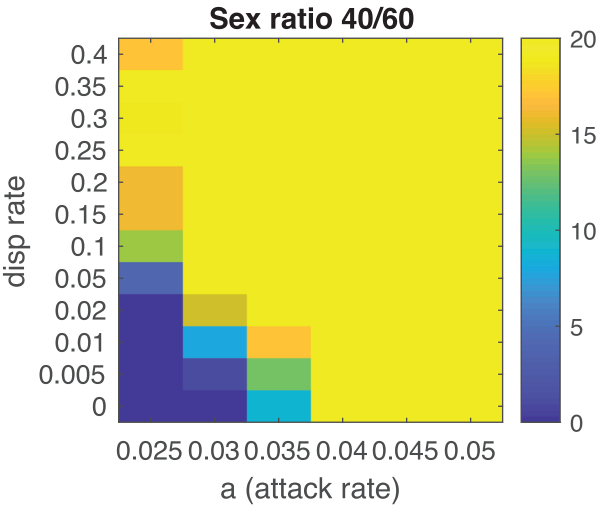

Model 4: large fluctuation
Population persistence

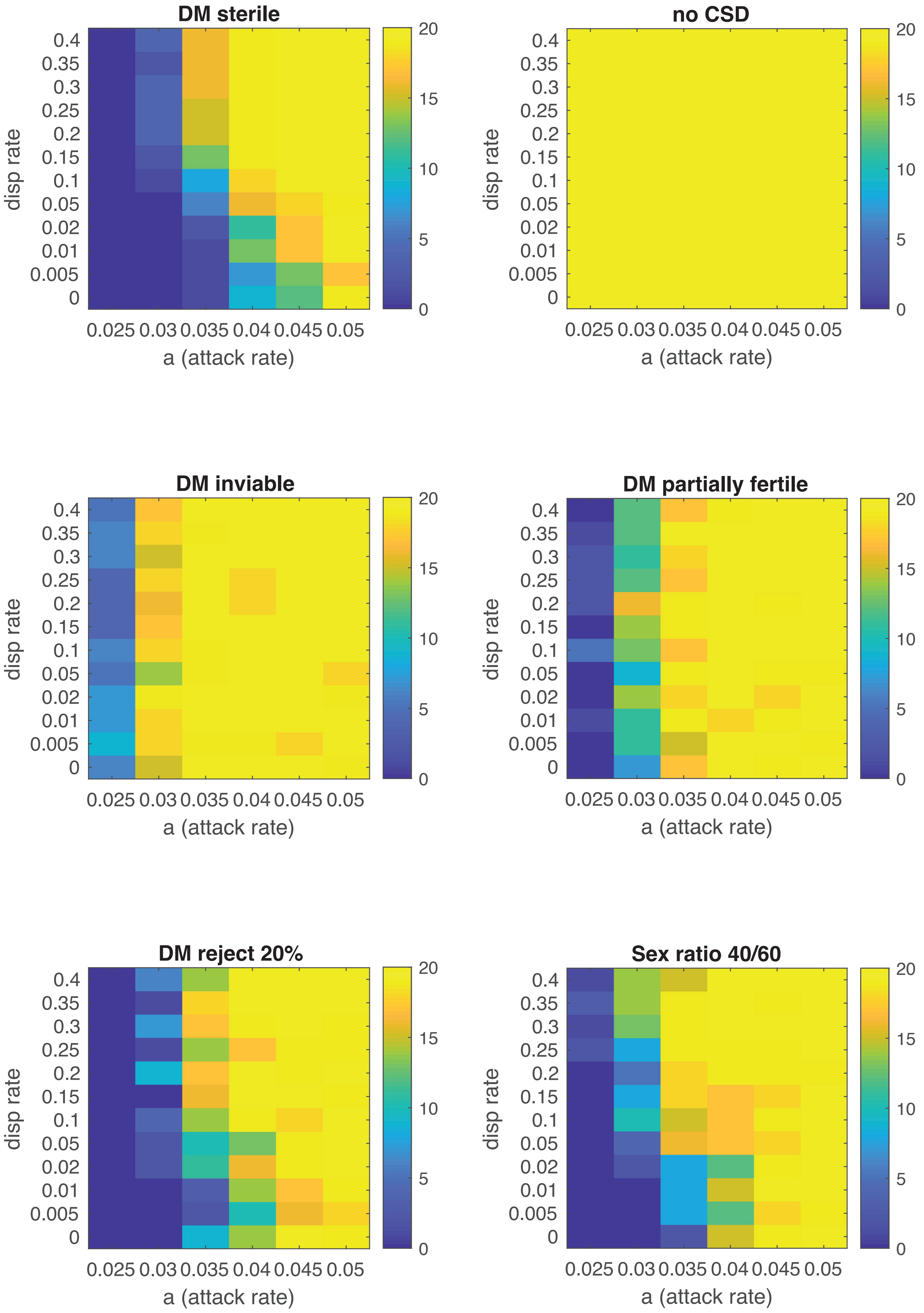

Model 4: spatially autocorrelated large fluctuation
Population persistence

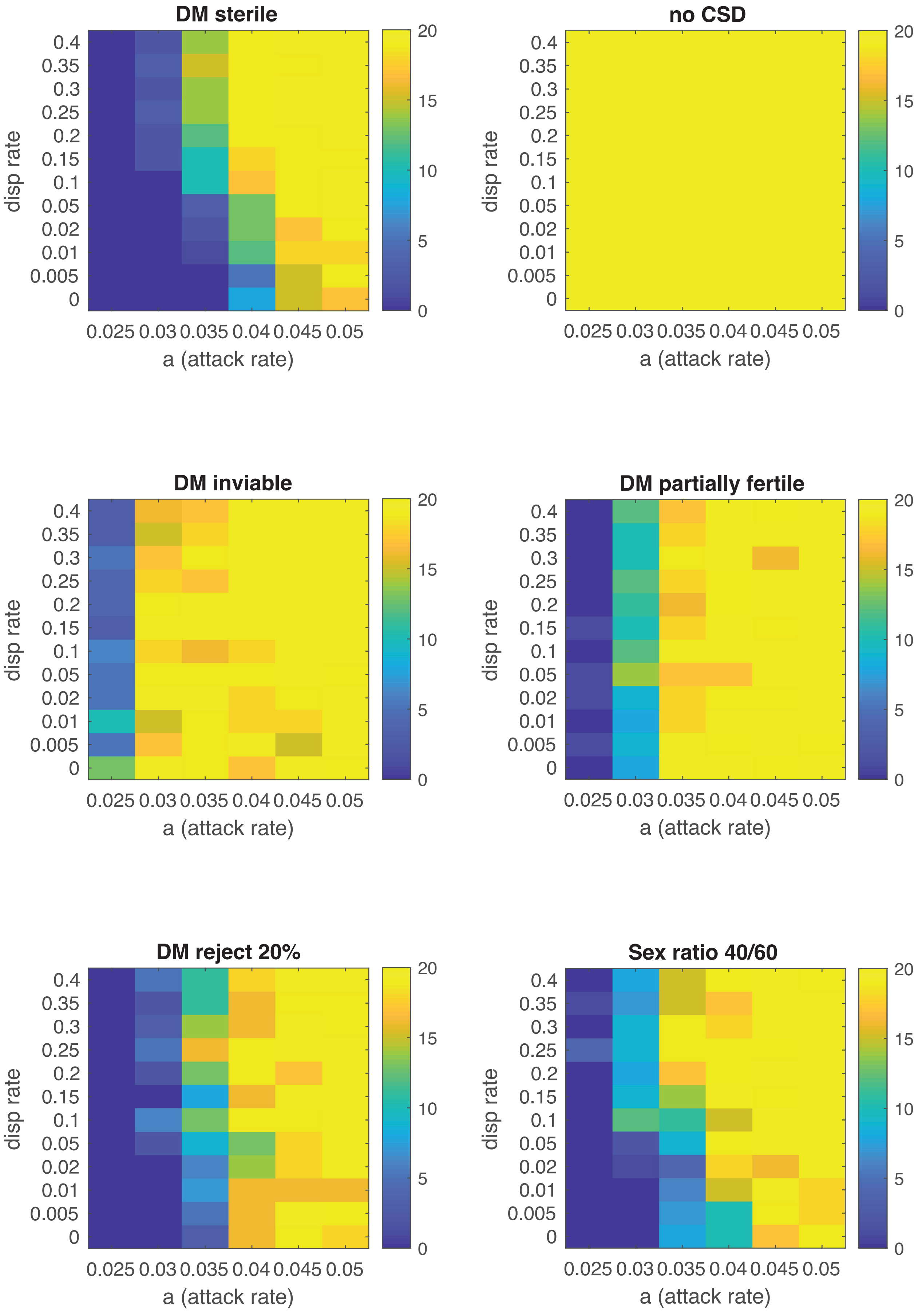

Model 4: large rednoise
Population persistence

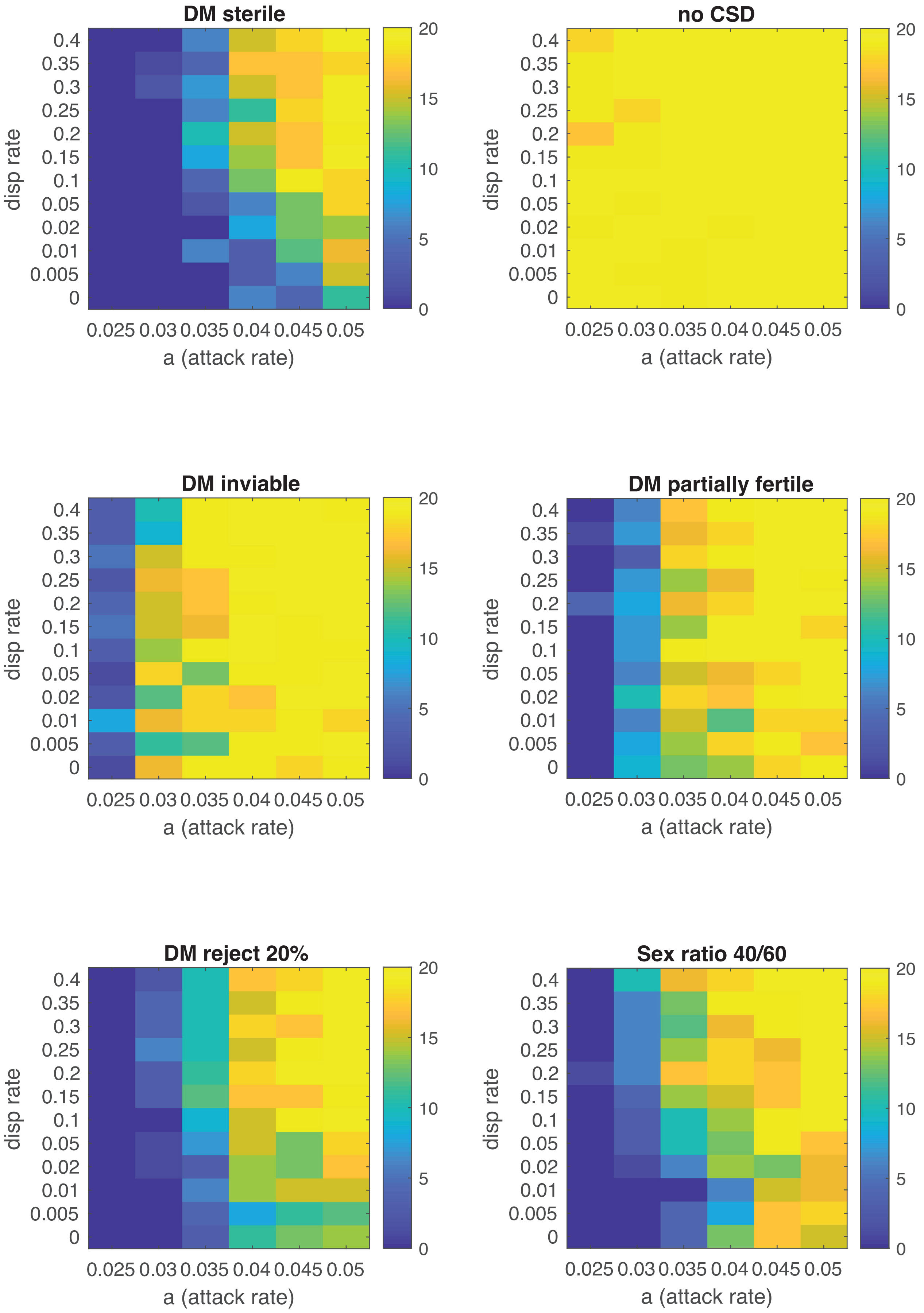

Model 5: no added fluctuation
Population persistence

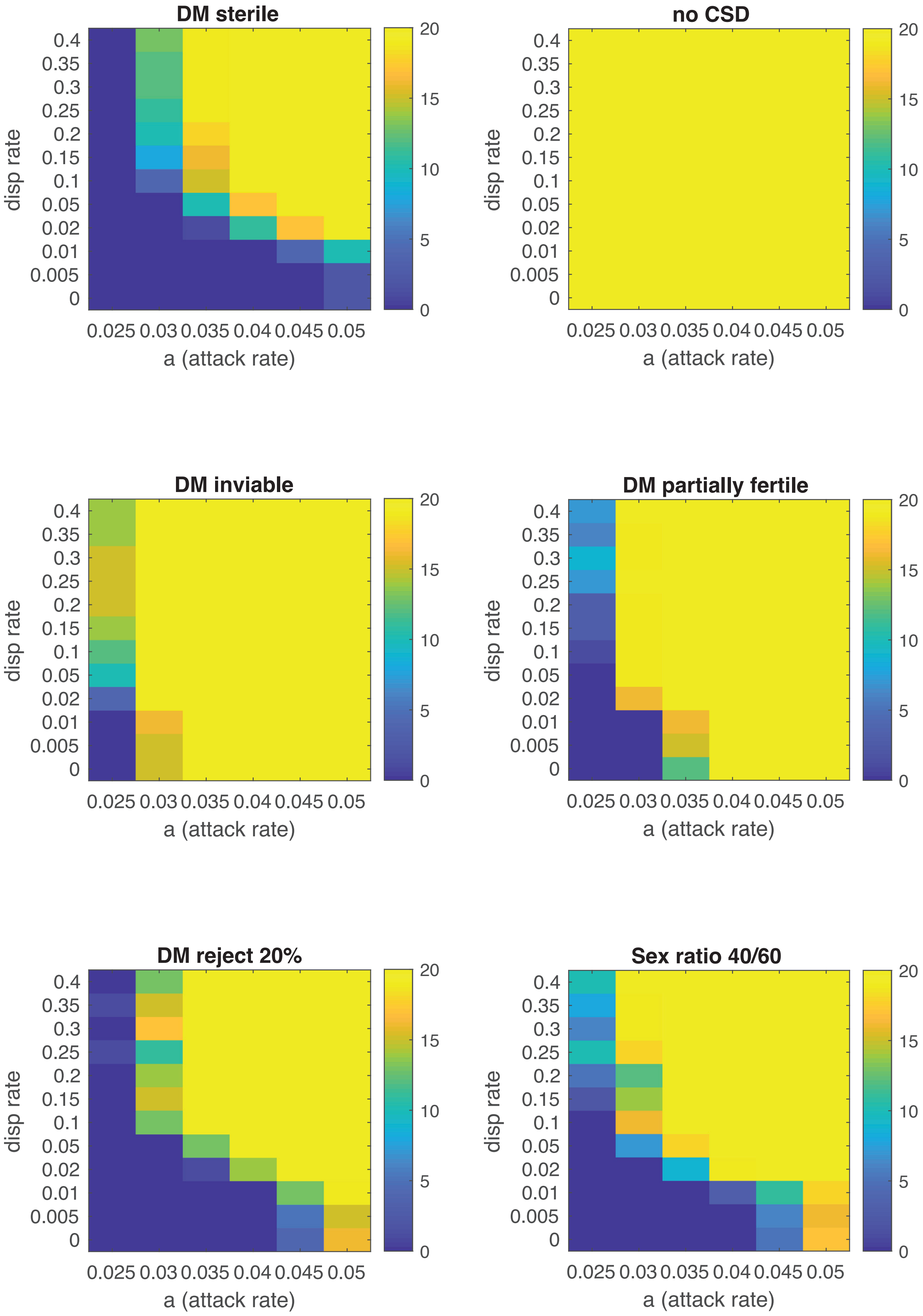

Model 5: small fluctuation
Population persistence

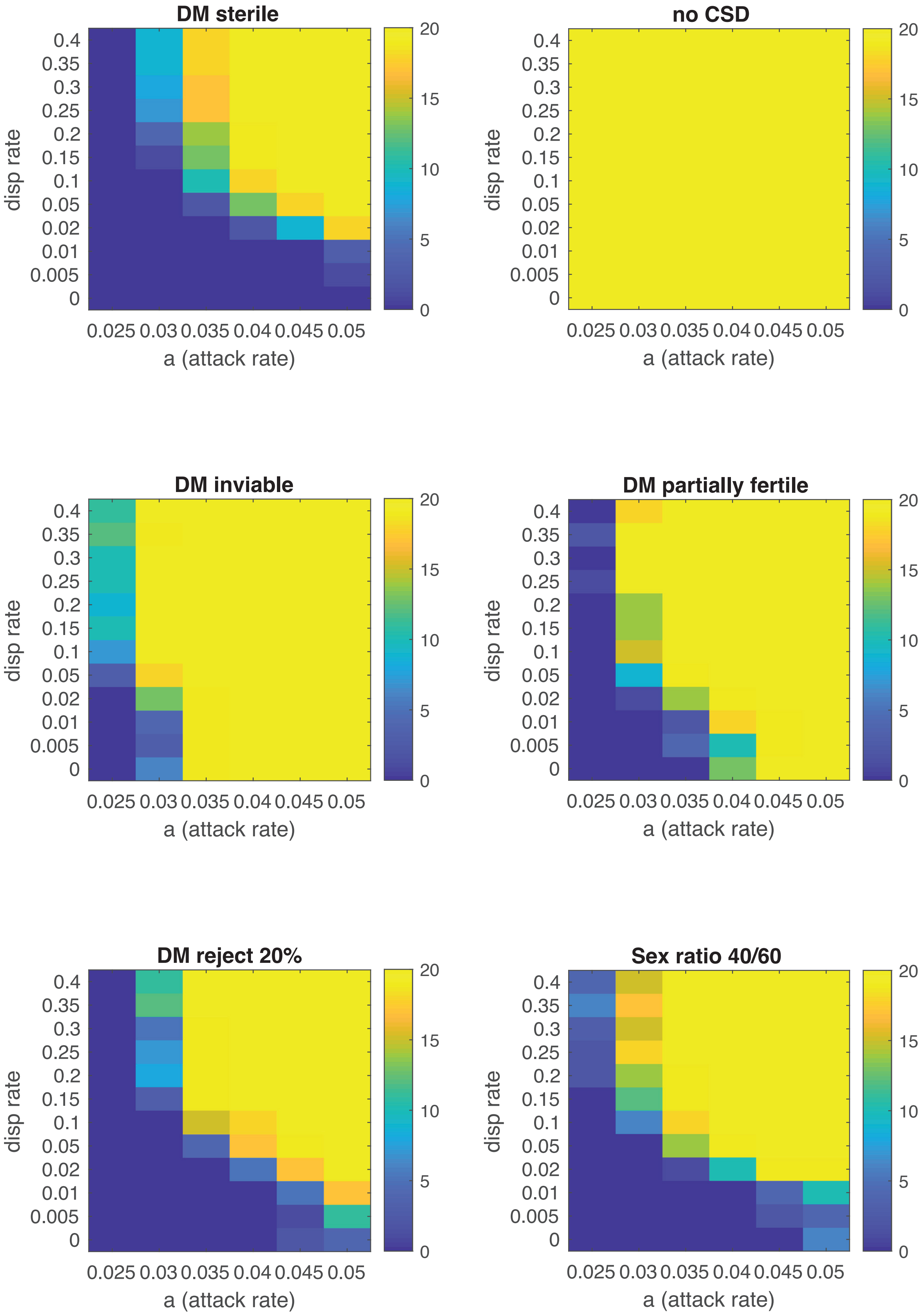

Model 5: large fluctuation
Population persistence

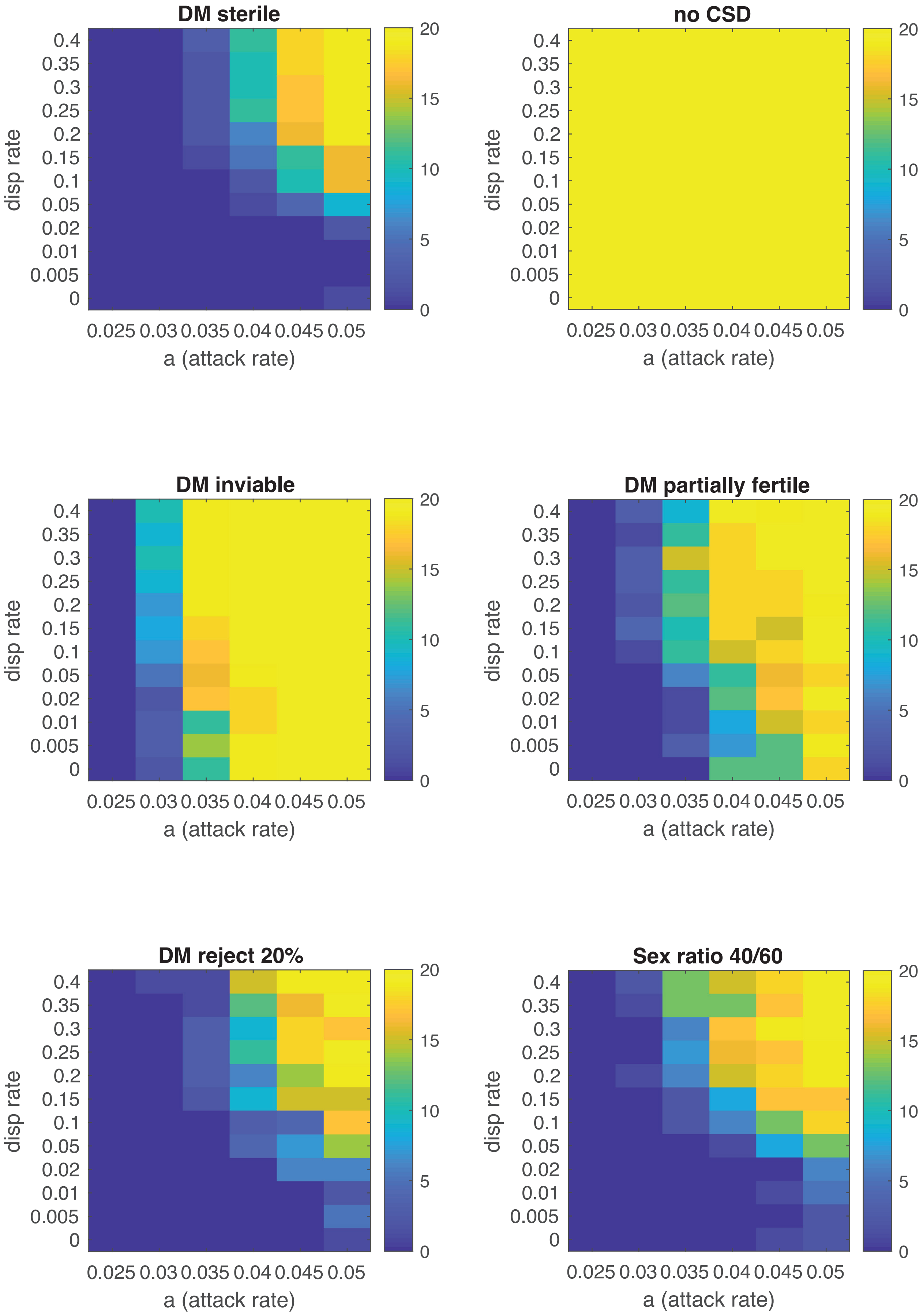

Model 5: spatially autocorrelated large fluctuation
Population persistence

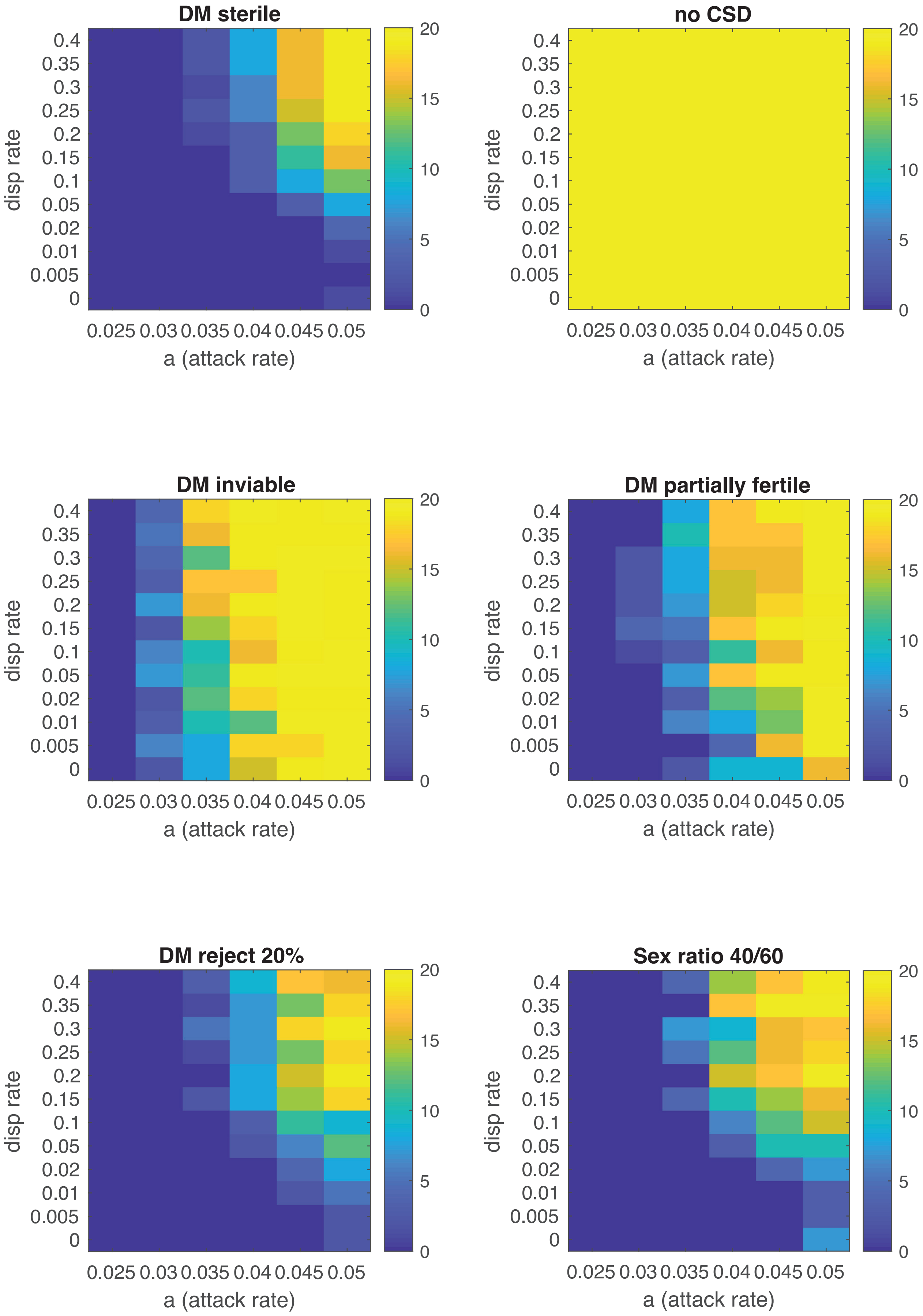

Model 5: large rednoise
Population persistence

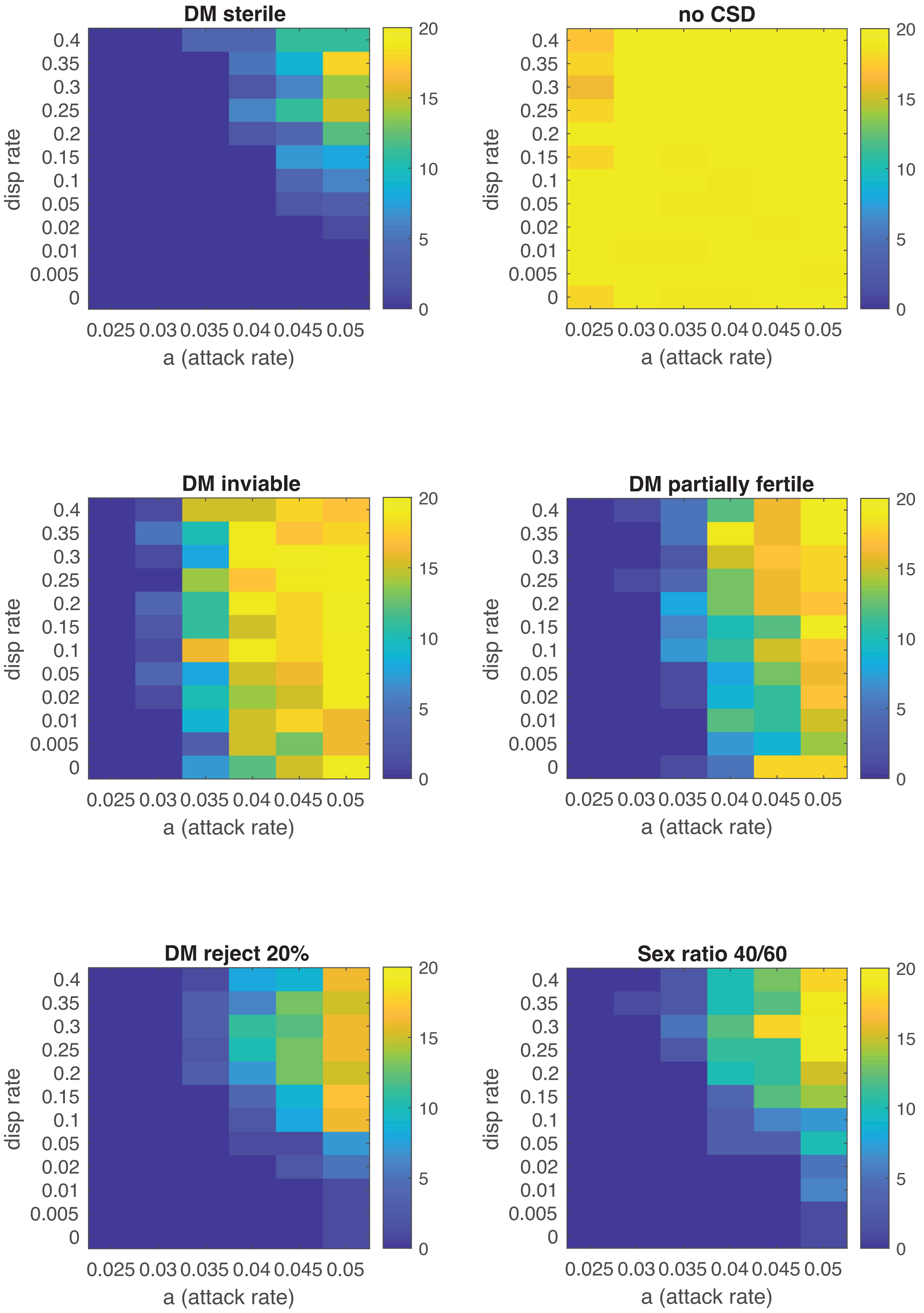

Model 6: no added fluctuation
Population persistence

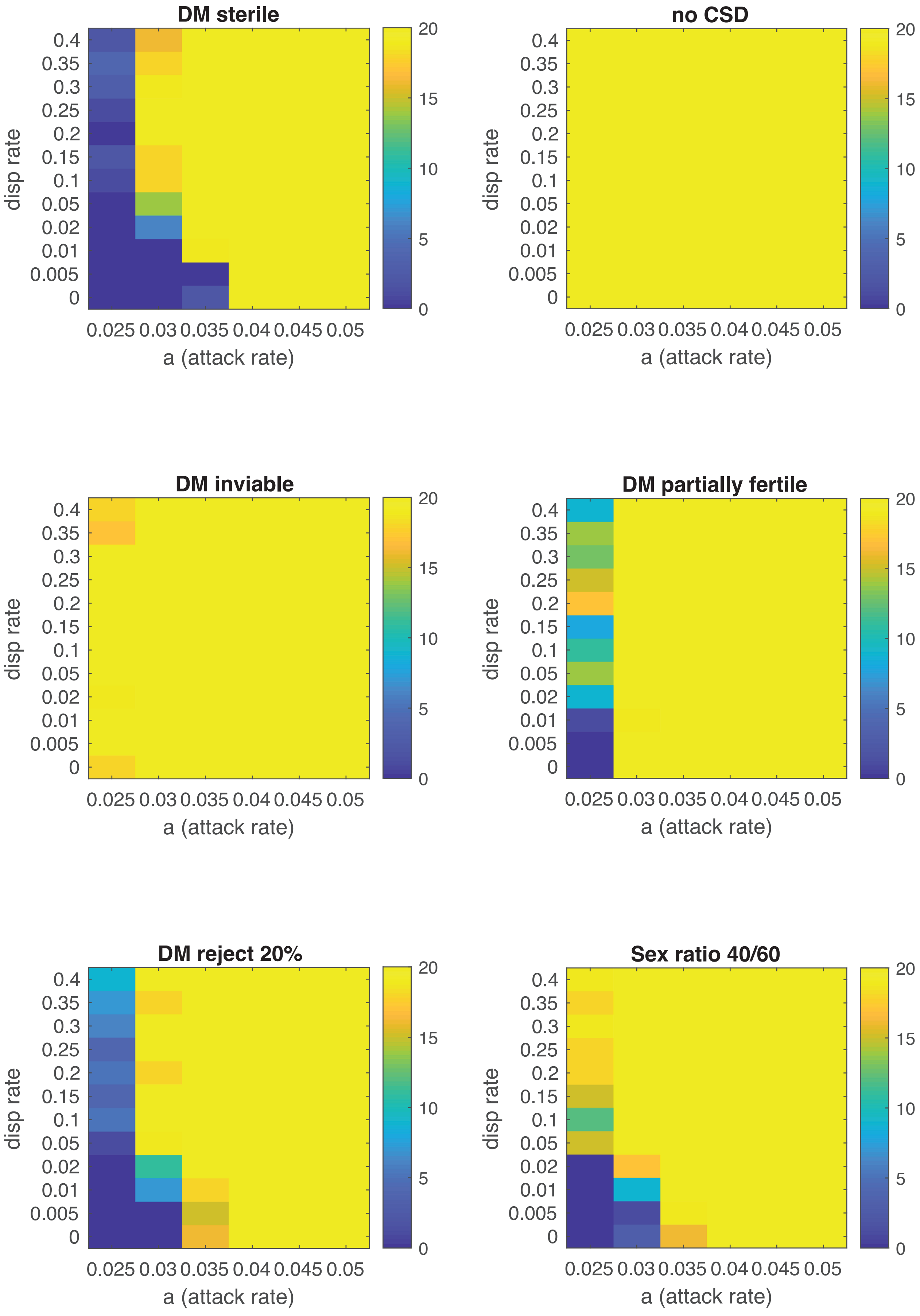

Model 6: small fluctuation
Population persistence

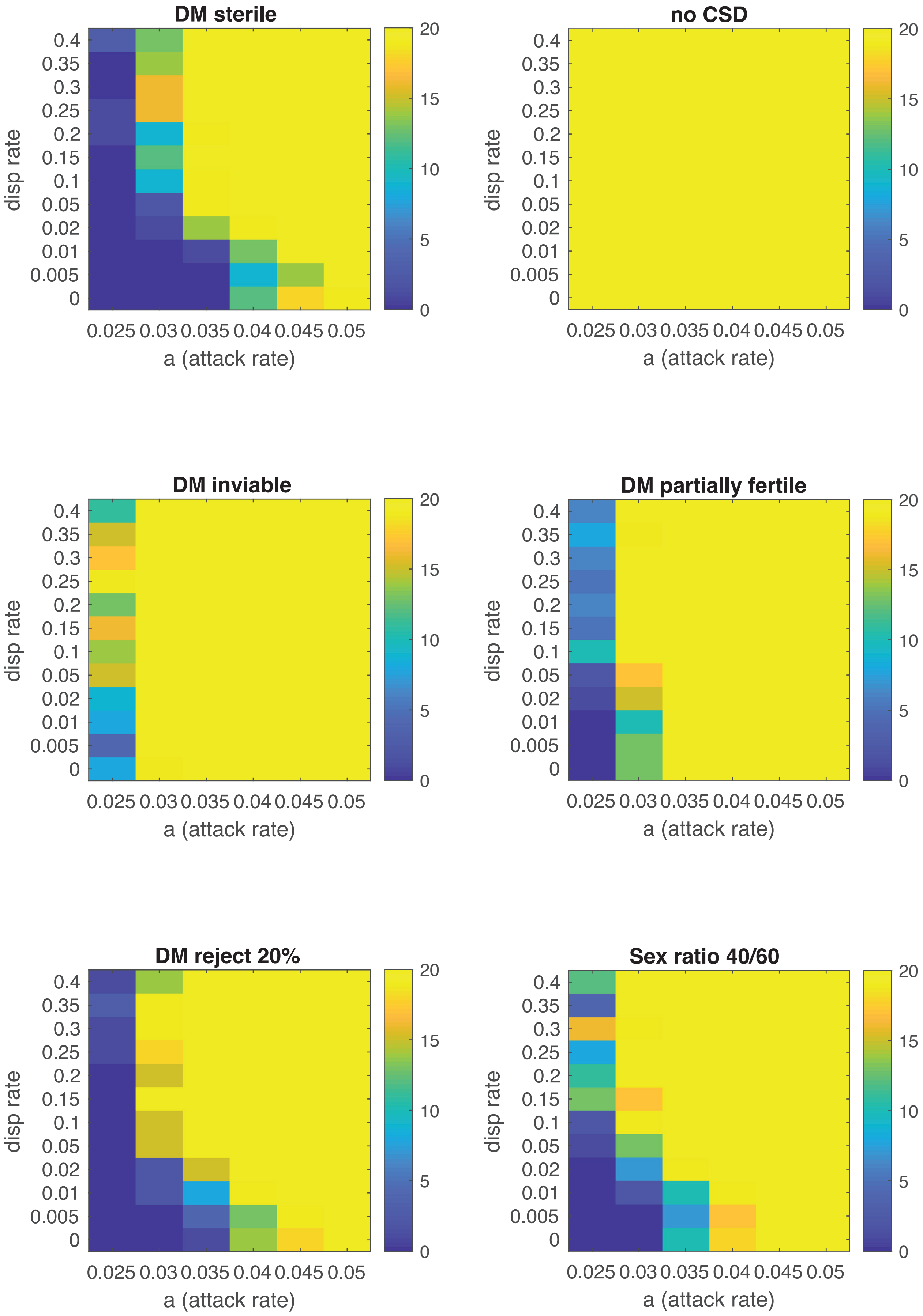

Model 6: large fluctuation
Population persistence

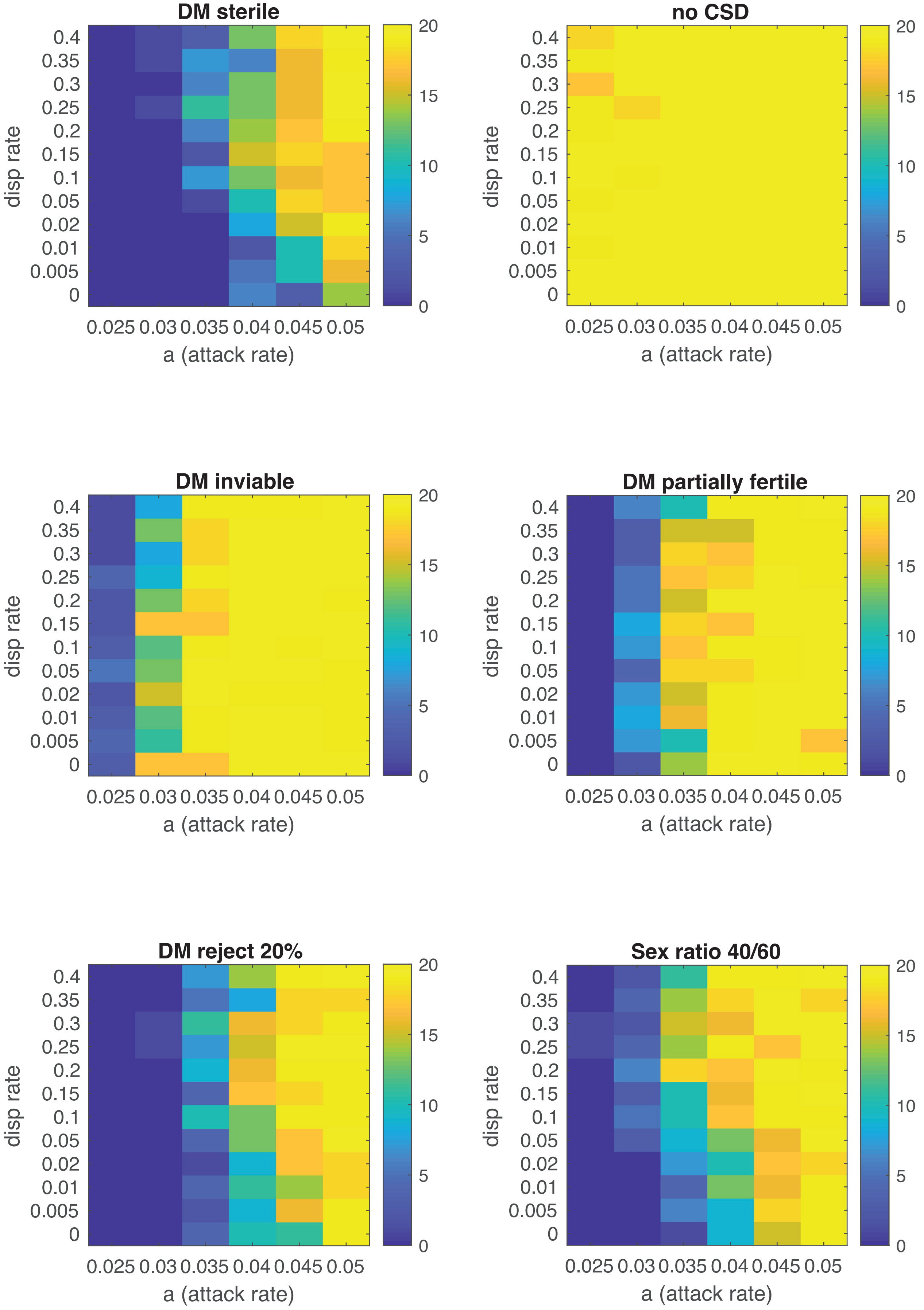

Model 6: spatially autocorrelated large fluctuation
Population persistence

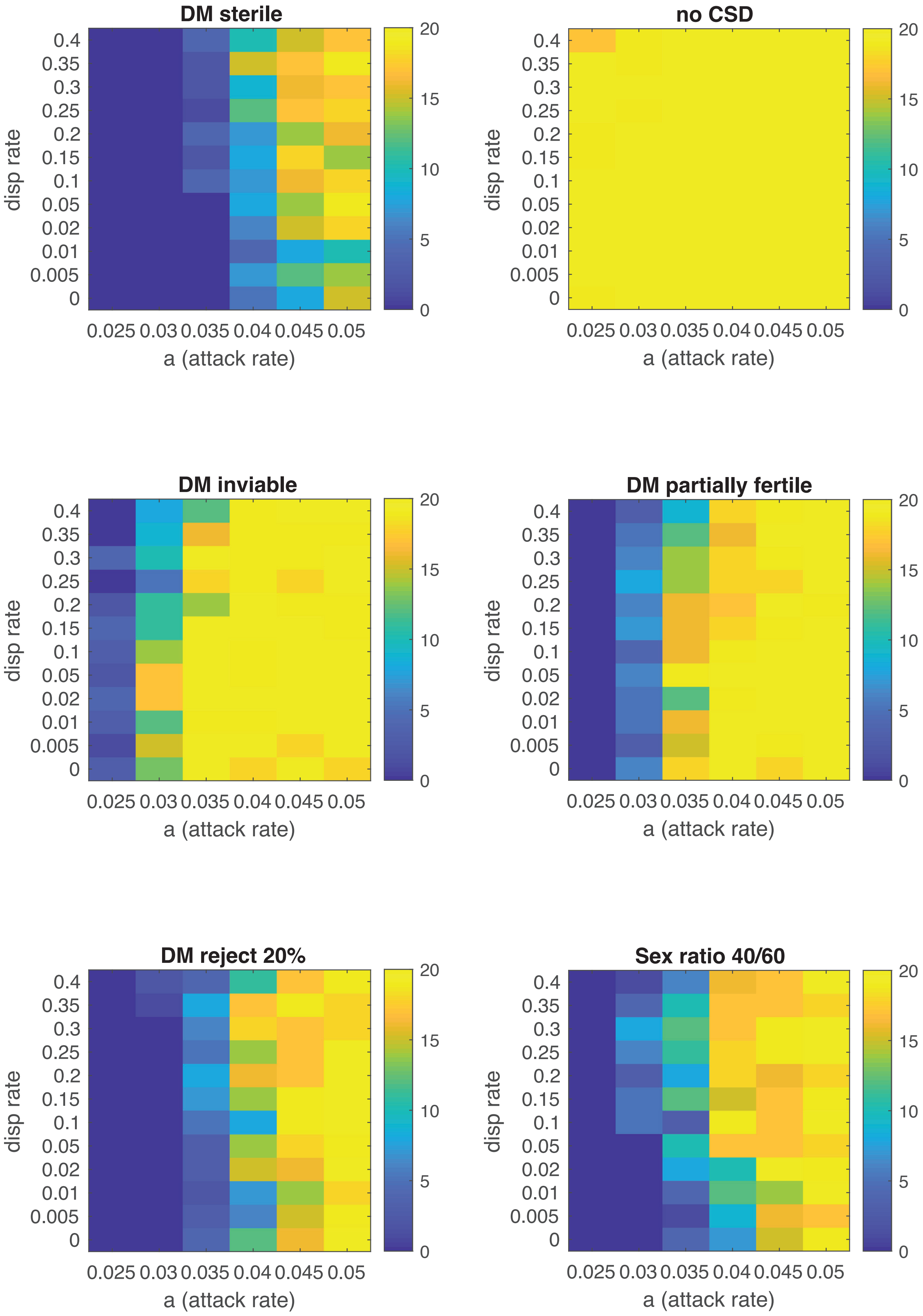

Model 6: large rednoise
Population persistence

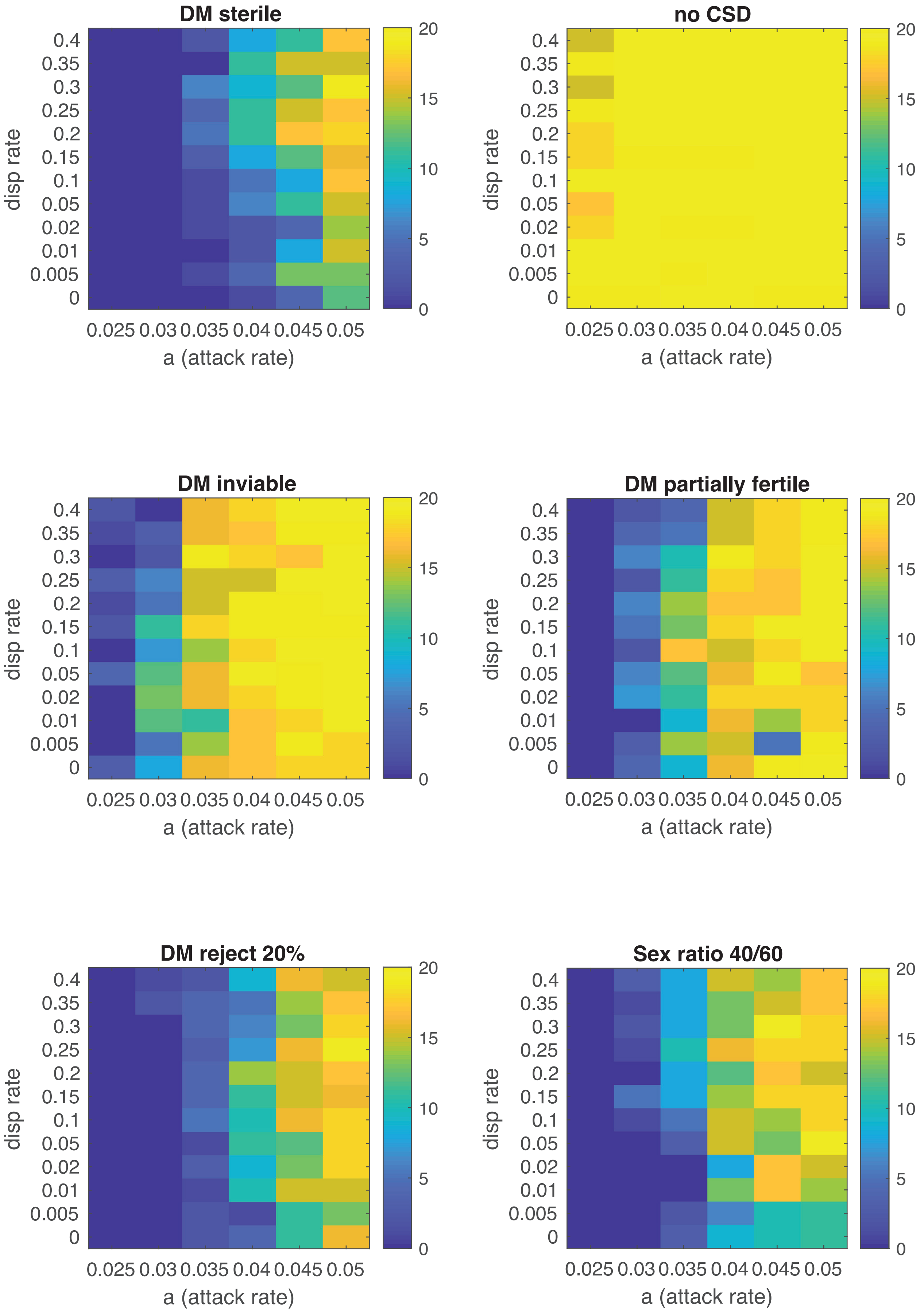

Figure S3. The number of alleles present at the end of simulations. The populations are initiated with 20 CSD alleles. Because no mutation is assumed, the max number of alleles is 20 and the number only declined over time. In Figure 4 in the main text, the same data are presented by dispersal rate.

Model 1: no added fluctuation
Number of CSD alleles

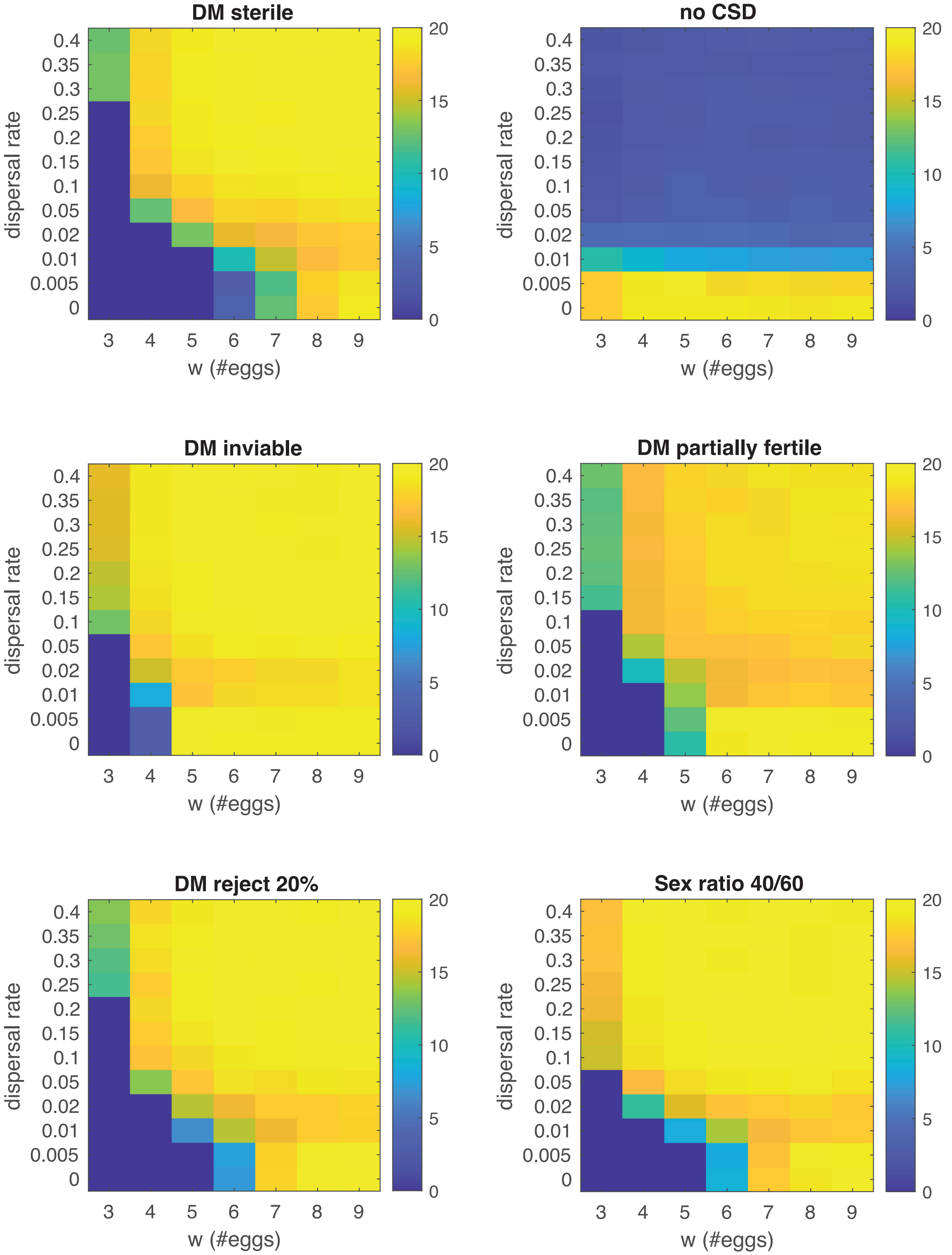

Model 1: small fluctuation
Number of CSD alleles

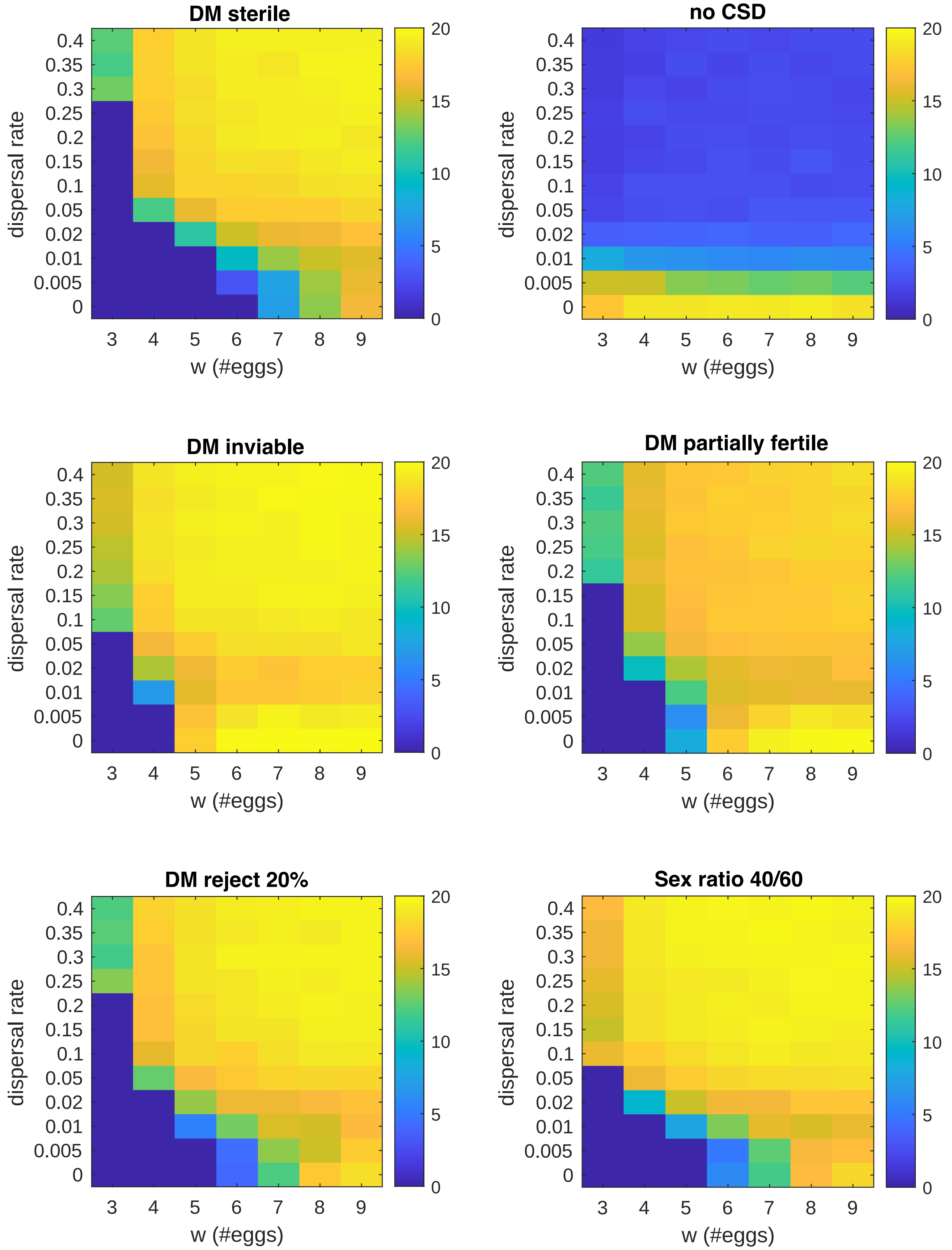

Model 1: large fluctuation
Number of CSD alleles

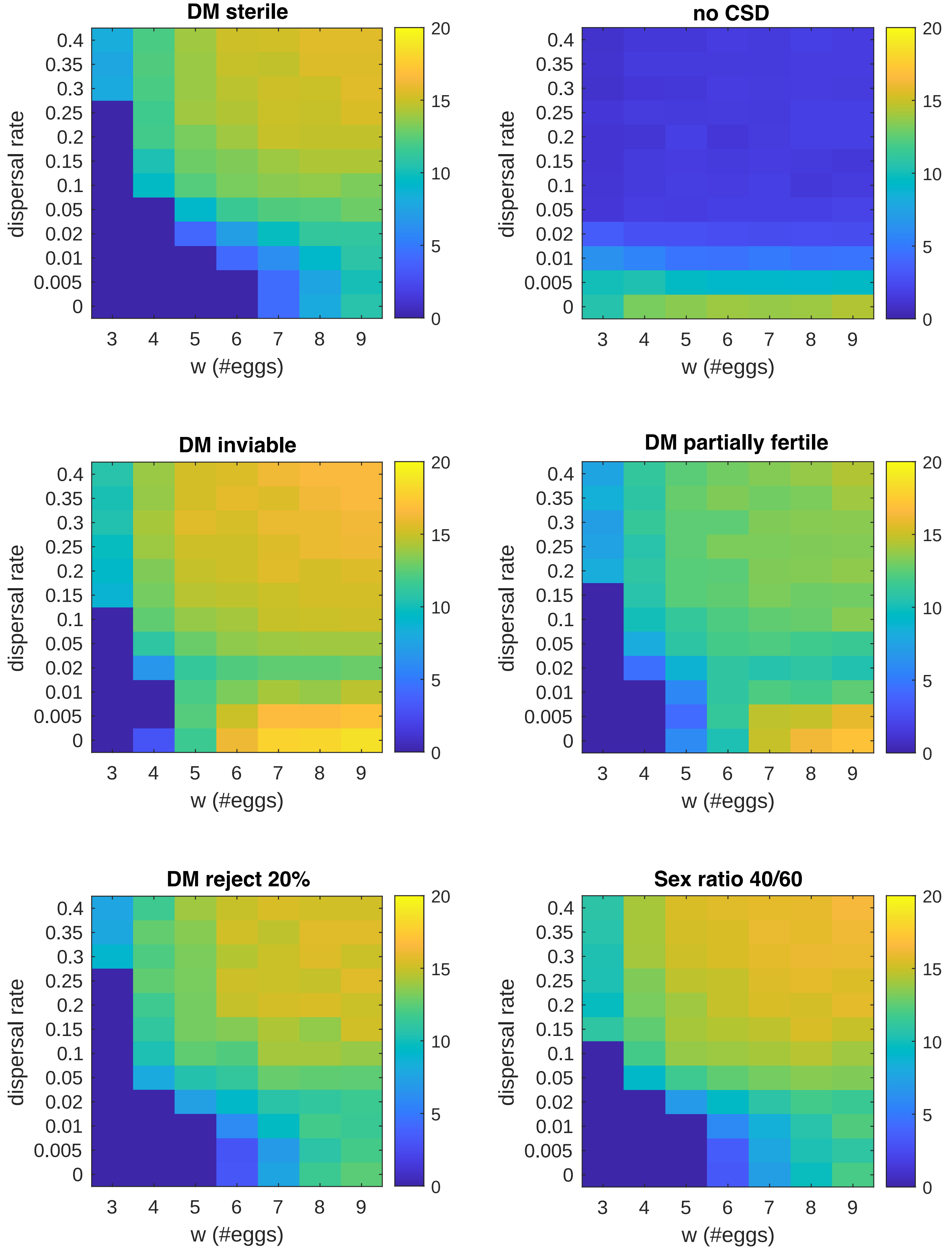

Model 1: spatially autocorrelated large fluctuation
Number of CSD alleles

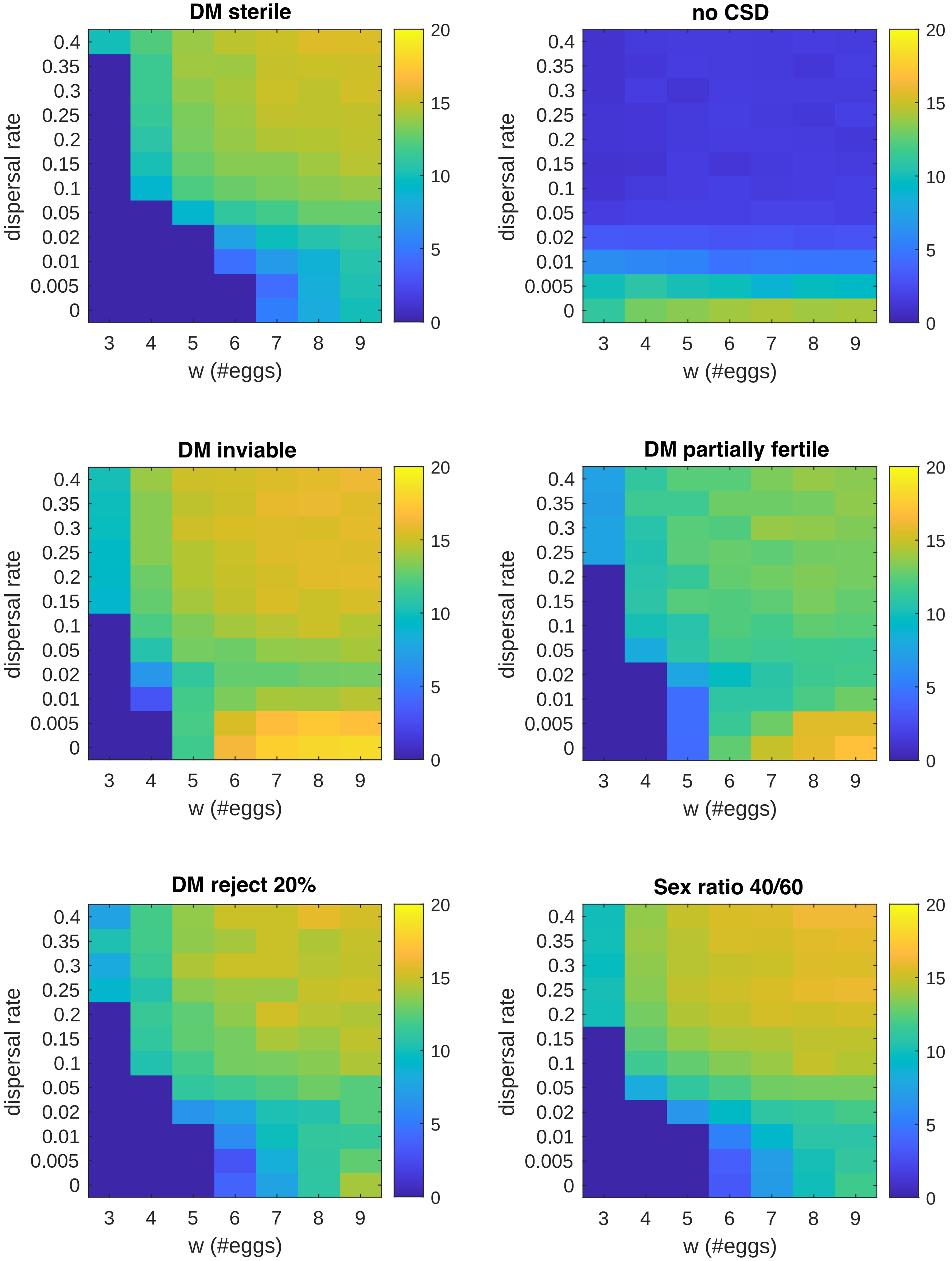

Model 1: large rednoise
Number of CSD alleles

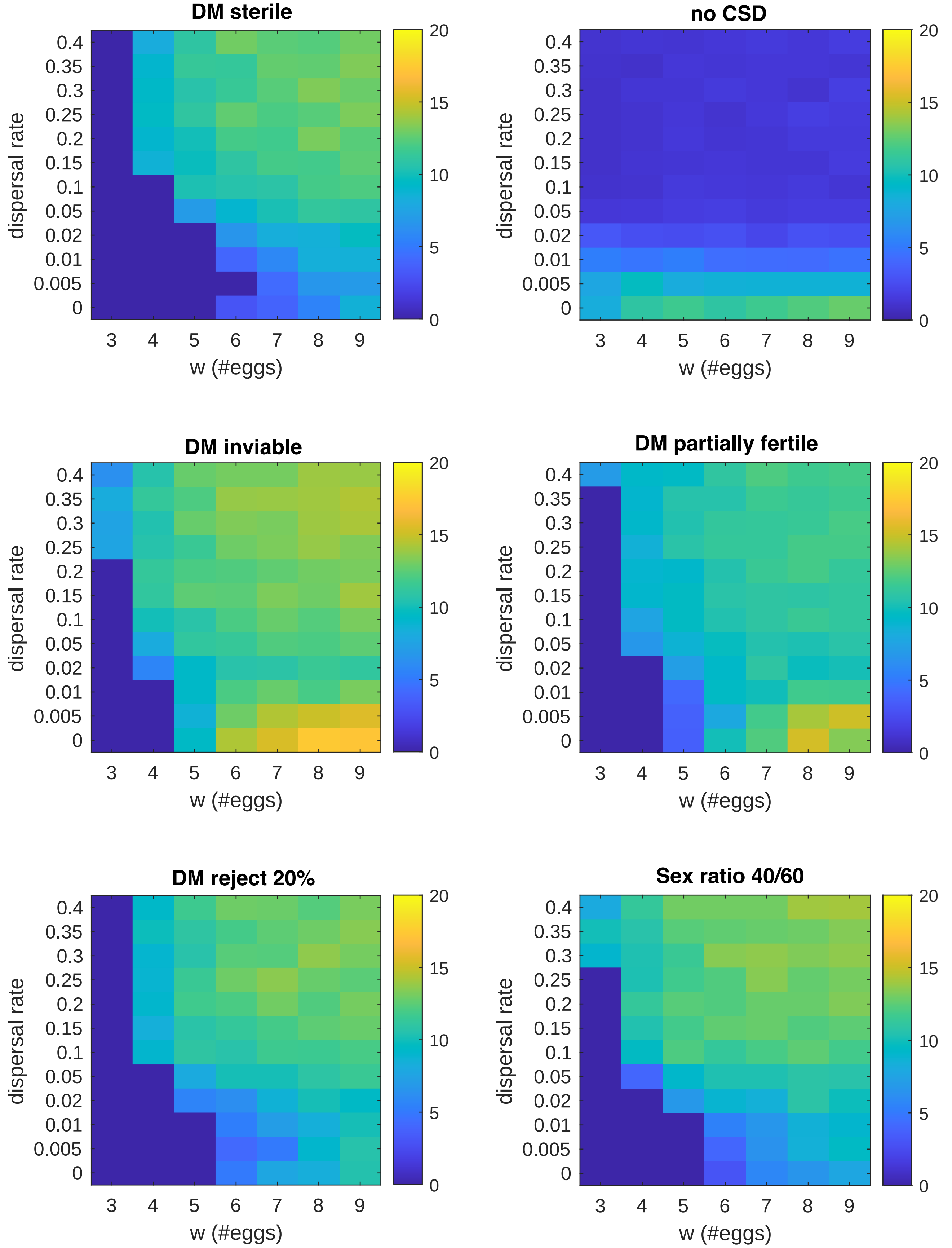

Model 2: no added fluctuation
Number of CSD alleles

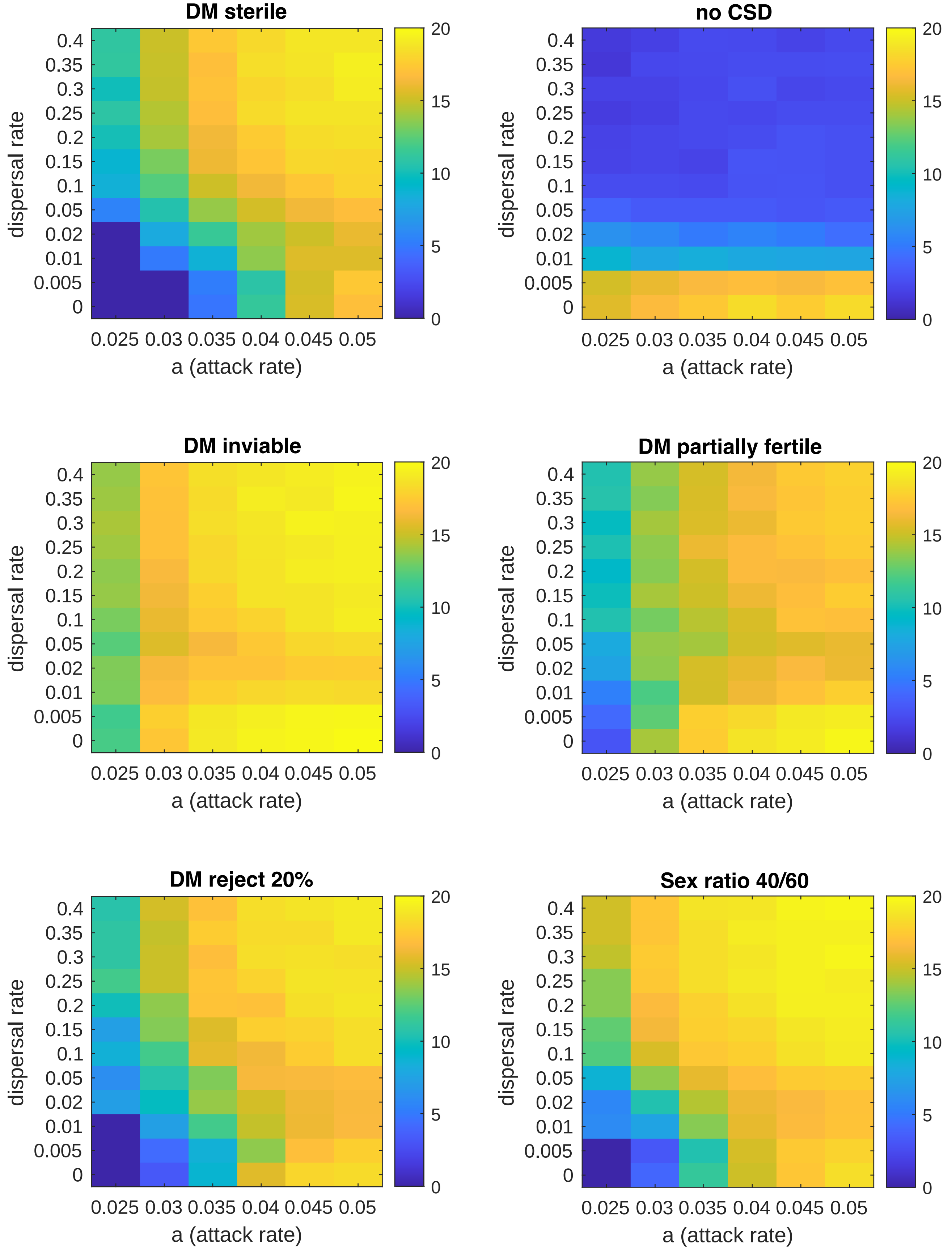

Model 2: small fluctuation
Number of CSD alleles

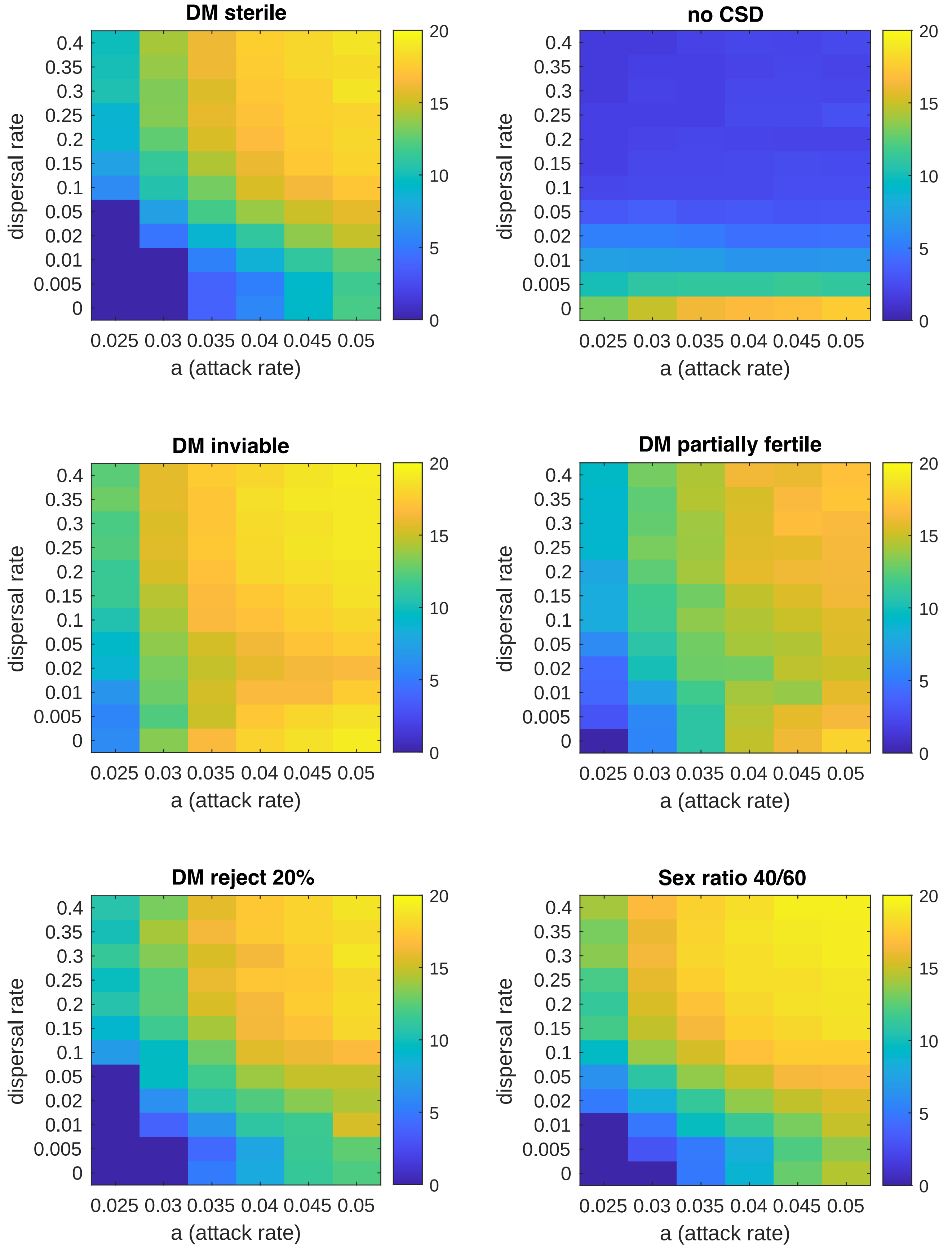

Model 2: large fluctuation
Number of CSD alleles

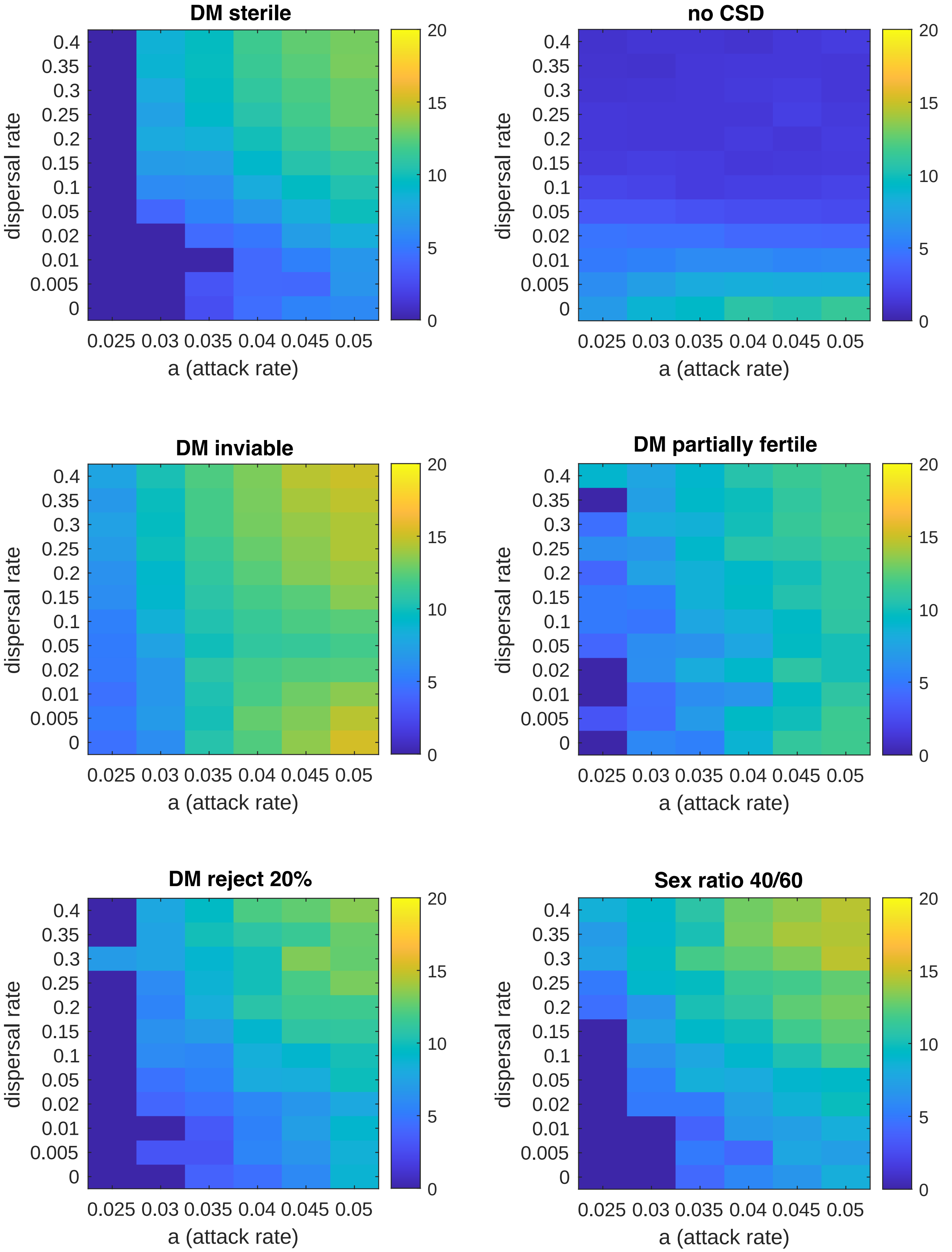

Model 2: spatially autocorrelated large fluctuation
Number of CSD alleles

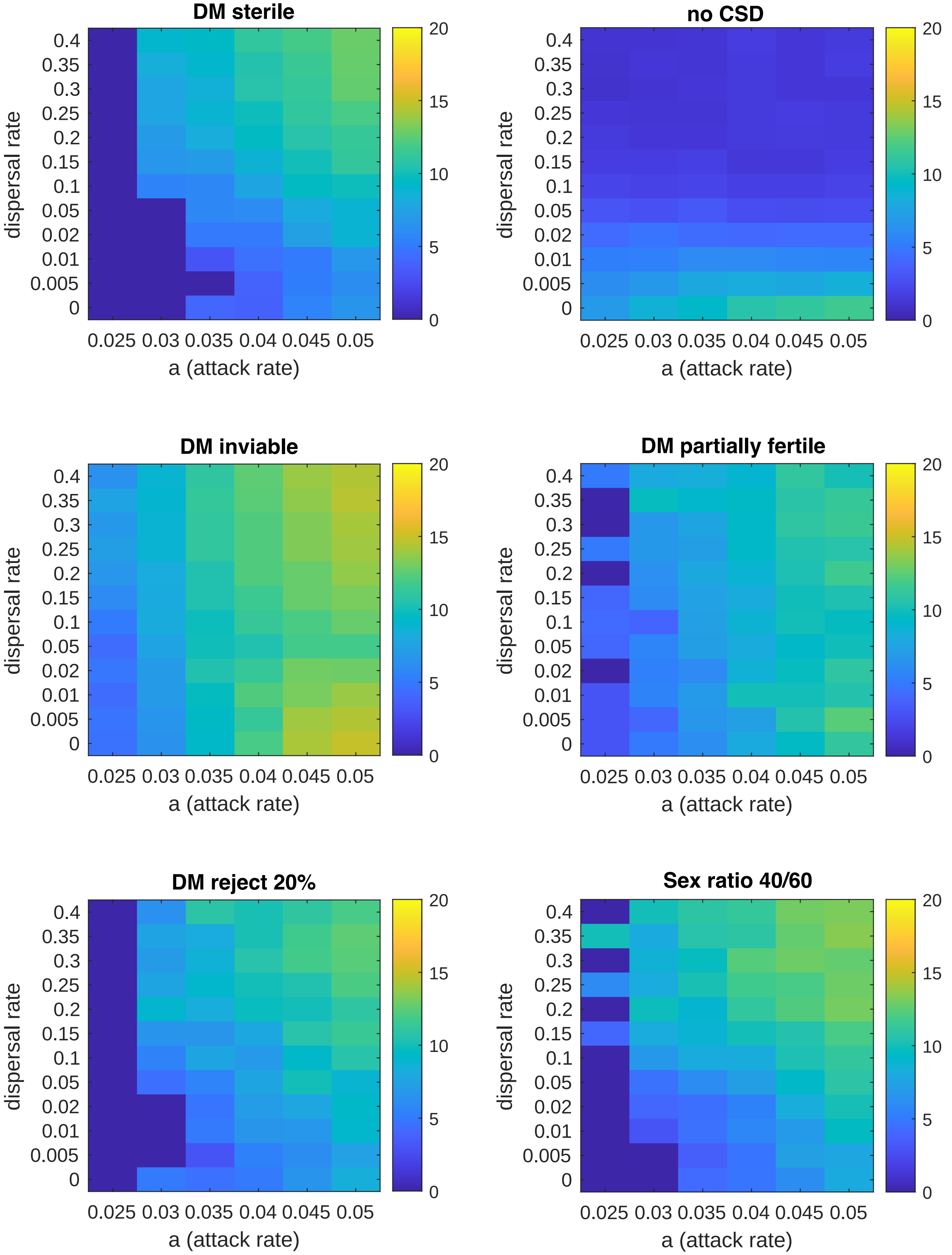

Model 2: large rednoise
Number of CSD alleles

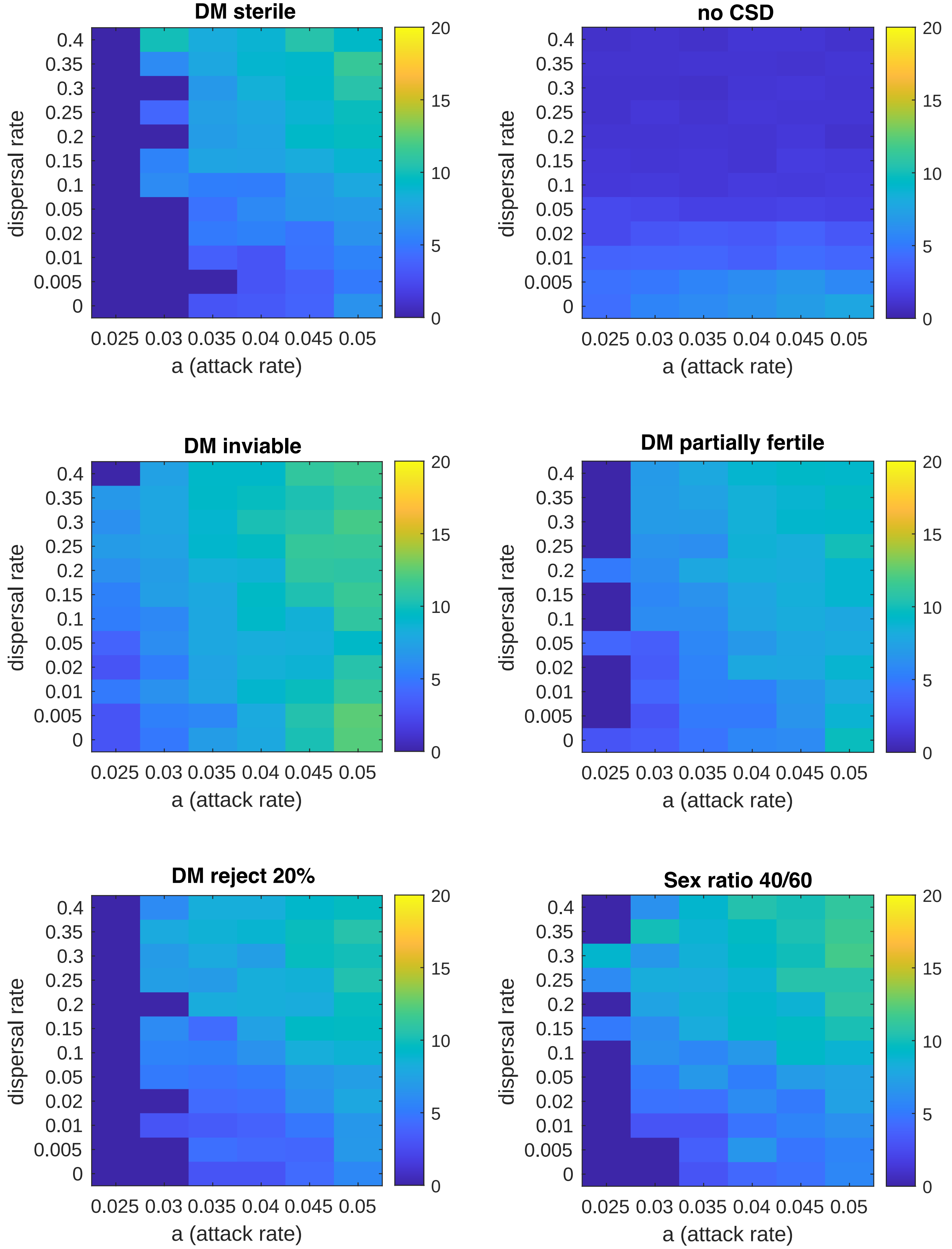

Model 3: no added fluctuation
Number of CSD alleles

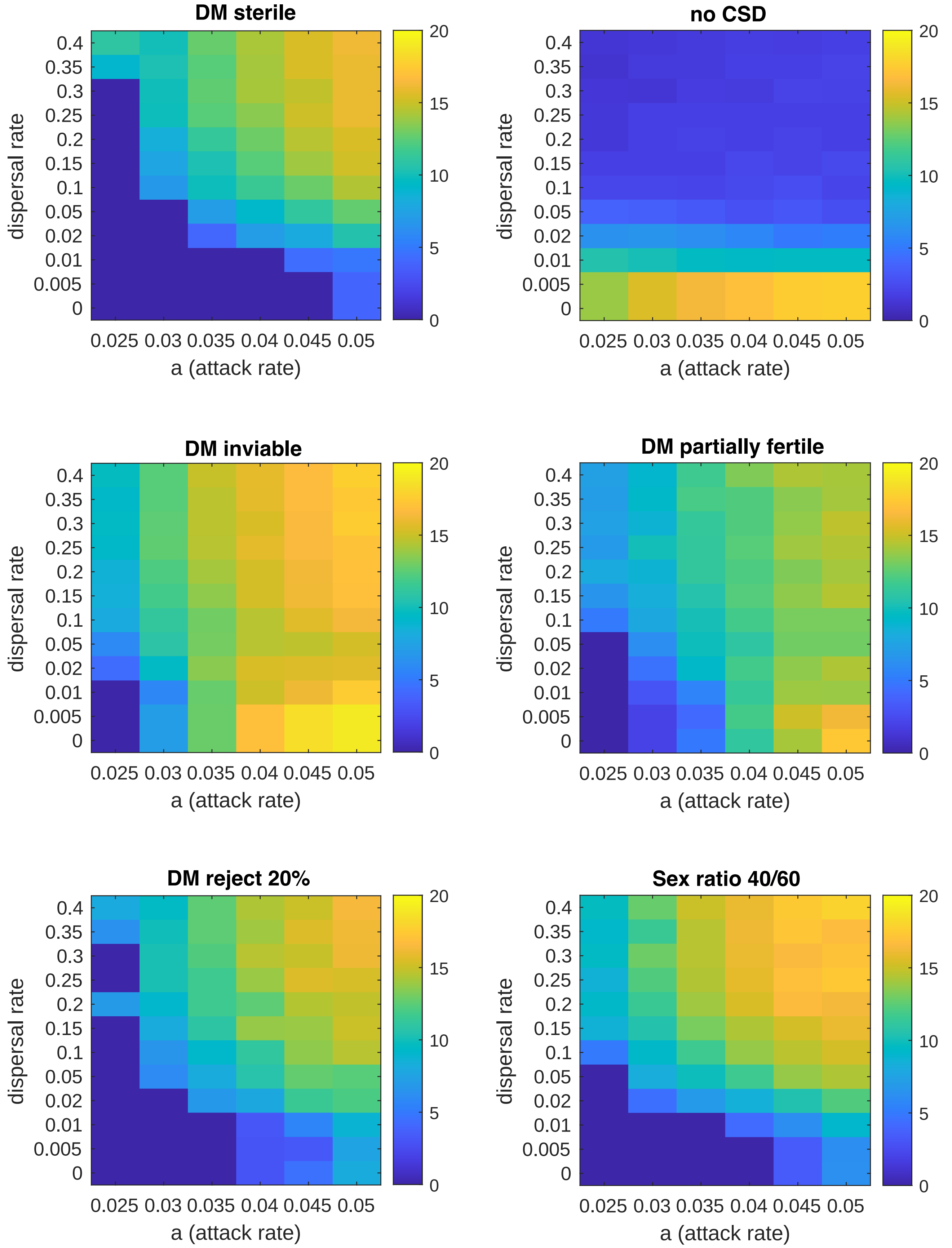

Model 3: small fluctuation
Number of CSD alleles

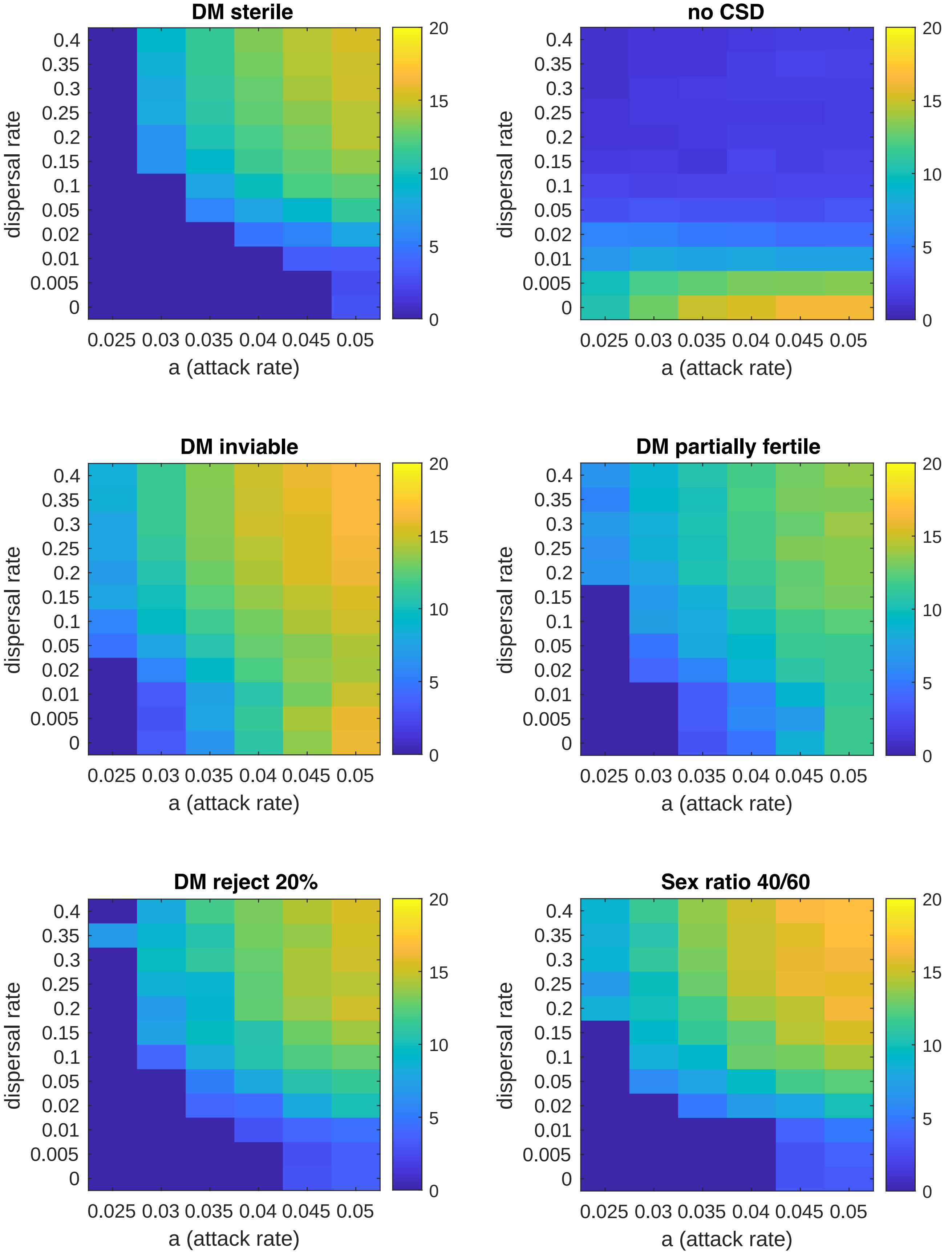

Model 3: large fluctuation
Number of CSD alleles

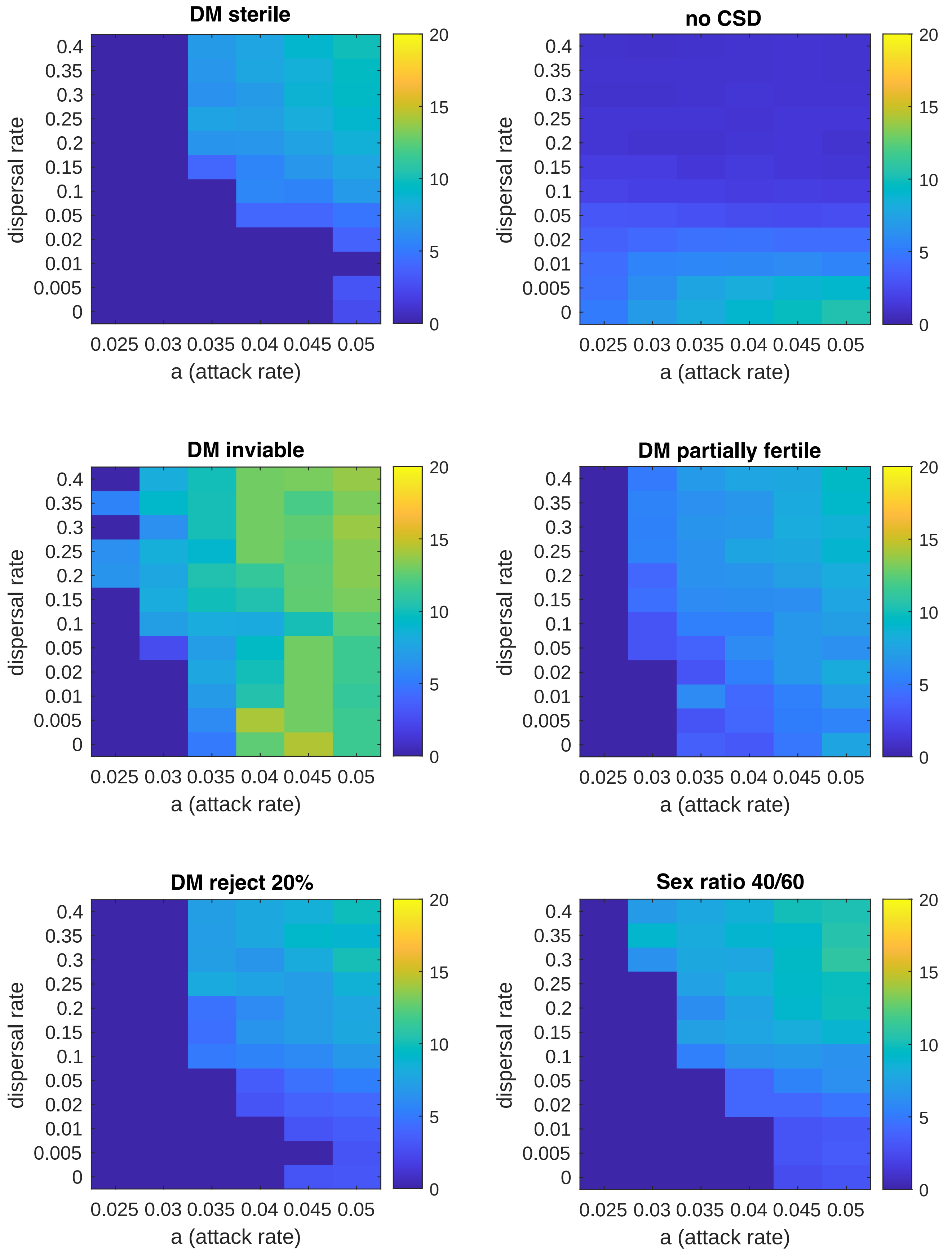

Model 3: spatially autocorrelated large fluctuation
Number of CSD alleles

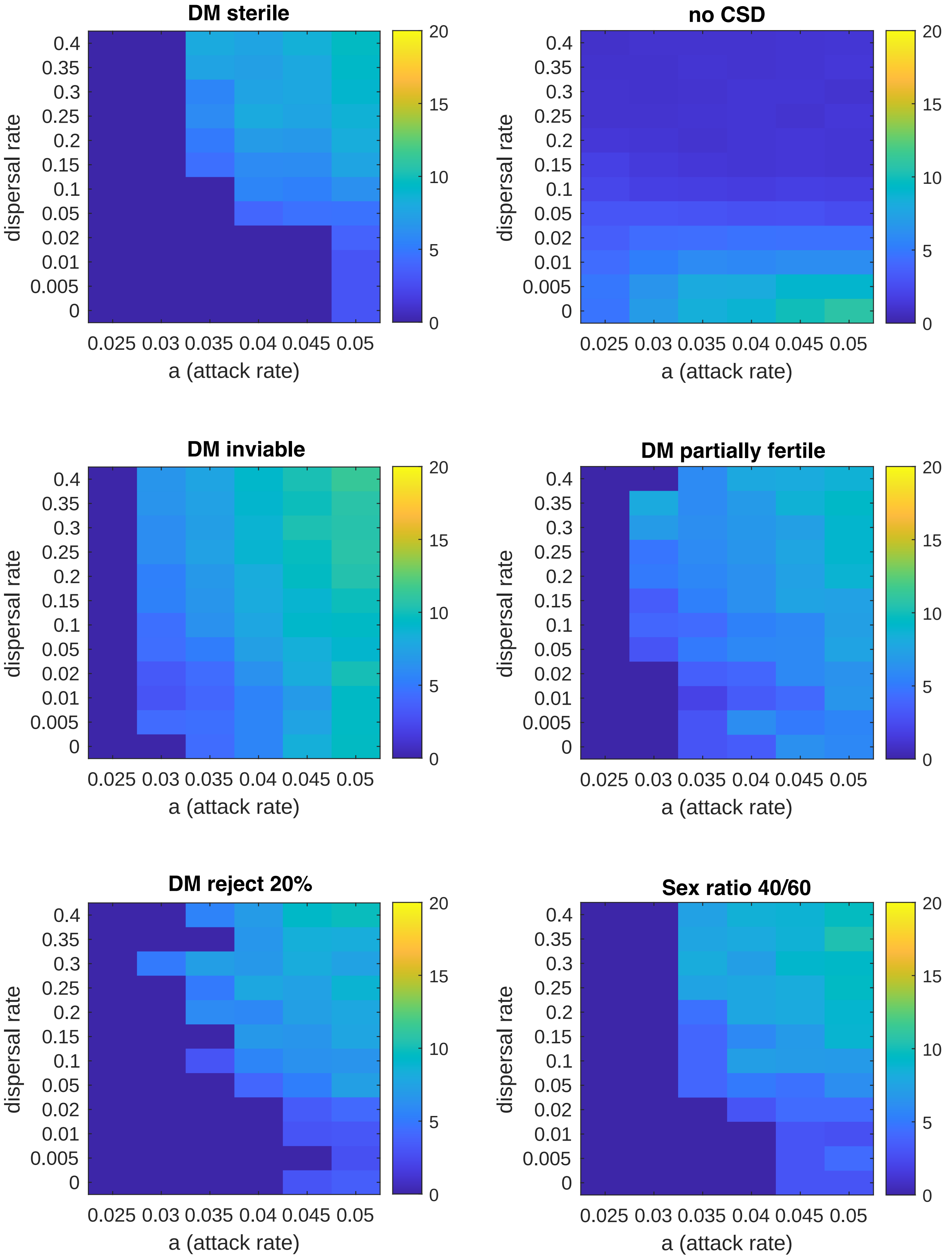

Model 3: large rednoise
Number of CSD alleles

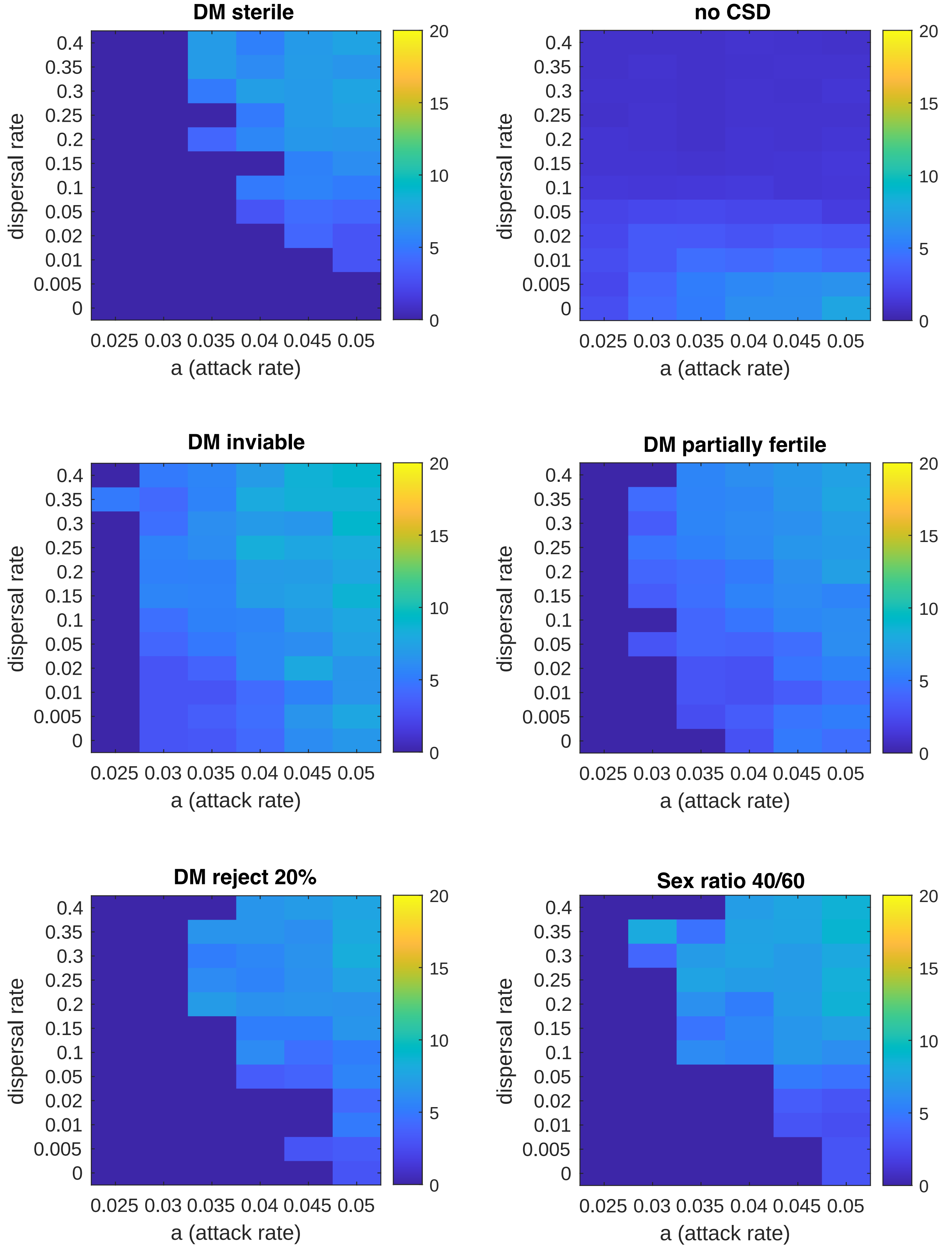

Model 4: no added fluctuation
Number of CSD alleles

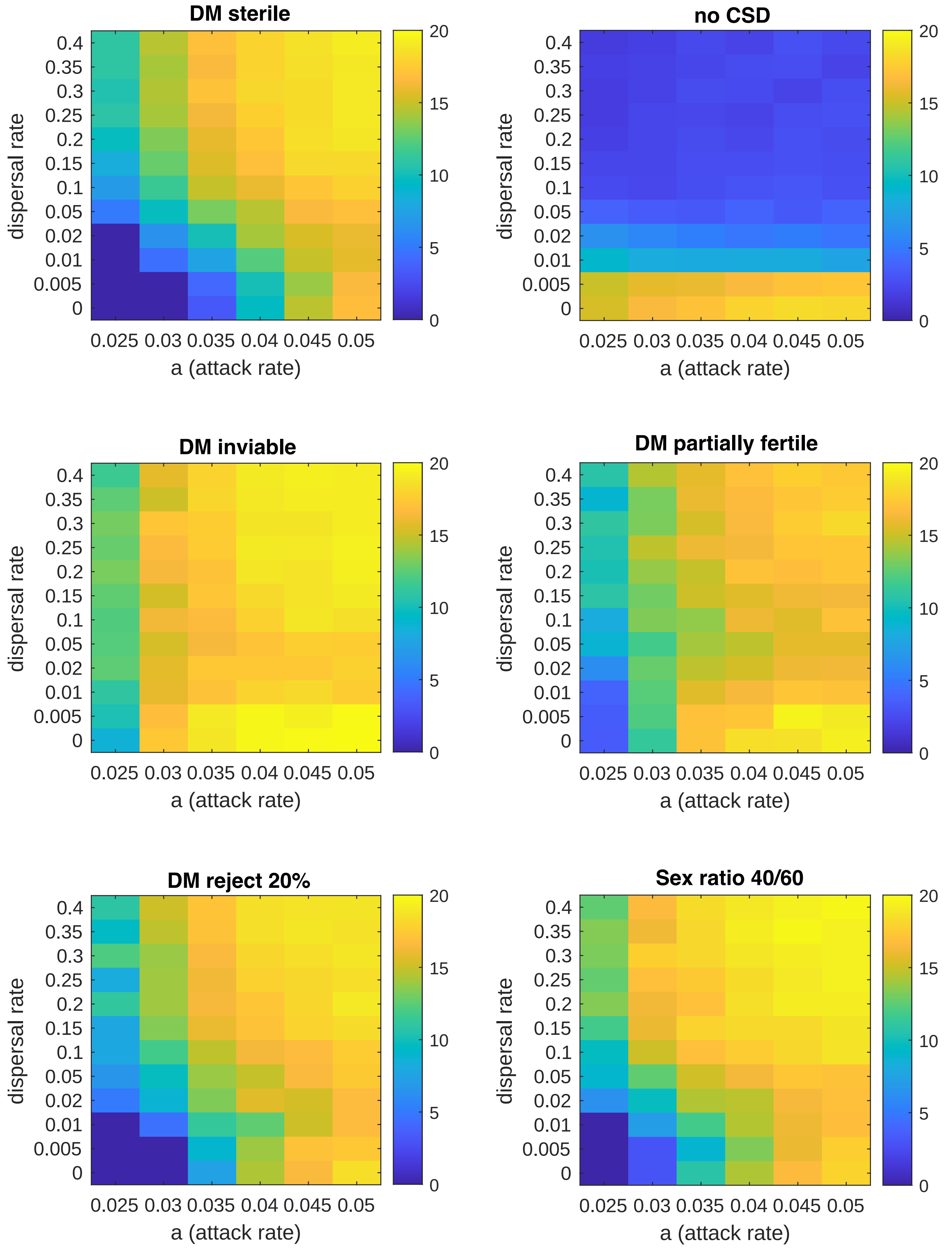

Model 4: small fluctuation
Number of CSD alleles

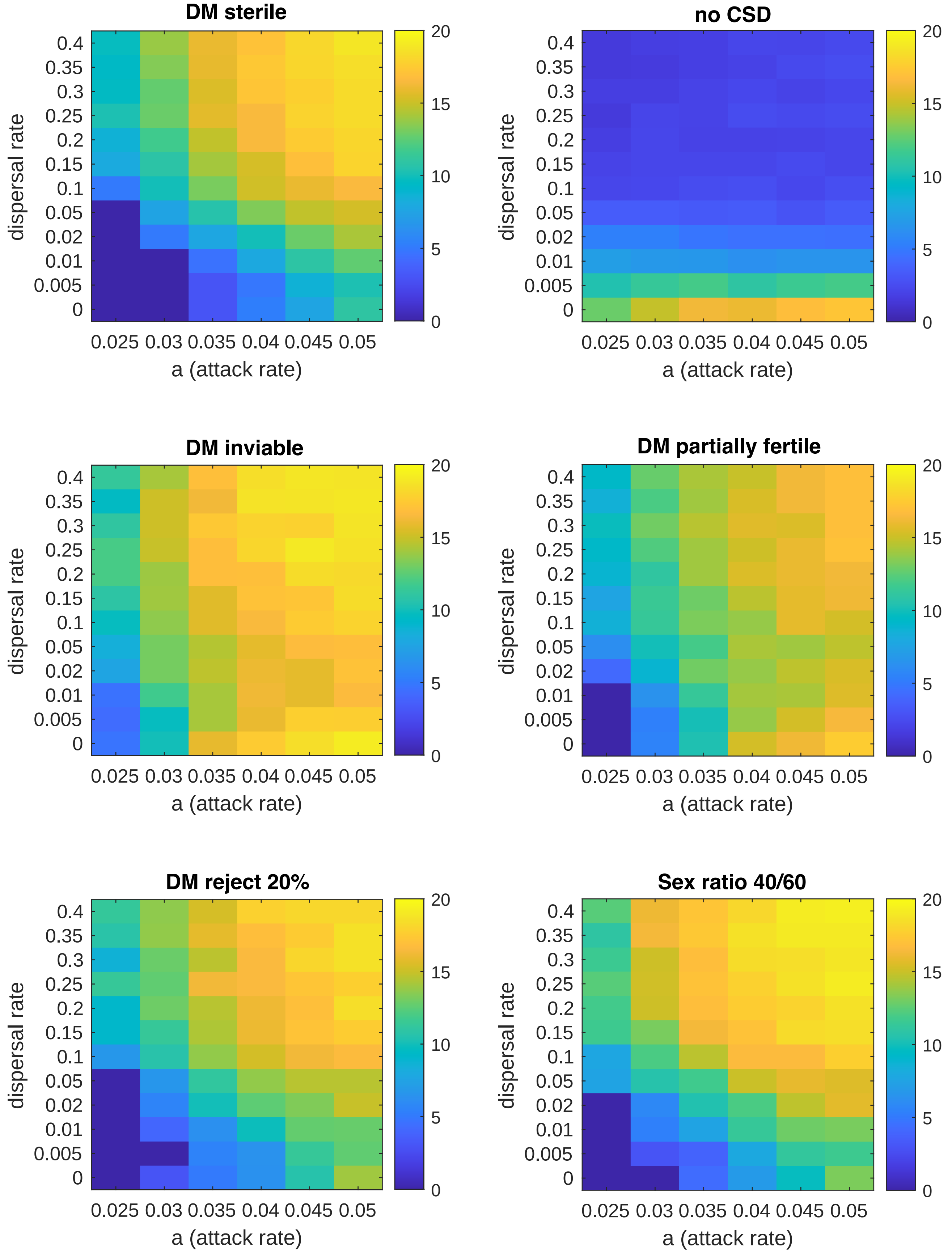

Model 4: large fluctuation
Number of CSD alleles

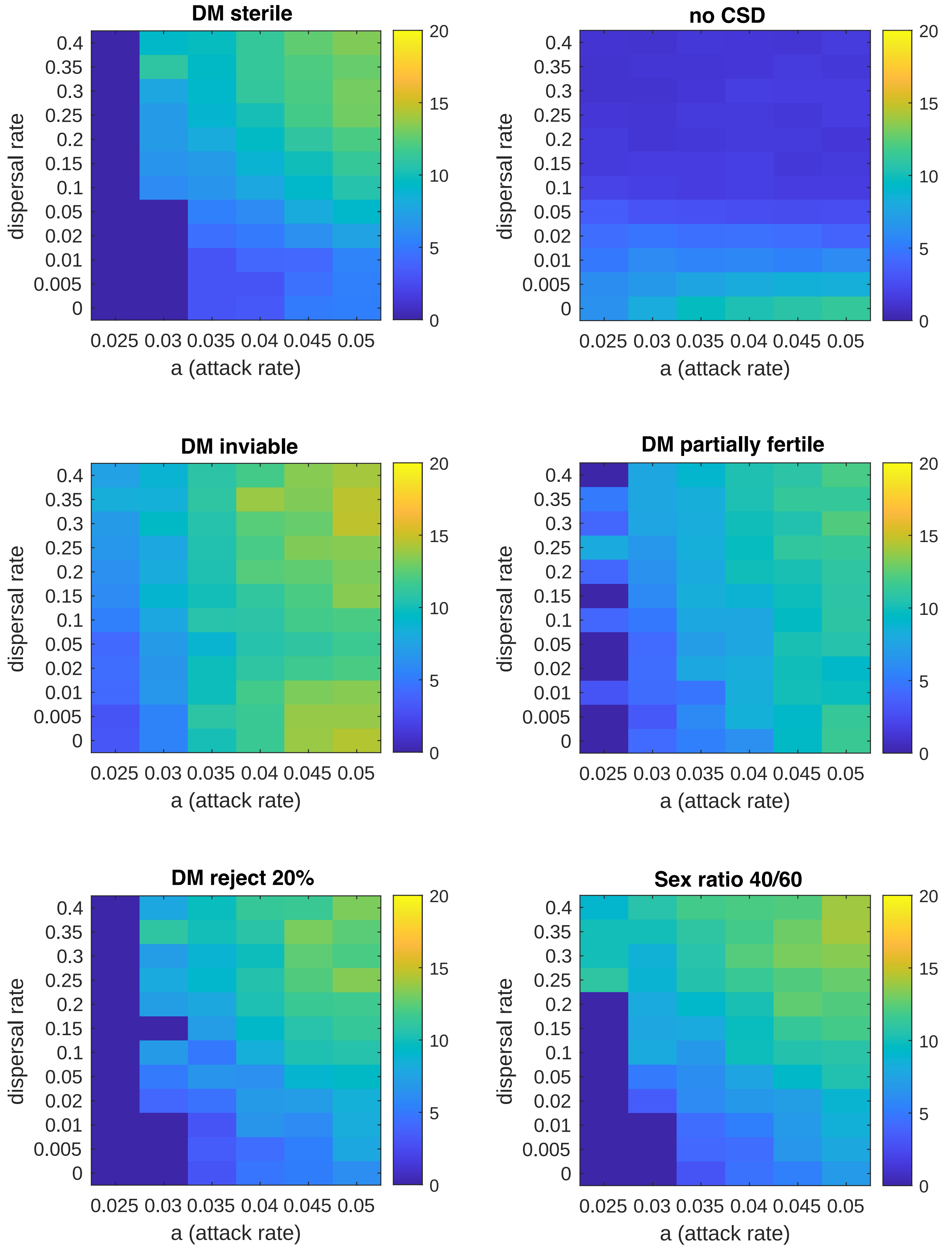

Model 4: spatially autocorrelated large fluctuation

Number of CSD alleles

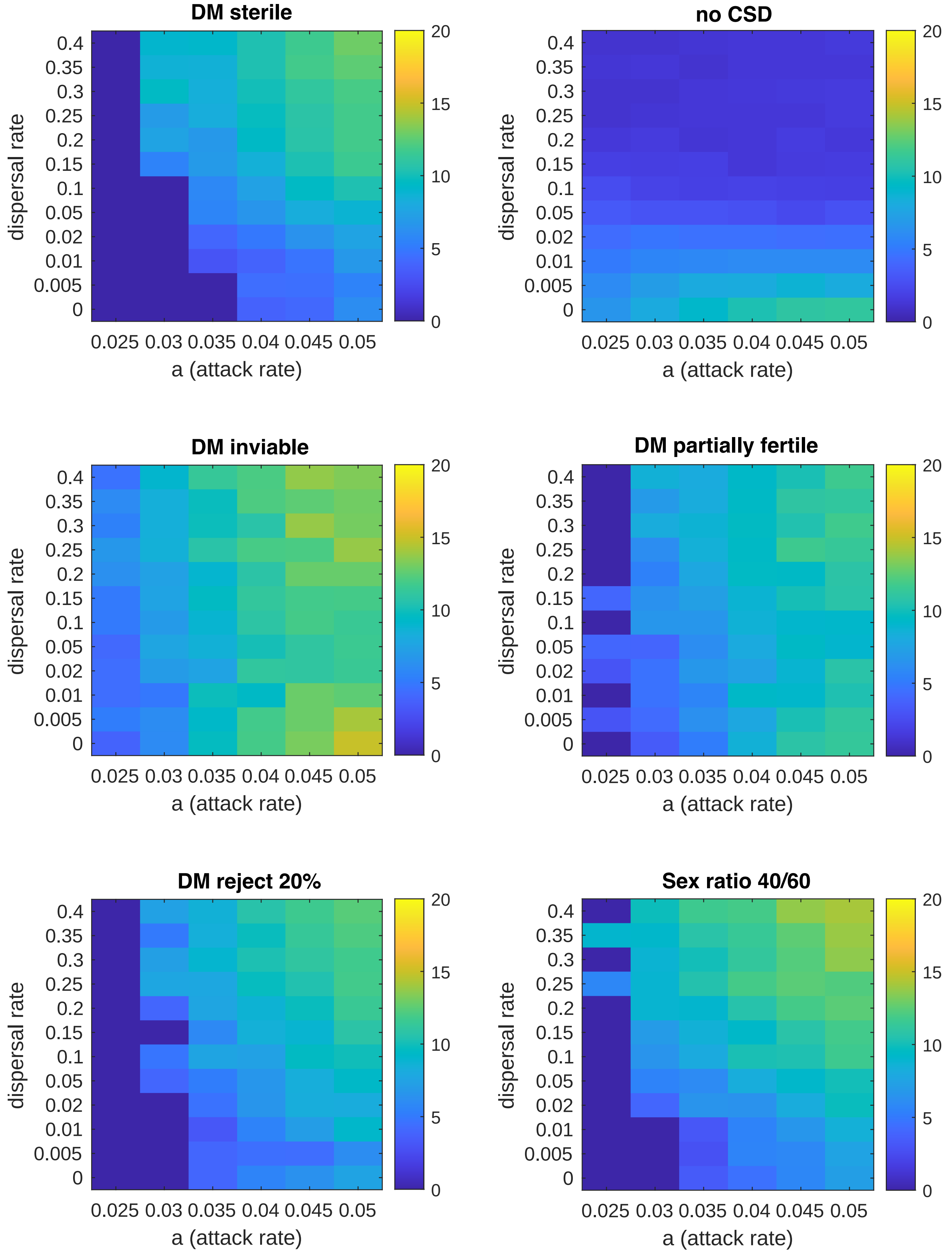

Model 4: large rednoise
Number of CSD alleles

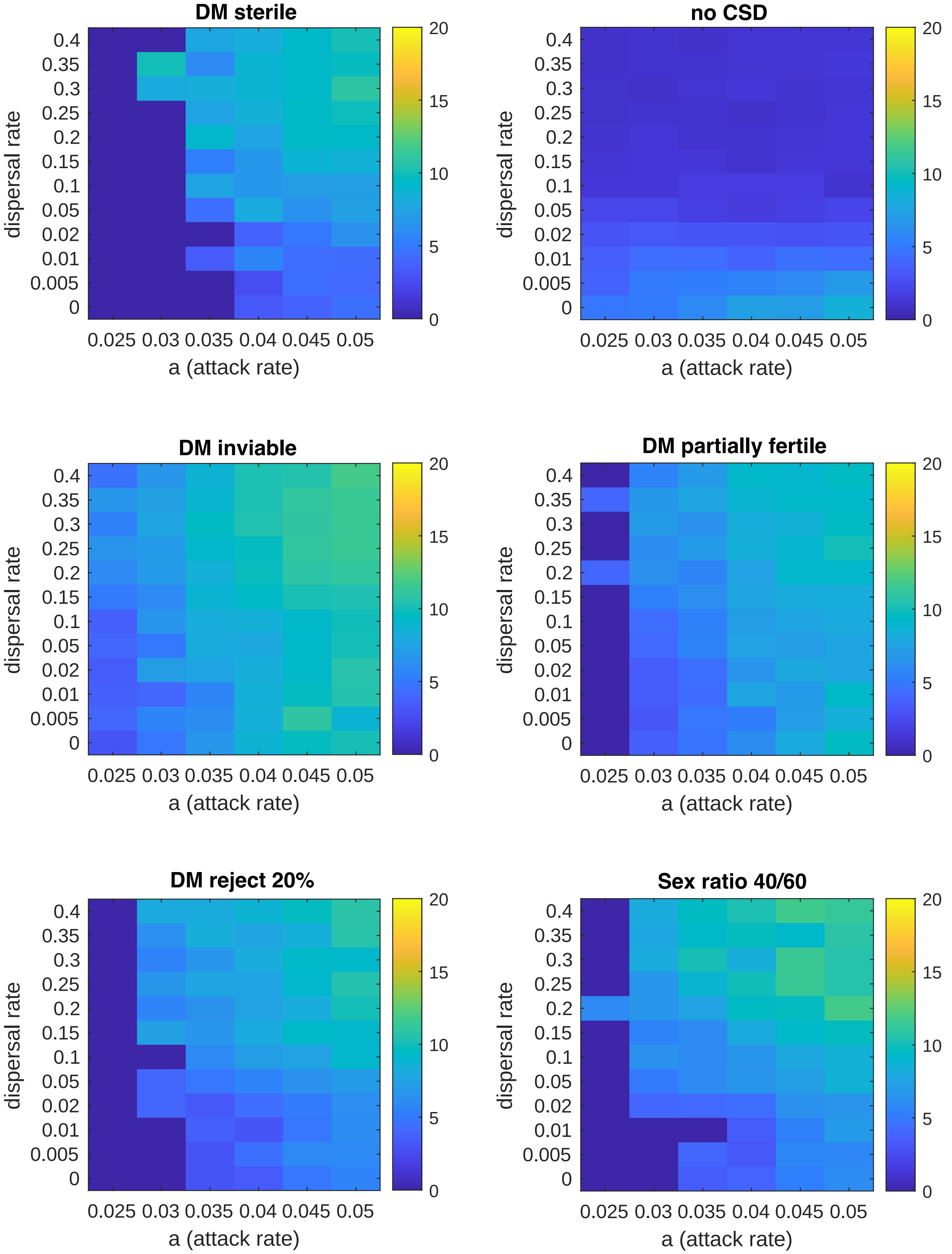

Model 5: no added fluctuation
Number of CSD alleles

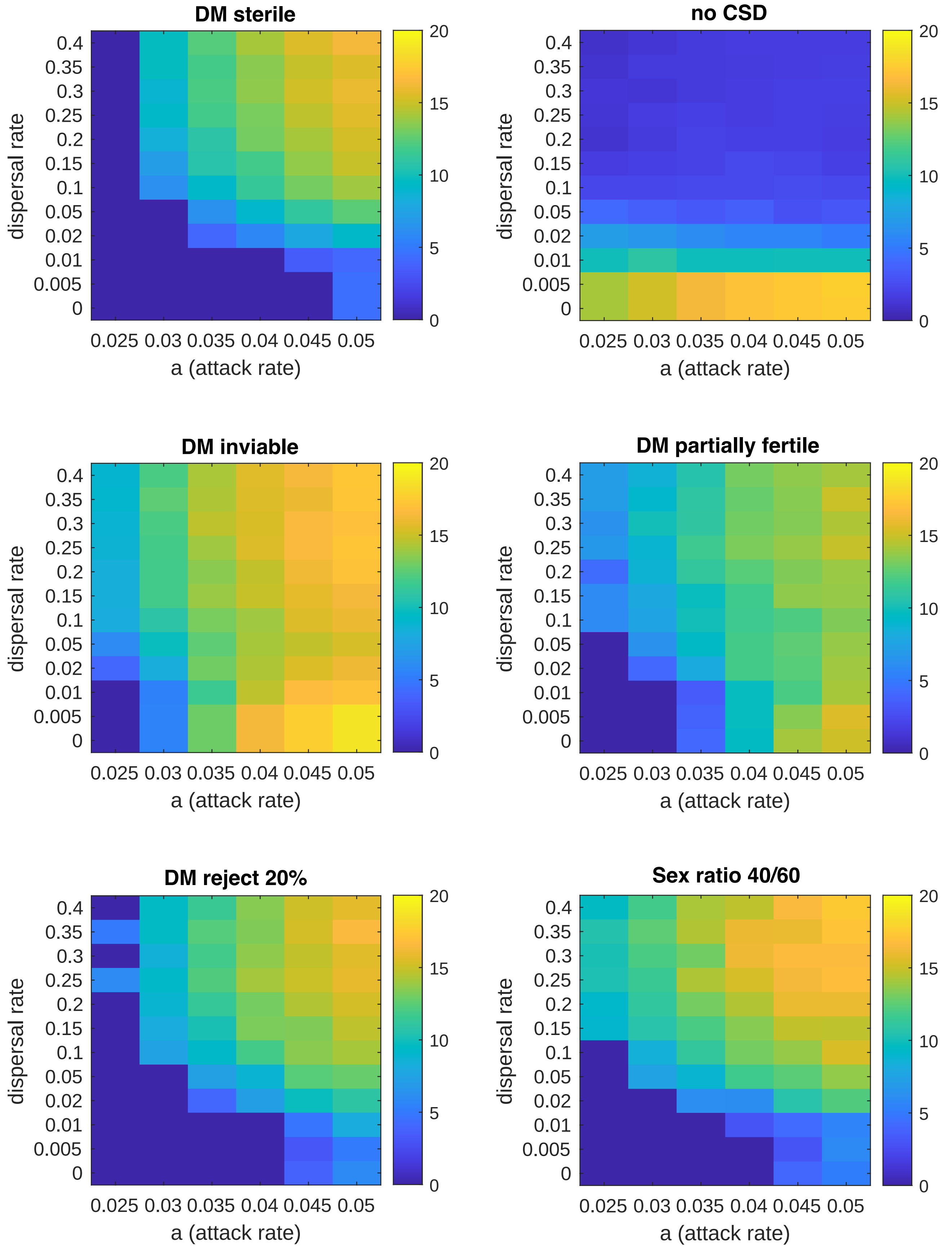

Model 5: small fluctuation
Number of CSD alleles

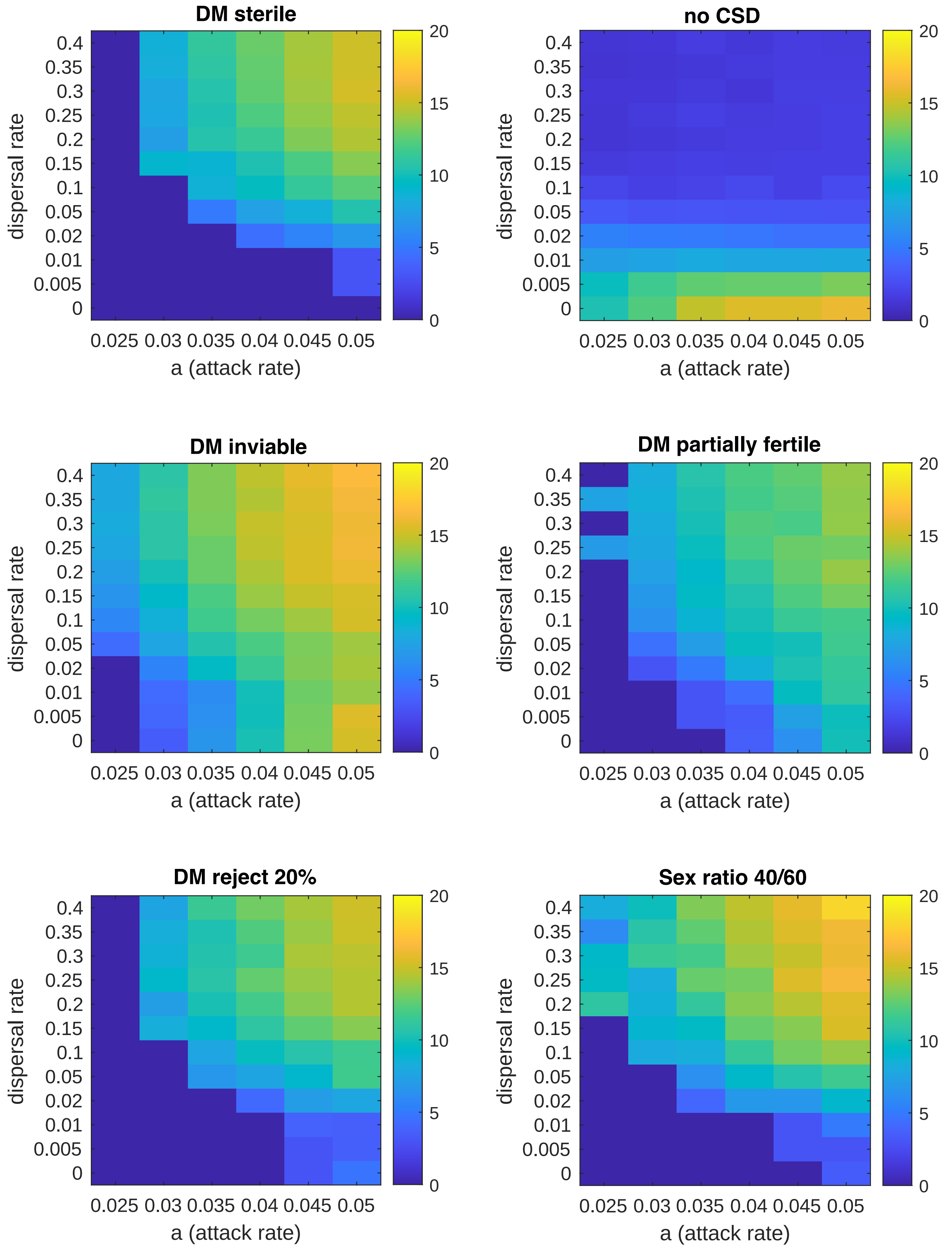

Model 5: large fluctuation
Number of CSD alleles

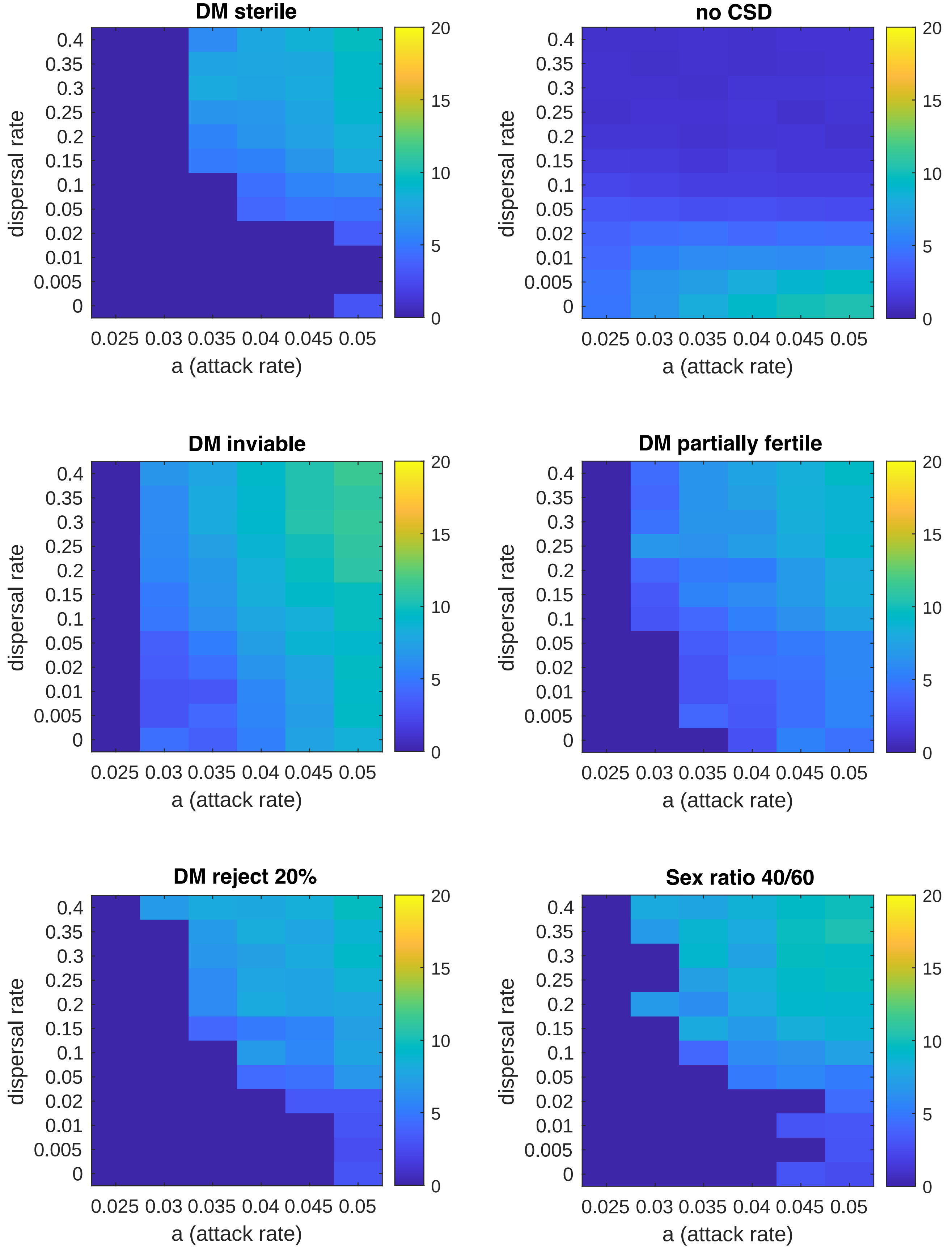

Model 5: spatially autocorrelated large fluctuation
Number of CSD alleles

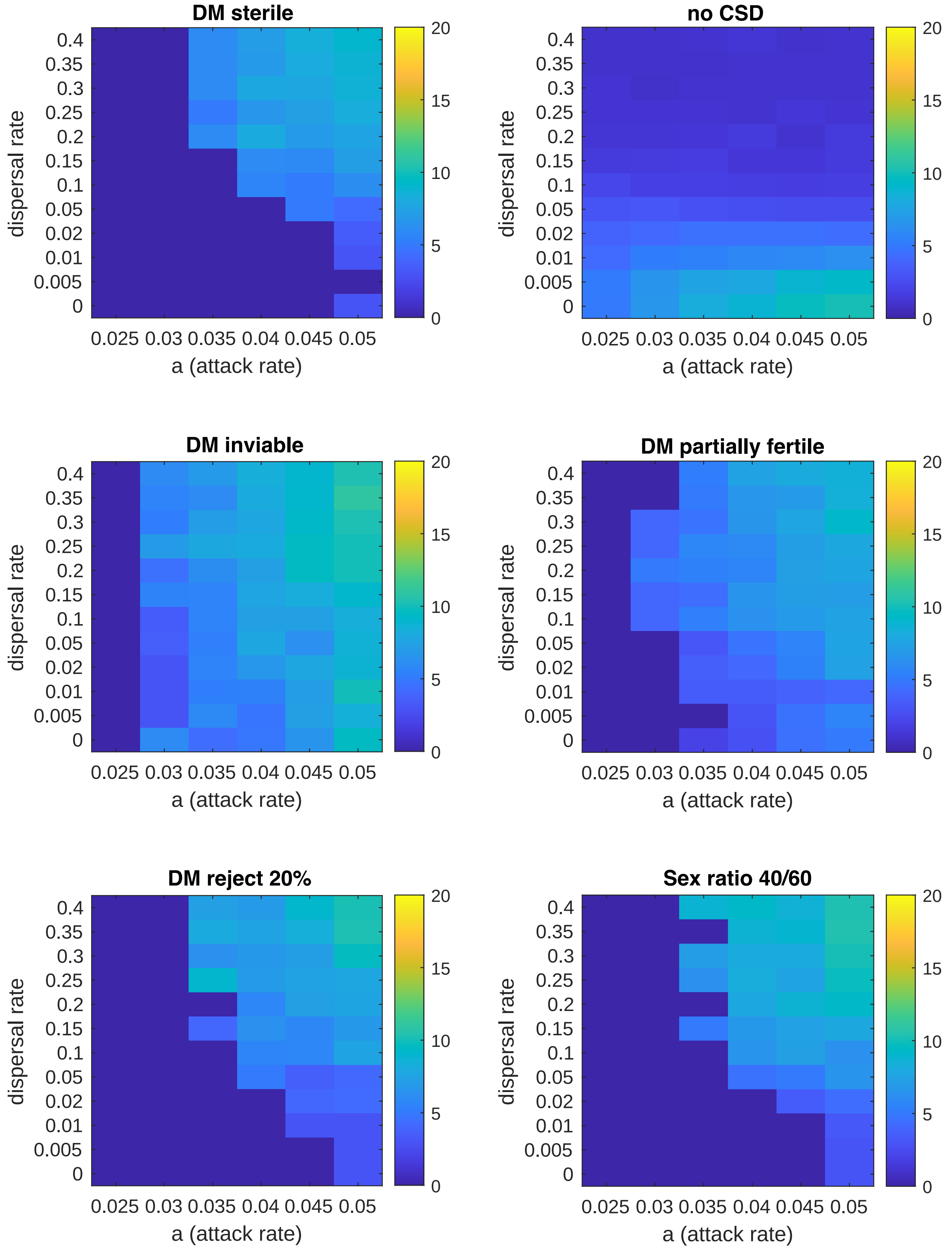

Model 5: large rednoise
Number of CSD alleles

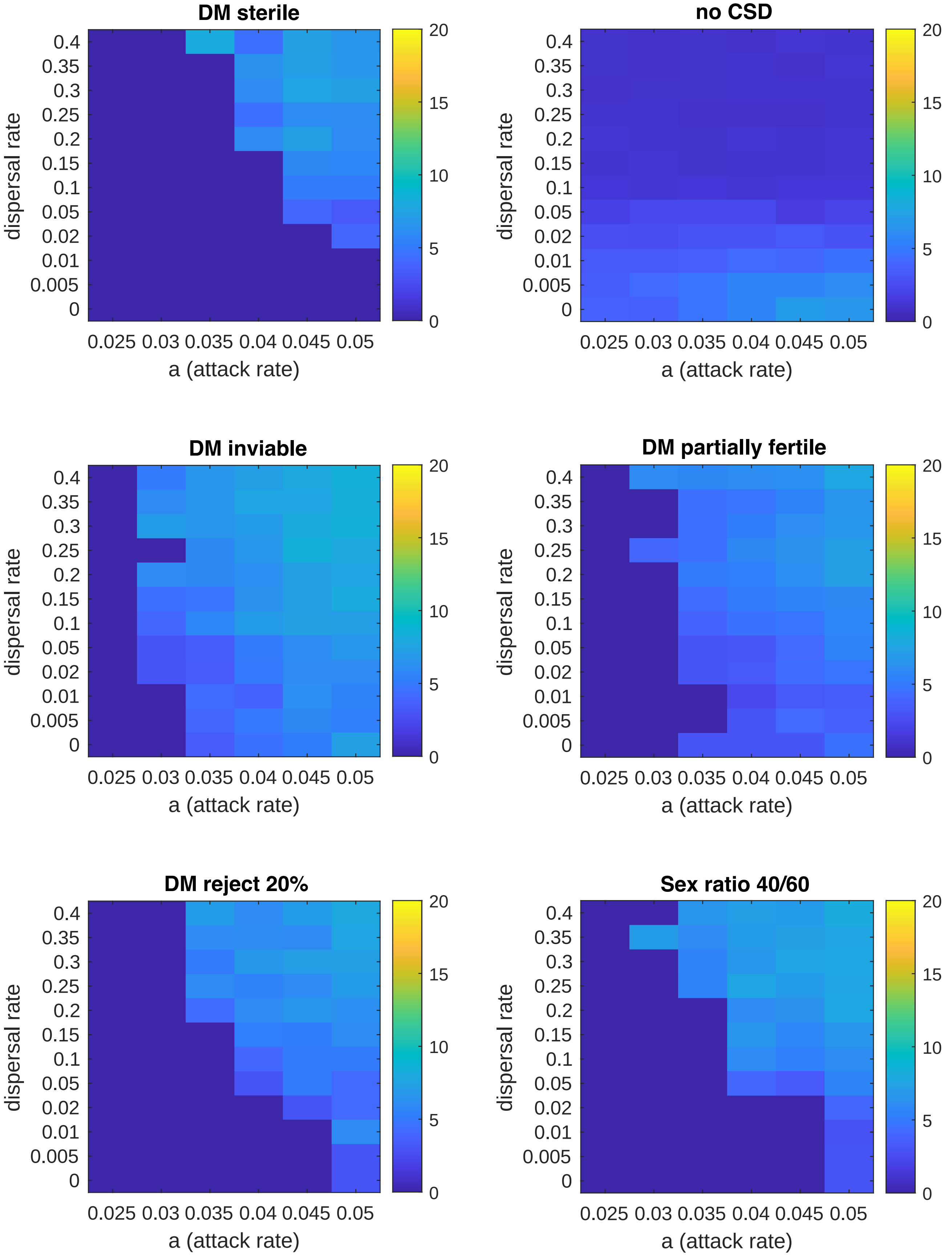

Model 6: no added fluctuation
Number of CSD alleles

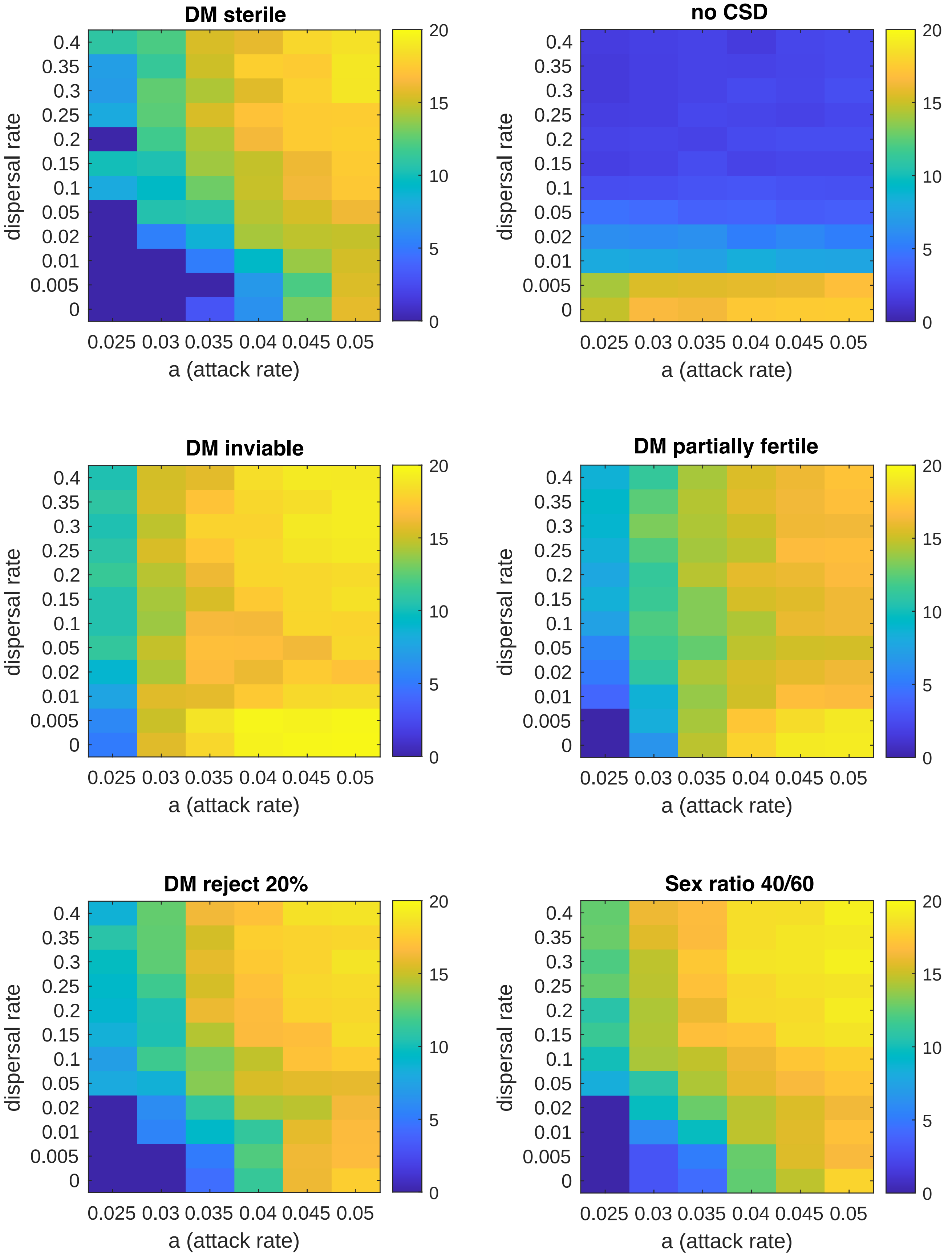

Model 6: small fluctuation
Number of CSD alleles

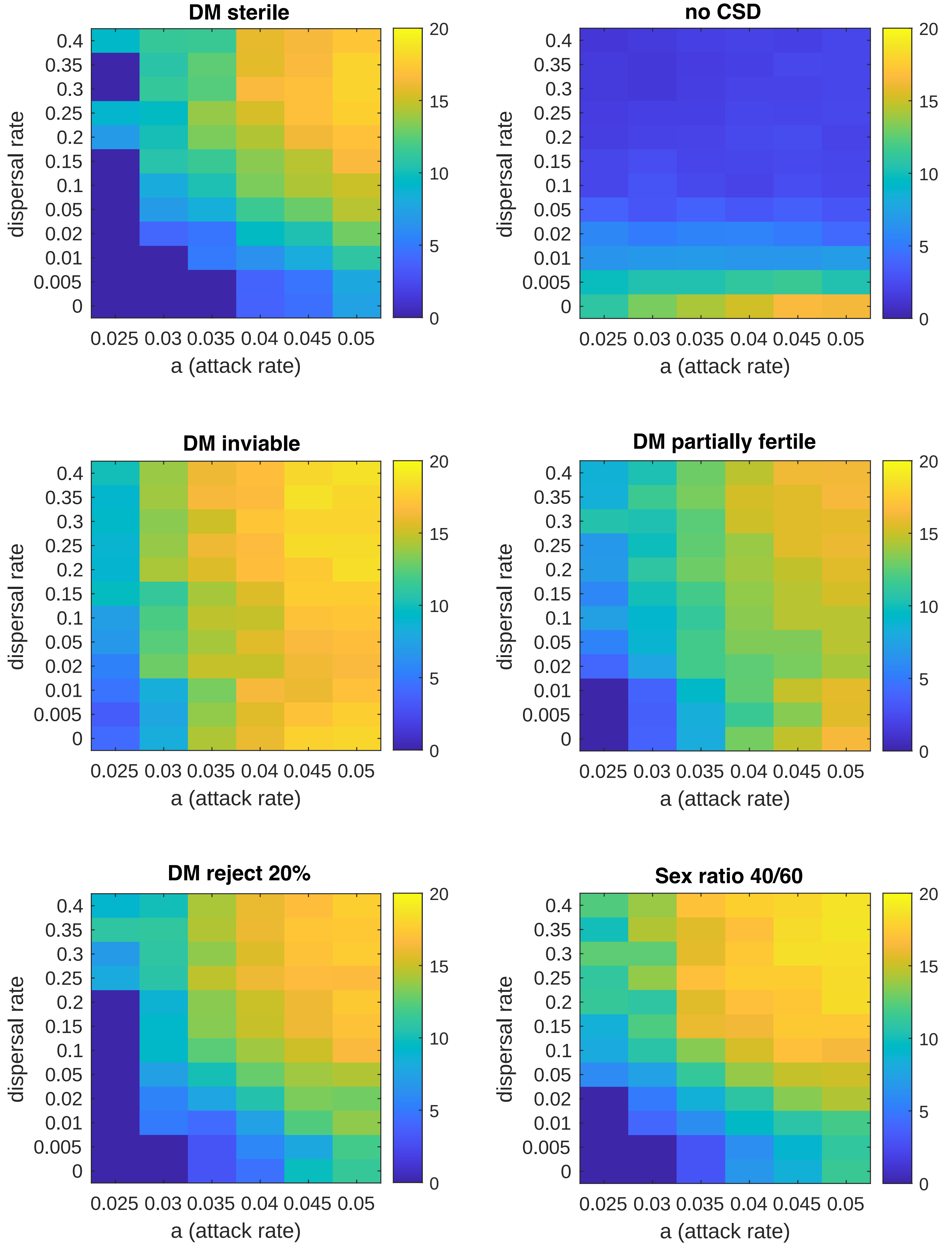

Model 6: large fluctuation
Number of CSD alleles

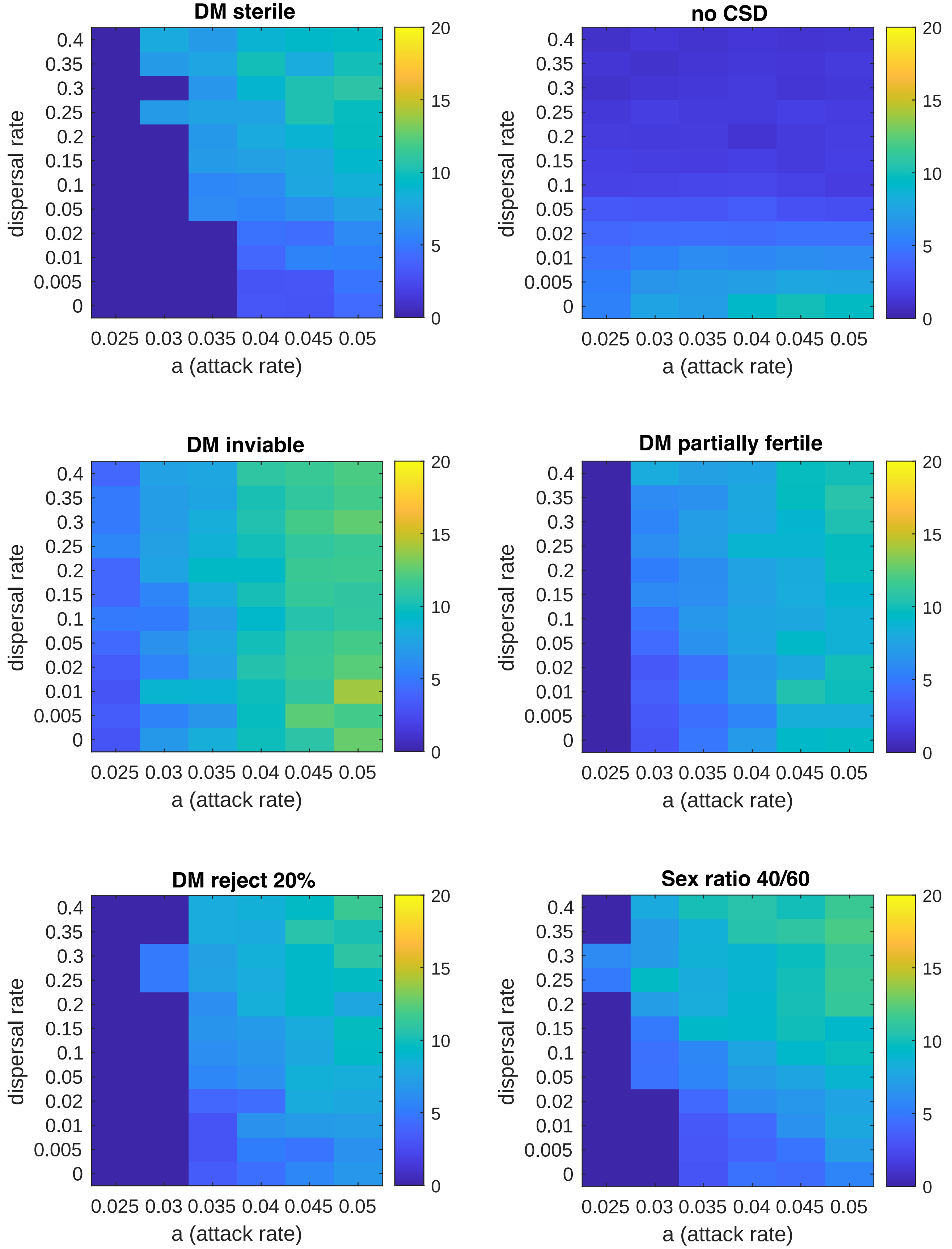

Model 6: spatially autocorrelated large fluctuation

Number of CSD alleles

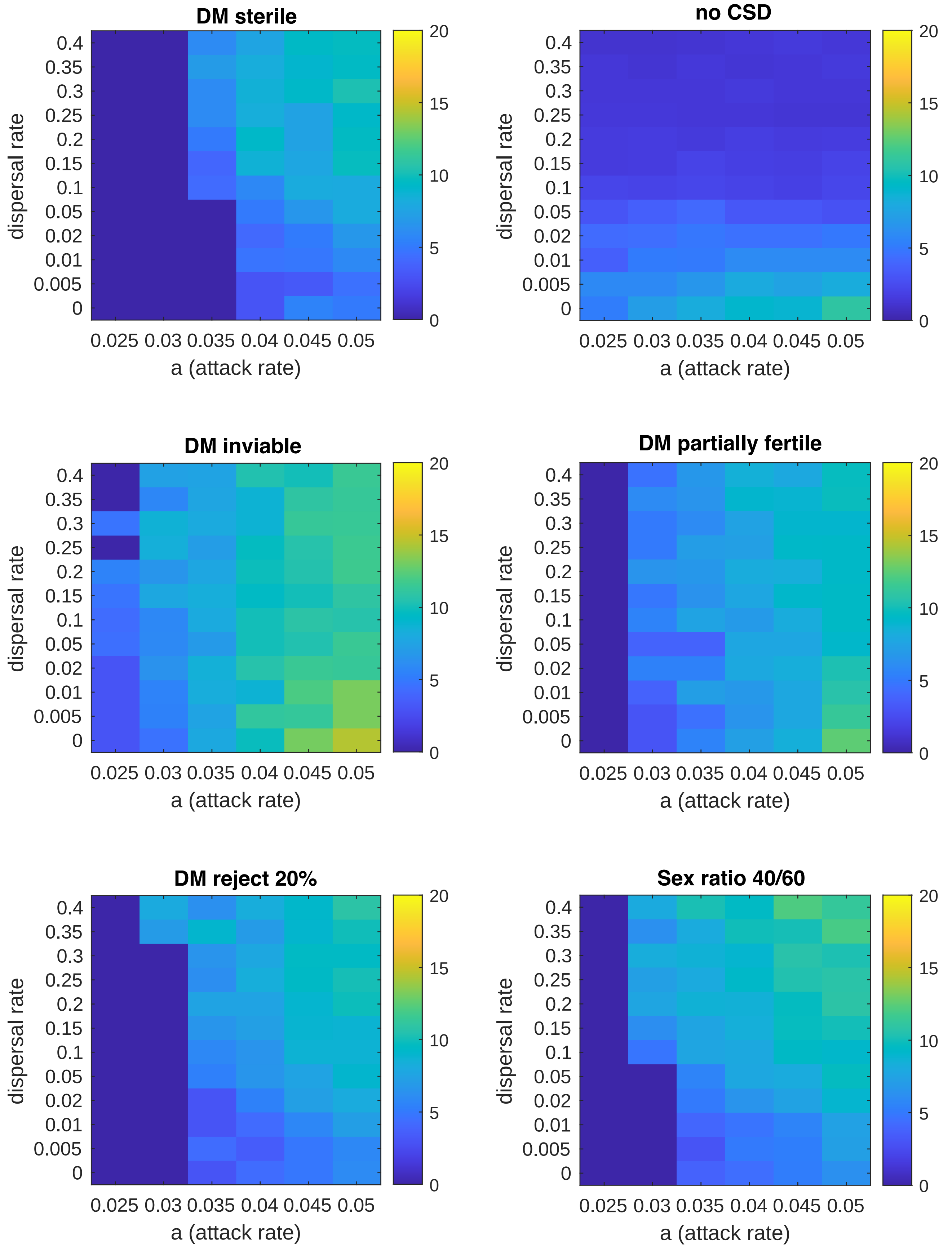

Model 6: large rednoise
Number of CSD alleles

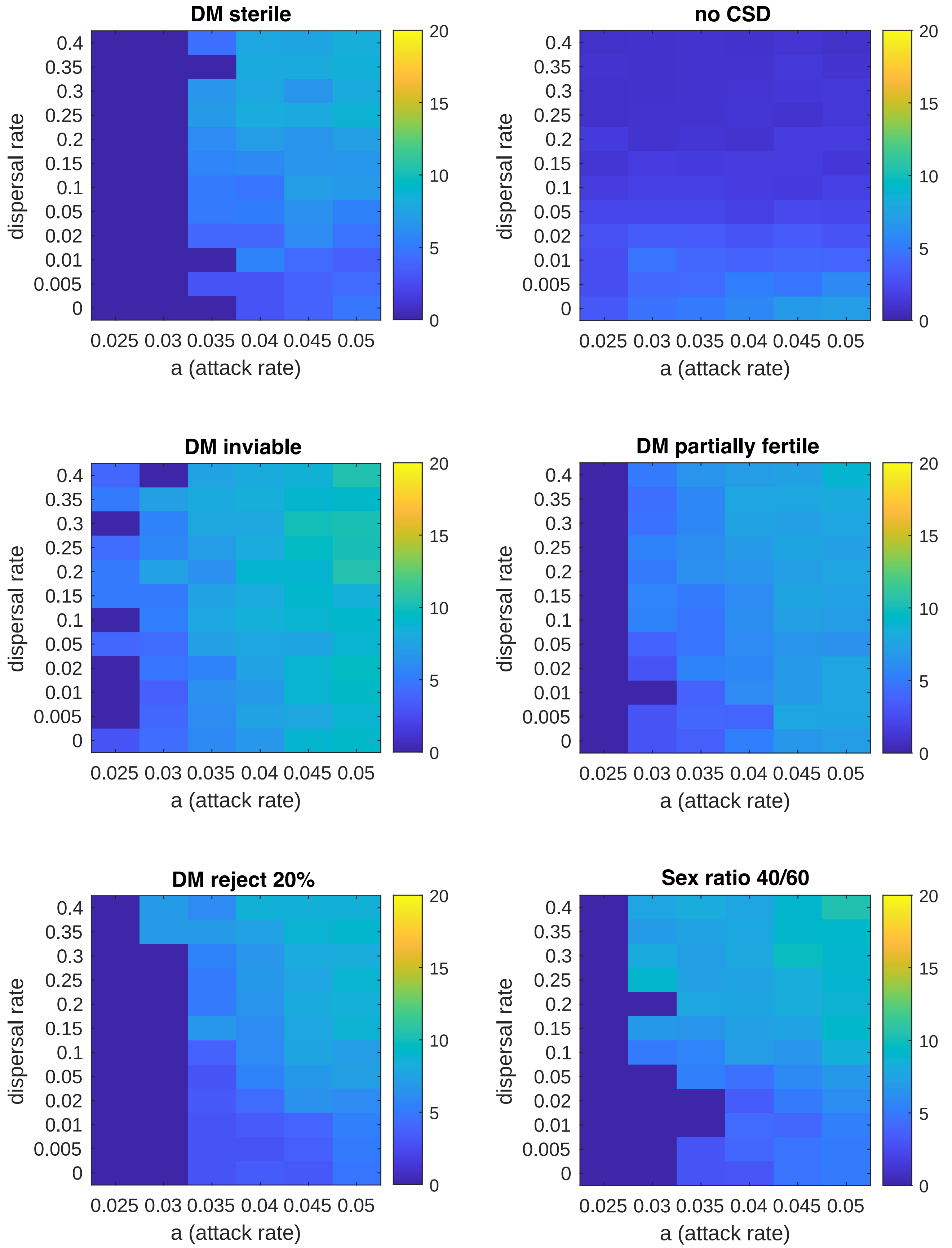

Supplement: Supplementary file 1 — Supplementary Material [file ECE3-10-13030-s001.pdf]
